# Decade Milestone Advancement of Defect-Engineered g-C_3_N_4_ for Solar Catalytic Applications

**DOI:** 10.1007/s40820-023-01297-x

**Published:** 2024-01-04

**Authors:** Shaoqi Hou, Xiaochun Gao, Xingyue Lv, Yilin Zhao, Xitao Yin, Ying Liu, Juan Fang, Xingxing Yu, Xiaoguang Ma, Tianyi Ma, Dawei Su

**Affiliations:** 1 School of Mathematical and Physical Sciences, Faculty of Science, University of Technology Sydney (UTS), Sydney, NSW 2007 Australia; 2 Laboratory of Plasma and Energy Conversion, School of Physics and Optoelectronic Engineering, Ludong University, 186 Middle Hongqi Road, Yantai, 264025 People’s Republic of China; 3 School of Energy and Environmental Engineering, University of Science and Technology Beijing, Beijing, 100083 People’s Republic of China; 4 Department of Chemistry, The University of Tokyo, 7-3-1 Hogo, Bunkyo, Tokyo, Japan; 5 School of Science, STEM College, RMIT University, Melbourne, VIC 3000 Australia

**Keywords:** Defect engineering, g-C_3_N_4_, Electronic band structures, Photocarrier transfer kinetics, Defect states

## Abstract

Over the past decade, graphitic carbon nitride (g-C_3_N_4_) has emerged as a universal photocatalyst toward various sustainable carbo-neutral technologies. Despite solar applications discrepancy, g-C_3_N_4_ is still confronted with a general fatal issue of insufficient supply of thermodynamically active photocarriers due to its inferior solar harvesting ability and sluggish charge transfer dynamics. Fortunately, this could be significantly alleviated by the “all-in-one” defect engineering strategy, which enables a simultaneous amelioration of both textural uniqueness and intrinsic electronic band structures. To this end, we have summarized an unprecedently comprehensive discussion on defect controls including the vacancy/non-metallic dopant creation with optimized electronic band structure and electronic density, metallic doping with ultra-active coordinated environment (M–N_*x*_, M–C_2_N_2_, M–O bonding), functional group grafting with optimized band structure, and promoted crystallinity with extended conjugation *π* system with weakened interlayered van der Waals interaction. Among them, the defect states induced by various defect types such as N vacancy, P/S/halogen dopants, and cyano group in boosting solar harvesting and accelerating photocarrier transfer have also been emphasized. More importantly, the shallow defect traps identified by femtosecond transient absorption spectra (fs-TAS) have also been highlighted. It is believed that this review would pave the way for future readers with a unique insight into a more precise defective g-C_3_N_4_ “customization”, motivating more profound thinking and flourishing research outputs on g-C_3_N_4_-based photocatalysis.

## Highlights

- This review summarizes the decade milestone advancement of defect-engineered g-C_3_N_4_ and emphasizes the roles of crystallinity and defect traps toward a more precise defective g-C_3_N_4_ “customization” in the future.
- A critical insight into the defect traps has been discussed in depth, probing the defect-induced states and photocarrier transfer kinetics of g-C_3_N_4_.
- The prospect and outlooking for precise defective g-C_3_N_4_ “customization” is proposed.

## Graphical abstract

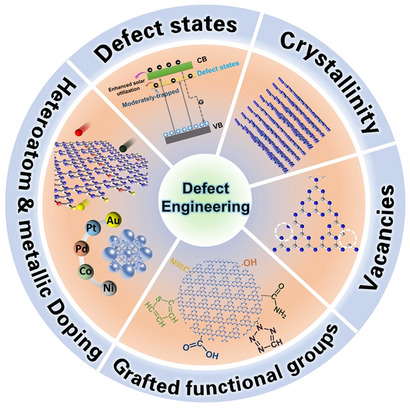

## Introduction

**Fig. 1:**
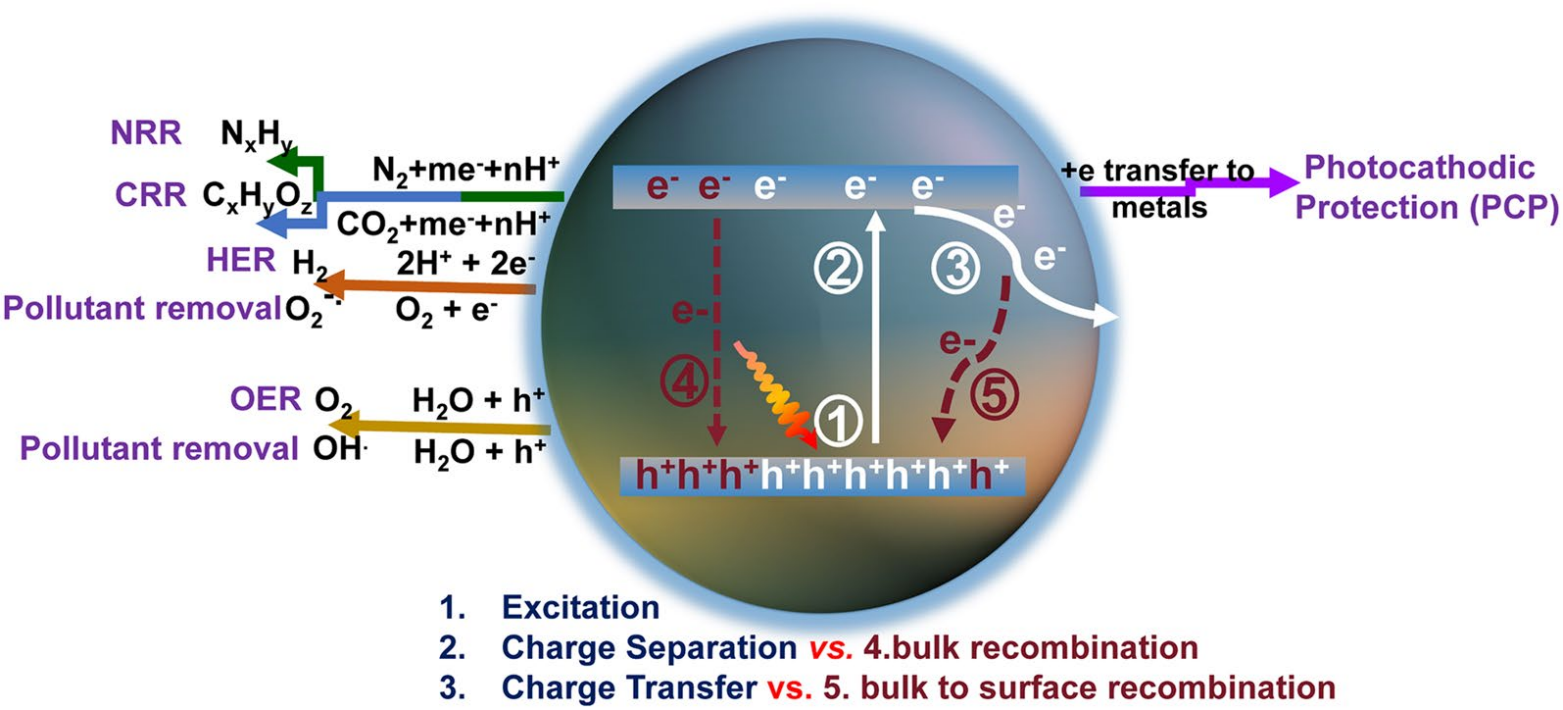
Schematic photoexcitation, charge transport, and solar applications for g-C_3_N_4_

Solar-to-chemicals/electricity oriented by photocatalysts has been regarded as a promising supplement for existing energy types [1–13]. Nowadays, the emerging graphitic carbon nitrides (g-C_3_N_4_) have attracted numerous research attention [14, 15], outperforming the traditional TiO_2_ materials, particularly in the research fields of solar-driven H_2_ evolution reaction (HER) [16–19], CO_2_ reduction reaction (CRR) [20–26], N_2_ reduction reaction (NRR) [27–32], photocathodic protection (PCP) [33–37], pollutant removal [38–41], and oxygen evolution reaction (OER) [42–45]. Despite the application discrepancy, they all share similarities until the electrons are involved in redox reactions in an aqueous solution [5, 46]. Specifically, this progress in g-C_3_N_4_ materials can be classified into 5 steps (Fig. 1): (1) When the irradiation energy is larger than the bandgap (typically around 2.7 eV) [7, 47], the electrons and holes in g-C_3_N_4_ can be excited; (2) once irradiation, the electrons in valance band maximal (VBM: 1.57 V vs. standard hydrogen electrode (SHE)) would be excited into conductive band maximal (CBM: − 1.13 V vs. SHE), leaving the VB occupied with holes (oxidizing ability) and CB with electrons (reducing ability), respectively. (3) Afterward, the electrons would transfer from bulk to surface and finally reach the active interfacial sites to participate in the redox reaction. It is worth mentioning that the CB position in g-C_3_N_4_ must be more negative than the desired reduction potential so that the reductive reactions (HER, CRR, NRR, pollutant removal, PCP) can take place. Similarly, the VB position should be more positive than the required oxidation potential to satisfy the oxidation reactions such as OER and ·OH generation. However, the separation and transport of photocarriers (electrons and holes) in both bulk-phase and surface of g-C_3_N_4_ are not smooth as there are mainly two recombination pathways: (4) The excited electrons in CB are very active and prone to recombine with holes in VB, mainly releasing energy with the radiative fluorescence; (5) The electrons migrated from CB to surface are also susceptible to be trapped by the defect-associated surface states and then recombine with holes, releasing energy in a non-radiative way with heat.

## Challenges

Since the pioneered work on the discovery of g-C_3_N_4_ for photocatalytic H_2_ evolution by Wang et Al. [7], g-C_3_N_4_ has emerged as a hot metal-free photocatalyst with environmental benignity that attracts numerous research attention. Despite the various photocatalytic applications, g-C_3_N_4_ is still confronted by the above-mentioned five fundamental steps, of which the initial photoexcitation followed by photocarrier transfer processes are quite complex. Specifically, we summarize the most intractable challenges that impede the large-scale applications of g-C_3_N_4_. On the charge excitation side, the challenge is:

1. **Insufficient solar light absorption** Photoexcitation acts as the primary and fundamental step for solar applications of g-C_3_N_4_, of which if there are more excited photocarriers, there would be more efficient photocarriers involved in the final redox reaction. So far, enormous efforts to create a more abundant specific surface area with porous nanostructures to enhance the multiple solar scattering, diffraction, and absorption have been demonstrated to be efficient in boosting the corresponding photocatalytic activity of g-C_3_N_4_ [48–54]. However, the bandgap of bulk g-C_3_N_4_ is around 2.7 eV which means the hole/electron pairs can only be excited under light wavelength shorter than 460 nm, which occupies only around 16.5% of the solar spectrum. The low absorption of longer visible light longer than 460 nm and even near-infrared light leads to a limited amount of photoexcited electrons and holes, which would dramatically lower the solar activity of g-C_3_N_4_.
  While on the charge transport side, the challenges are more complicated, which include:
2. **Sluggish photocarrier transfer kinetics** For the lowest unoccupied molecular orbital (LUMO) of pristine g-C_3_N_4_, no electrons appeared around the bridging N atoms, which indicates the electron in g-C_3_N_4_ would only be excited and transferred within one C_6_N_7_ unit, thus hindering the electron transfer along the in-plane direction and increasing the photocarrier possibility of being recombination [55]. To this end, the intrinsic localized *π* conjugated network of g-C_3_N_4_ leads to slow photocarrier mobility with low electronic conductivity and sluggish photocarrier transfer kinetics in the horizontal direction. In addition, the insufficient polymerization degree of g-C_3_N_4_ also generates edged amino groups which could act as charge traps, further hindering the photocarrier transfer in the vertical direction. Therefore, both situations can lead to a sluggish charge transfer process, thus fewer electrons or holes presenting in the interfacial surface of g-C_3_N_4_.
3. **Severe photocarrier recombination in bulk-phase** The excited active electrons in CB are not in thermodynamical equilibrium and thus prone to return to the ground state, releasing energy via a non-radiative transition. This is particularly true for bulk g-C_3_N_4_ as the severe electron localization has largely restrained the photocarrier transfer rate, leaving a longer time for photocarrier bleaching. Additionally, there are no additional energy levels between CBM and VBM, lacking the temporary photocarrier “reservoir” to buffer the photoexcited electrons or holes from CBM and VBM, respectively. Therefore, the photogenerated electrons and holes cannot be separated efficiently, resulting in insufficient photocarrier for redox reactions, which needs to be urgently improved.
4. **Severe photocarrier recombination in the surface** As we know, the intensive pyrolysis of melamine or other precursors would lead to the NH_3_ gas, and the active H* during the heat treatment can induce a considerable portion of edge amino groups, which lowers the polymerization degree. As g-C_3_N_4_ is a N-containing material, the presence of amino groups would inevitably induce a relatively strong interlayered van der Waals interaction, which is prone to become the surface traps to bleach the photocarriers. Furthermore, if the experimental condition contains impurities, there also might be a bigger possibility to induce more surface traps. The surface recombination would happen in a less-easy detected manner, releasing the recombination energy in a non-radiative way of heat. However, this point has less been emphasized in comparison with the former bulk-phase recombination, which needs to be alleviated in the next studies on photocarrier transfer dynamics and g-C_3_N_4_-based photocatalytic activities.

## Solution: Defect Engineering

Defect engineering refers to the introduction of impurities to the matrix or regulation of atom periodicity of semiconductors, which has been successfully proven to be an efficient strategy in tailoring the electronic band structures, optical properties, and conductivity of photocatalysts [56–62]. Intriguingly, apart from the intrinsic merits changes, the extrinsic morphology of g-C_3_N_4_ can also be optimized in terms of precursor types, reaction templates, and annealing conditions (pyrolysis atmosphere, heating rate, annealing time, and pressure). As a result, the defective g-C_3_N_4_ samples normally enable significant improvements in extended solar harvesting ability, efficient photocarrier transfer process, as well as higher surface area with more abundant active sites, thus leading to a comprehensive activity increase for various photocatalytic applications. To this end, we believe defect engineering could be regarded as an “all-in-one” strategy to boost the solar utilization of g-C_3_N_4_ as it takes the most important factors, namely the electronic band structure and nanostructure into consideration toward various photocatalytic applications.

Despite great achievements have been made in boosting the solar activity of g-C_3_N_4_ via morphology modification [48–54] and hybrid construction [63–74], the electronic band structure and photocarriers transfer in bare g-C_3_N_4_ should be emphasized as they are the basement for further performance enhancement. Fortunately, these drawbacks of g-C_3_N_4_ have been demonstrated to be significantly ameliorated via a defect engineering strategy. Defect engineering refers to the introduction of impurities to the matrix or regulation of atom periodicity of semiconductors, which has been successfully proven to be an efficient strategy in tailoring the electronic band structures, optical properties, and conductivity of photocatalysts [56–62]. Intriguingly, apart from the intrinsic merits changes, the extrinsic morphology of g-C_3_N_4_ can also be optimized in terms of precursor types, reaction templates, and annealing conditions (pyrolysis atmosphere, heating rate, annealing time, and pressure). As a result, the defective g-C_3_N_4_ samples normally enable significant improvements in extended solar harvesting ability, efficient photocarrier transfer process, as well as higher surface area with more abundant active sites, thus leading to a comprehensive activity increase for various photocatalytic applications. To this end, we believe defect engineering could be regarded as an “all-in-one” strategy to boost the solar utilization of g-C_3_N_4_ as it takes the most important factors, namely the electronic band structure and nanostructure into consideration toward various photocatalytic applications.

**Fig. 2:**
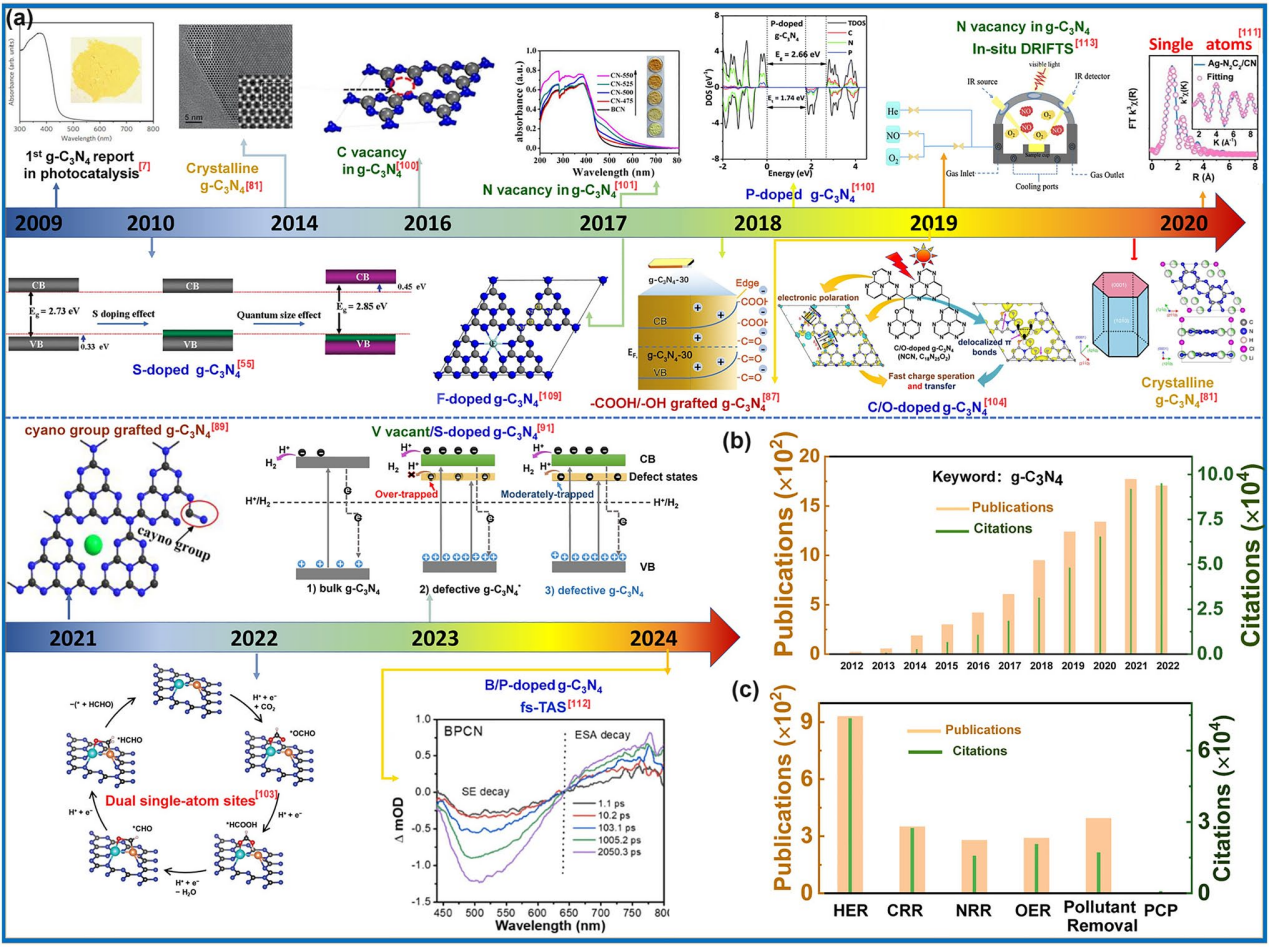
**a** Research history outline of defective g-C_3_N_4_ photocatalysts for solar applications [7, 55, 81, 87, 89, 91, 100, 101, 103, 104, 109–113]; Number of annual publications and citations **b** using “g-C_3_N_4_” as the title from 2012 to 2022 and **c** using “g-C_3_N_4_” plus “hydrogen evolution”, or “CO_2_ reduction”, or “nitrogen reduction”, or “oxygen evolution”, or “pollutant removal”, or “photocathodic protection” as topics in 2022. Adapted from ISI Web of Science, dated 8th June 2023

Throughout the research history of self-modified defective g-C_3_N_4_ [7, 75–99], there exists various defect types including the C/N vacancies [100, 101], heteroatom dopants [102], metallic dopants [103], grafted functional groups [89] as well as crystallinity improvement [83] toward the solar-driven HER, CRR, NRR, OER, PCP, and pollutant removal applications as reflected by the surging publications and citations since 2012 (Fig. 2). Generally speaking, with these defect modification strategies, the bandgaps of g-C_3_N_4_ can be dramatically reduced, rendering an enhanced solar harvesting ability even to almost 600 nm [102]. While for the N vacancies [101] or heteroatomic doping with higher electronegativity atoms such as P/S/F [102], there might be new energy levels (defect states) lying in the forbidden bands or strong electronic polarization effect, respectively. For instance, the O-/S/F-doped g-C_3_N_4_ would induce an electron redistribution and electronic polarization [104], even with the electrons accumulating around the bridging N sites in the HOMO and LOMO, leading to a faster charge transfer kinetics [55]. Additionally, Gao et al. proposed an N vacant and S-doped g-C_3_N_4_ with shallow defect states, which enabled a higher photocatalytic HER rate of 4,219.9 μmol g^−1^ h^−1^, which was 29.1-fold higher than unmodified g-C_3_N_4_ [91]. The shallow defect states could act as a temporary electron reservoir to accommodate the electrons from CBM, suppressing the bulk-phase photocarrier recombination. The Co single atoms (SAs) were successfully doped into the g-C_3_N_4_ matrix forming the Co–N bonding via a microwave method to promote the CO yield achieved the highest value of 1.056 μmol mg^−1^ [105]. In addition, the crystalline g-C_3_N_4_ with cyano groups also exhibited a high photocatalytic HER of 64 μmol h^−1^ as its enhanced charge transfer rate and optimized photocarrier separation [106].

The topic of our review is unique as it focuses on the self-defect engineering of g-C_3_N_4_, limiting the range out of morphology control, heterostructures, and coupling compounds [107, 108]. Firstly, we introduce the challenges confronted by bulk g-C_3_N_4_, mainly including insufficient solar light absorption (particularly the longer wavelength than 450 nm) and the inferior photocarrier separation efficiency in both bulk-phase and surface. Compared with other reviews on one or several defect types [62], this review is a more comprehensive view as it includes all kinds of defect controls including vacancy creation, non-metal/metallic doping, functional groups grafting, particularly crystallinity enhancement, and defect traps, which have been discussed as the solutions of defect engineering. Importantly, theoretical guidance in understanding defect roles and redox mechanism, emphasis on the defect states, and probing of photocarrier kinetics by the introduction of femtosecond transient spectrum have also been throughout reviewed. Last but not least, the limits and outlook of defective g-C_3_N_4_ have been proposed to bring more comprehensive insights for the ultimate goal of defect “customization” for future readers.

### Design Principles of Defect Engineering

**Fig. 3:**
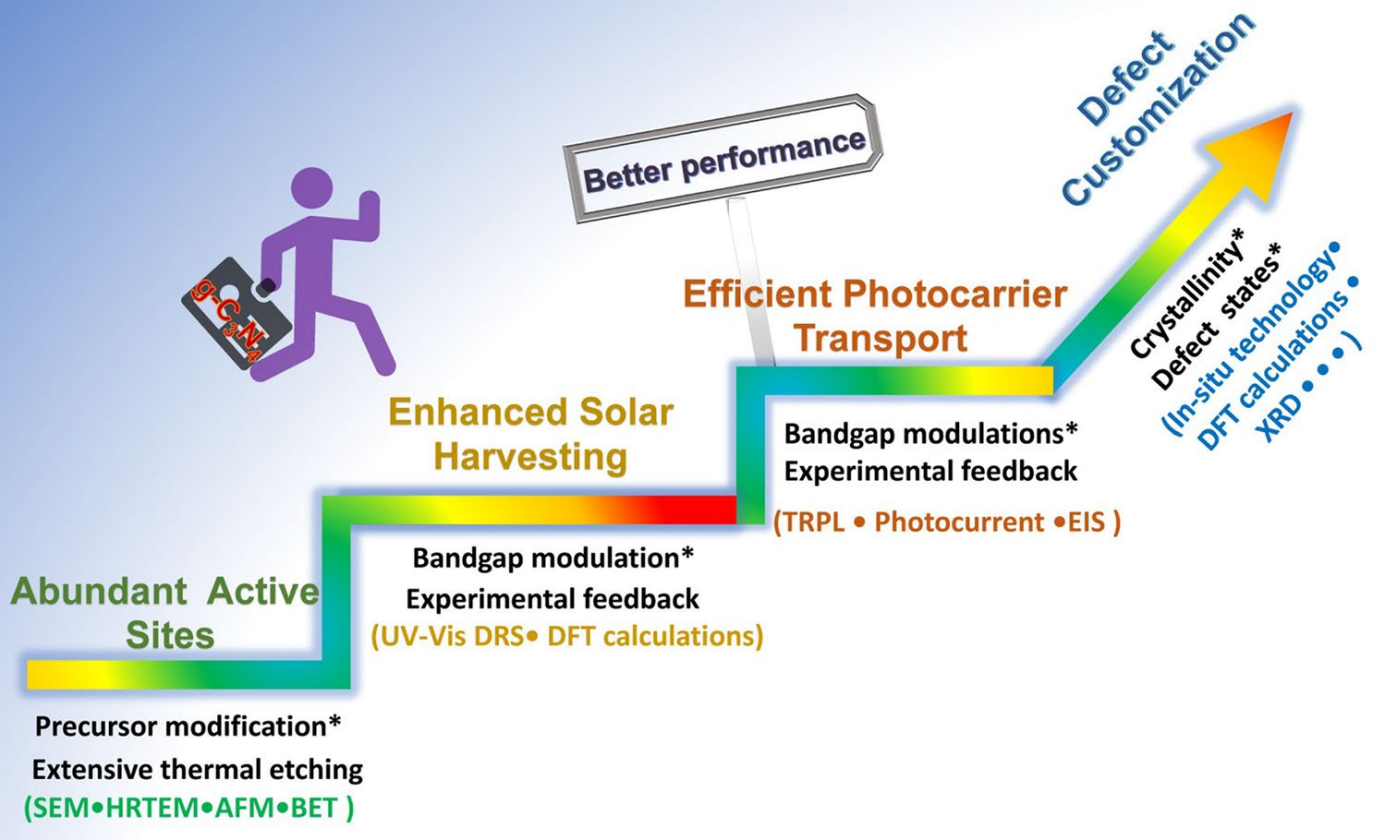
Design principles of defective g-C_3_N_4_ toward better photocatalytic performance

In general, the defect engineering on g-C_3_N_4_ should obey three important principles, namely the basic creation of abundant active sites, enhanced solar harvesting ability, and efficient transport (Fig. 3). (i) For the former abundant active sites, the synthetic strategy mainly focuses on the precursor modification along with the thermal etching at desired gas atmospheres. The scanning electron microscopy (SEM), transmission electron microscopy (TEM), atomic force microscopy (AFM), and Brunauer–Emmett–Teller (BET) techniques have been used to characterize the corresponding porous structures. (ii) For the enhanced solar harvesting requirement, the bandgap calculated via UV–visible diffuse reflectance spectra (UV/Vis DRS) should be optimized with experimental feedback. Importantly, density functional theory (DFT) calculations are a good guidance tool to learn the defect merits. (iii) As for the latter efficient photocarrier transport, time-resolved fluorescence spectroscopy (TRPL), photocurrent, and electrochemical impedance spectroscopy (EIS) are powerful tools to evaluate the extent of photocarrier separation efficiency by getting the lifetimes, photocurrent, and trapping resistance results. It can also be optimized by those approaches of solar harvesting. To achieve the ultimate goal of defective g-C_3_N_4_ with the best performance, more research work needs to be carried out in the near future, including the precise control of crystallinity, defect states with shallow positions or even optimized surface states. To achieve the defect customization, more advanced in situ probing technologies are also required such as the in situ diffuse reflectance infrared Fourier transform spectrums (DRIFTS), and in situ Femtosecond transient spectrums.

### Vacancies with Optimized Band Structures and Electronic Density

**Fig. 4:**
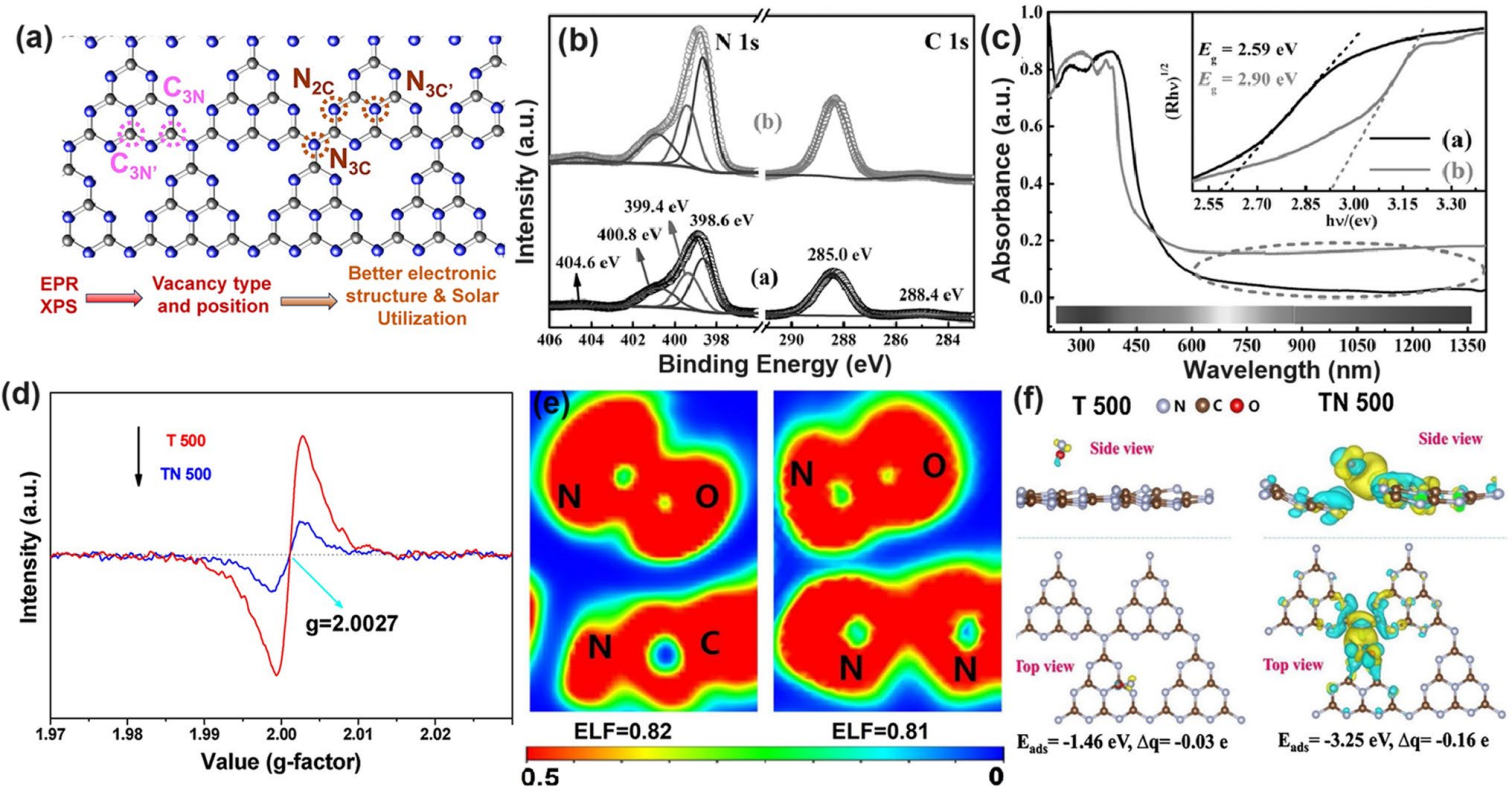
Defect control of C_3N_ vacancies.** a** Possible C vacant and N vacant positions in g-C_3_N_4_; **b** high-resolution C 1* s* and N 1* s* XPS spectra; **c** DRS spectra and Tauc plot [115]. Copyright 2015, Wiley–VCH. **d** Electron paramagnetic resonance (EPR) spectra; **e** ELF diagrams (left: T 500; right: TN500); **f** adsorption energy and charge density difference between T 500/TN 500 and NO [116]. Copyright 2020, Elsevier

By changing the experimental conditions for g-C_3_N_4_ synthesis, either C vacancies or N vacancies can be obtained, of which the vacancy type and position can be identified by the electron paramagnetic resonance (EPR) signal and resolved X-ray photoelectron spectroscopy (XPS) peak area ratios of C or N species. Generally speaking, both C and N vacancies could impart g-C_3_N_4_ with an optimized electronic structure, including a narrower bandgap, enhanced solar light absorption, and more favorable charge separation and transport, thus rendering an improvement of solar utilization (Fig. 4a). Based on the geometrical configuration, the C vacancies only occur in the three-coordinated sites with the edge and inner sites to be C_3N_ and C_3N’_, respectively. While for N vacancies, the vacant positions can be classified into two-coordinated N sites (N_2c_), inner three-coordinated N sites (N_3c’_), and outer three-coordinated N sites (N_3c_), respectively. With the growing knowledge of theoretical calculations, the formation of defect states and the reaction mechanism for vacant g-C_3_N_4_ have also been discussed in depth. It is worth mentioning that the C vacancies preferred to induce a delocalized *π* bonding at the bridging N_3C_ sites [104], thus boosting the electrons transferring between different C_6_N_8_ units. In this way, the photocarrier transport would be more efficient, giving rise to an overall photocatalytic performance enhancement. While the N vacancies might work in a different way additional energy levels (C_3_^+^, defect states) would be induced in the forbidden band [114]. These defect states could play a positive role in that, on one hand, the band excitation energy can be reduced with enhanced solar harvesting ability. On the other hand, these states could accommodate the migrated electrons from CBM, suppressing the photocarrier recombination process. Benefiting from the above-mentioned factors, the N vacant g-C_3_N_4_ generally delivers a substantially promoted photocatalytic activity [91]. Therefore, this section would start with C vacancies, then N vacancies, and finally both C vacancies and N vacancies in one g-C_3_N_4_ material.

#### C Vacancies with Enhanced Electronic Polarization

An initial work on C vacant g-C_3_N_4_ was reported by Yang’s group [115]. Specifically, the porous holy C vacant g-C_3_N_4_ nanosheets (HGCN) were obtained by the thermal exfoliation of bulk g-C_3_N_4_ (BGCN) under NH_3_ atmosphere. Compared to pristine BGCN, HGCN owned plentiful in-plane pores that were more accessible to aqueous solution and reduced van der Waals interaction, which could significantly enhance the mass transport and photocarrier separation for water splitting. However, the author claimed that the C vacancies might originate from the loss of graphitic C species according to the increased peak-area ratio of N=C–N to C=C from 0.13 to 0.14 for BGCN and HGCN, respectively (Fig. 4b). This explanation could be fuzzy because the C=C peak for both samples was not obvious, and this weak signal might also come from the equipment or sample contamination. Different from our expectation, HGCN showed an enlarged bandgap of 2.90 eV that was 0.31 eV higher than BGCN (Fig. 4c), which was supposed to be the quantum confinement effect owing to the small grain size and ultra-thin merits of HGCN nanosheets. Additionally, the authors also insisted that, owing to the C vacancies, the enhancement of light absorption in HGCN in the near-infrared region could also be witnessed. Benefiting from the above-mentioned factors, this C vacant g-C_3_N_4_ exhibited a prolonged charge lifetime and enhanced HER rate, which was 1.7 and 20-fold higher than bulk g-C_3_N_4_.

Different from the above study, Li et al*.* fabricated the tubular g-C_3_N_4_ with C vacancies presented in the edge C_3N_ site via the pyrolysis of urea and melamine mixture under an inert N_2_ atmosphere, and the corresponding products were labeled as TN−*x* (*x* presents annealing temperature) [116]. The authors claimed that the N_2_ atmosphere was critical for the g-C_3_N_4_ morphology and defects generation. Especially, g-C_3_N_4_ obtained without N_2_ (T 500) displayed a tubular length of 20 μm and diameter of 1–2 μm and bulky nanoplates inside. In contrast, TN 500 was observed with thin nanosheets inside, which further demonstrated the exfoliation process induced by the N_2_ atmosphere. In addition, the Lorentzian line for TN 500 was considerably attenuated, confirming the appearance of C vacancies (Fig. 4d). According to the DFT calculations, both samples exhibited a strong covalent interaction with NO due to the high electronic location function (ELF) value of around 0.81 eV (Fig. 4e), indicating the stronger electronic polarization effect due to the absence of C vacancies. Furthermore, the NO molecules can be more easily activated by TN 500 as reflected by its larger adsorption energy and carried total charge (Δ*q*) of − 3.25 eV and 0.16 e, which are 1.79 eV and 0.13 e higher than those of T 500, respectively (Fig. 4f). This demonstrated that the NO molecules were extremely easy to be absorbed and activated over TN 500 than T 500, which would facilitate the redox kinetics of NO photo-oxidation activity. Therefore, TN 500 showed the highest removal rate of NO at 47.7%, implying the superior role of surface C vacancy in accelerating the NO removal rate.

**Fig. 5:**
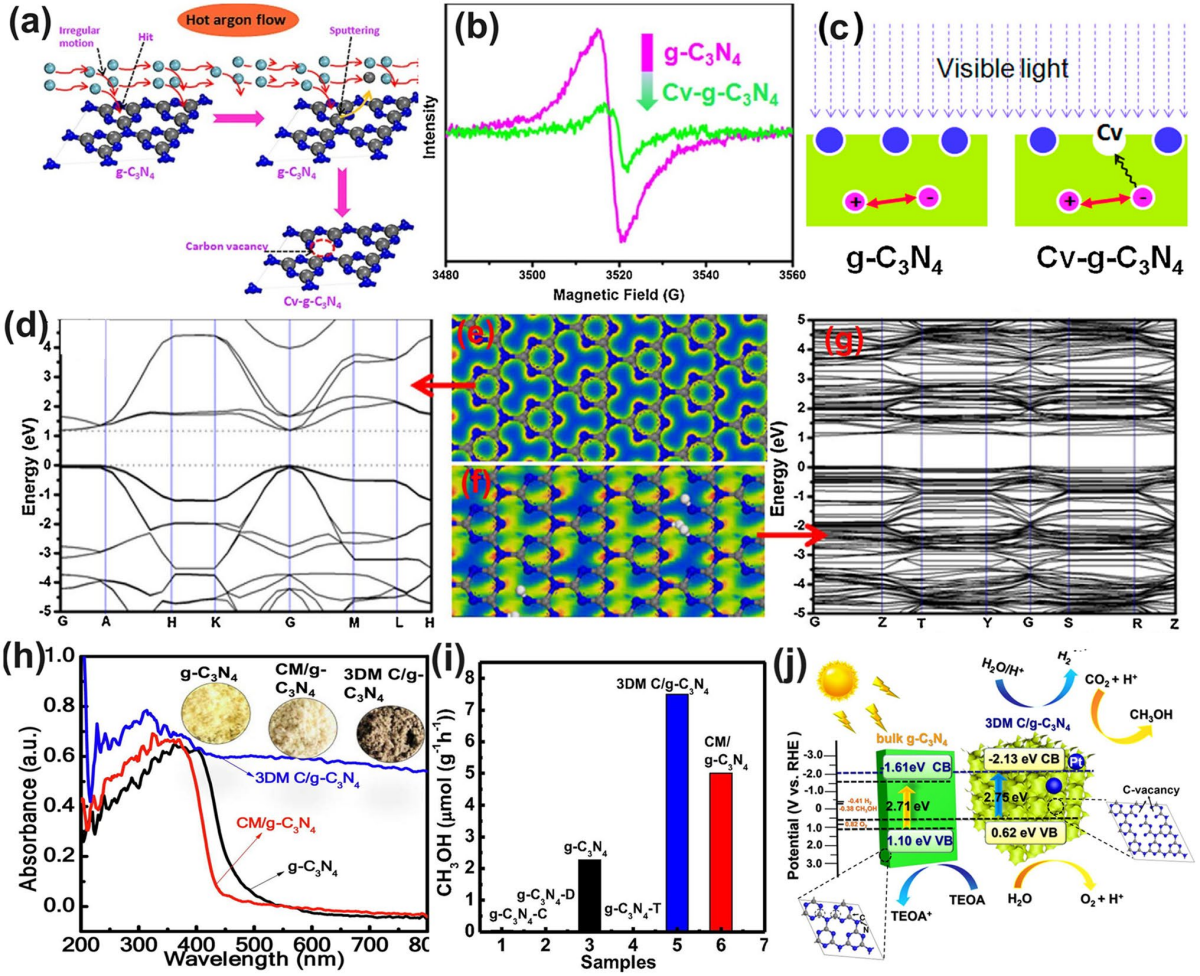
Defect control of C_3N_ vacancies. **a** Possible formation mechanism of carbon vacancy; **b** ESR spectra of g-C_3_N_4_ and Cv-g-C_3_N_4_; **c** C vacancy could significantly inhibit the recombination of photogenerated carriers; **d** Calculated band structure of g-C_3_N_4_; **e** calculated electron density of g-C_3_N_4_; **f** Calculated electron density of Cv-g-C_3_N_4_; **g** Calculated band structure of g-C_3_N_4_ [100]. Copyright 2016, Elsevier. **h** UV–vis DRS and optical photographs; **i** CH_3_OH yields of g-C_3_N_4_, g-C_3_N_4_-C, g-C_3_N_4_-D, g-C_3_N_4_-T, CM/g-C_3_N_4_, and 3DM C/g-C_3_N_4_, where C, D, T, and CM present the precursors of cyanamide, dicyandiamide, thiourea, and a mixture of cyanuric acid and melamine; **j** Schematic diagram illustrating the band structures of g-C_3_N_4_, 3DM C/g-C_3_N_4_ and the probable photocatalytic process [117]. Copyright 2021, Elsevier

Giving a simple simulated model of g-C_3_N_4_ with a triazine unit, Wang’s group suggested that the vacant C only appeared in the three-coordinated sites (Fig. 5a). In short, the vacancies could be obtained after a facile annealing process of bulk g-C_3_N_4_ under hot Ar flow at 520 °C for 1 h [100]. The Ar molecules were very active with high energy doing the irregular motion and thus had a bigger chance to hit the C atom surface than the N atom due to the smaller carbon molecular weight. Consequently, the C atoms were sputtered from the triazine framework to form the C vacant g-C_3_N_4_ (Cv-g-C_3_N_4_). This was in good agreement with the smaller peak area ratio of C–N_3_/C–C for Cv-g-C_3_N_4_ (1.2) than bulk g-C_3_N_4_ (2.7). Therefore, it was reasonable to see the weaker EPR signal of a Lorentzian line centered at about 3512 G, suggesting the decreased unpaired electron density around C vacancies (Fig. 5b). Interestingly, the authors claimed that these C vacancies could induce unsaturated N atoms with paramagnetic centers to attract more electrons from CB and break the symmetry of Cv-g-C_3_N_4_ with electron delocalization, further suppressing the photocarrier recombination (Fig. 5c). As for the calculated electronic band structures, Cv-g-C_3_N_4_ displayed a narrower bandgap and higher energy level density of VB than pristine g-C_3_N_4_ (Fig. 5d-g), revealing its enhanced solar light harvesting ability and more excitable electrons due to the electron delocalized effect caused by C vacancies. Benefiting from the above-mentioned factors, Cv-g-C_3_N_4_ changed the H_2_O_2_ formation pathway from a two-step single electron indirect reduction into a one-step two-electron direct reduction way, delivering a 14-times higher H_2_O_2_ formation than bulk g-C_3_N_4_. Delivering the same C vacant position, Wang and co-workers synthesized the 3D macropore g-C_3_N_4_ with C vacancy (3DM C/g-C_3_N_4_) via the calcination of polymethylmethacrylate (PMMA) spheres with cyanuric acid and melamine at 500 °C for 2 h [117]. The authors claimed that 3DM C/g-C_3_N_4_ had abundant macropores due to the in situ thermal removal of PMMA spheres, arousing an increased BET surface area with plentiful reactive sites for better capture and utilization of visible light. According to the UV–visible diffuse reflectance spectra (DRS, Fig. 5h), due to the introduction of C vacancies, the 3DM C/g-C_3_N_4_ showed a much wider solar light absorption range from 400 to 800 nm, which was significantly stronger than its counterparts of g-C_3_N_4_ and CM/g-C_3_N_4_ with the corresponding limited absorption edges of 460 and 435 nm, respectively. Furthermore, the photoluminescence (PL) spectra illustrated a much lower 3DM C/g-C_3_N_4_ intensity compared to those of CM/g-C_3_N_4_ and original g-C_3_N_4._ This implied the presence of C vacancies in 3DM C/g-C_3_N_4_ can not only enhance light absorption but also suppress photocarrier recombination, which finally boosted the highest CH_3_OH formation rate up to 7.5 μmol g^−1^ h^−1^ (Fig. 5i). Most importantly, the electronic band structure showed that, due to C vacancies, the 3DM C/g-C_3_N_4_ displayed a more negative CB position than bulk g-C_3_N_4_ by 0.52 eV, further indicating its stronger driving force toward CO_2_ reduction (Fig. 5j).

#### N Vacancies with Defect States

**Fig. 6:**
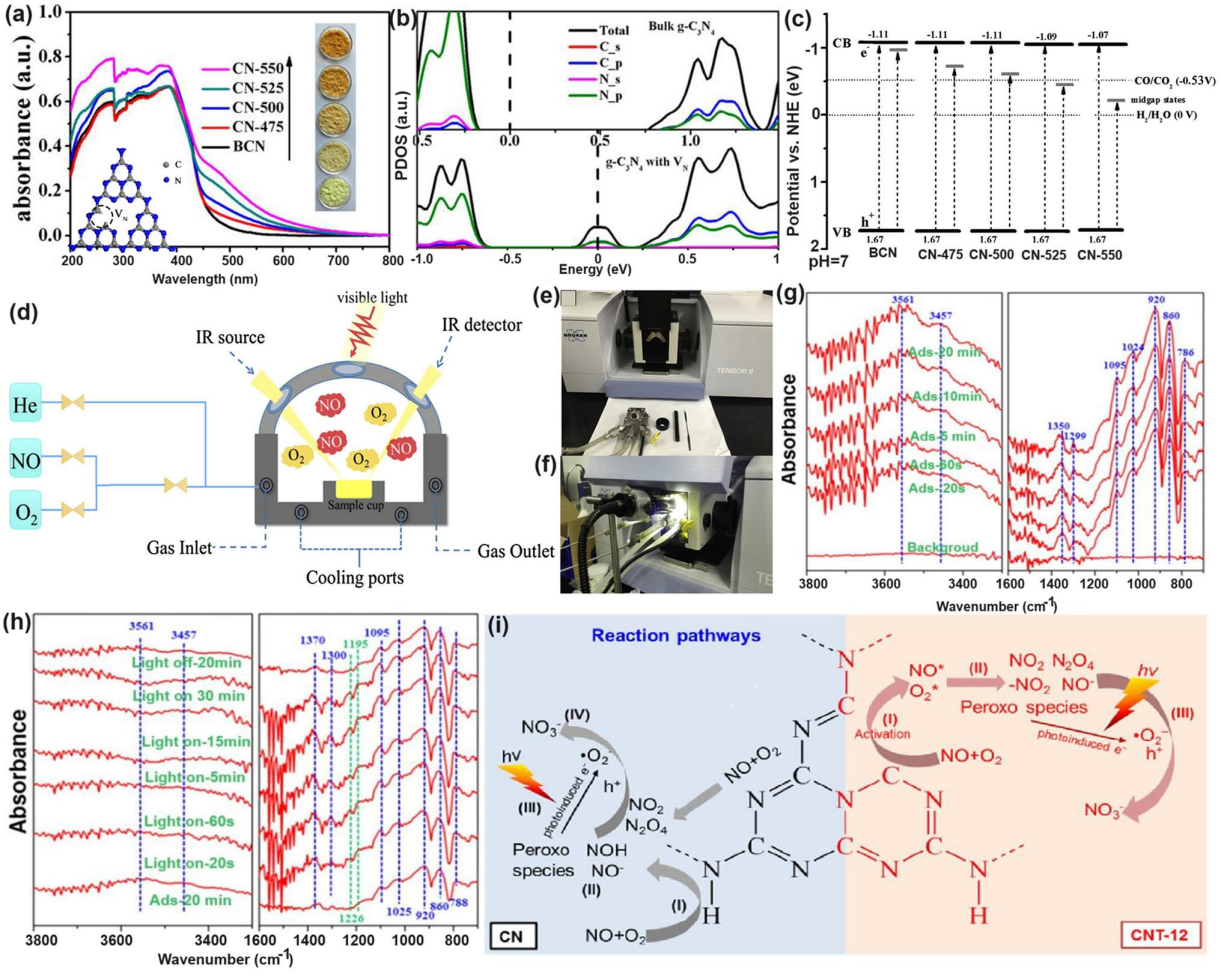
Defect control of N_2C_ vacancies. **a** UV–vis DRS of BCN and CN-*x*; **b** calculated PDOS of BCN and N vacant g-C_3_N_4_; **c** schematic illustration of the electronic structure of BCN and CN-*x* [101]. Copyright 2017, American Chemical Society. **d** Schematic diagram of in situ FT-IR reaction cell; digital photos of **e** a Tensor II FT-IR spectrometer and loading parts and **f** in situ FT-IR measurement working condition [113]. Copyright 2018, Elsevier. In situ DRIFTS images for **g** adsorption of NO/O_2_ and **h** photocatalytic reactions of Nv-CN; **i** proposed reaction pathways for adsorption and the photocatalytic oxidation of NO over pristine (left) and N-deficient (right) g-C_3_N_4_ [39]. Copyright 2019, American Chemical Society

Similar to the O vacancy-induced Ti^3+^ in the TiO_2_ system with additional defect states [118], Niu et al. claimed that the N vacancies in g-C_3_N_4_ could also arouse C^3+^ states with new energy levels [114], which has also been demonstrated by the following work on N_2C_ vacant g-C_3_N_4_ [101]. In detail, the target defective samples were prepared by the annealing of bulk g-C_3_N_4_ (BCN) from 475 to 550 °C under an H_2_ atmosphere, labeling as CN−*x* (*x* was heating temperature). Their N_2C_ vacant position was confirmed by the decreasing XPS peak area ratio of C–N=C/N–C_3_ at 3.82 and the increasing EPR signal of CN−*x* in comparison with those of BCN. It is interesting to see, with the increasing heating temperature, the color of CN−*x* gradually turned to brown, and the Urbach tail became wider with enhanced solar harvesting ability (Fig. 6a). This was ascribed to the defect states (also called midgap states), which had also been verified by the additional energy levels around Femi levels near CBM of defective g-C_3_N_4_ via DFT calculations results (Fig. 6b). It is worth to mention that the N vacancy induced defect states were deeper as the increased heating temperature (Fig. 6c), which was good for solar harvesting but detrimental for photocarrier separation. As these deep defect states could act as photocarrier recombination centers to lower the photoactivity of defective g-C_3_N_4_. As a result, we observed CN-550 exhibited an inferior photocatalytic H_2_ evolution rate of 55.64 μmol h^−1^, which was 13.5% lower than CN-525. This point is of critical importance for future defect design.

The N vacancies have been demonstrated to be efficient in boosting the g-C_3_N_4_-based NO removal. For instance, Dong et al. synthesized the N vacant g-C_3_N_4_ via the heat treatment of urea (CN-U) with a considerably enhanced EPR signal [113]. Impressively, they employed an in situ FT-IR setup to monitor the active species change and reveal the redox mechanism. As reflected in Fig. 6d–f, the instrument was composed of an FT-IR spectrometer, a diffuse reflectance cell with IR and solar irradiation windows, a high-temperature reaction chamber, a gas line, and a cooling system. The gas inlet and outlet enabled the chamber purification to obtain the clean NO and O_2_ feeding gas. The diffusion testing mode could identify the real-time active species by identifying the typical functional groups of NO oxidization intermediates. As a result, CN-U was observed with much stronger NO absorption and activation performance during the redox reaction. In a following-up work, the in situ DRIFTS observation and in-deep calculations were carried out to the inner mechanism by employing the prepared N_2C_ vacant g-C_3_N_4_ (Nv-CN) as photocatalyst [39]. Taking the best Nv-CN sample for example, its in situ DRIFTS confirmed that the new peaks around 1350/1299 cm^−1^ and 1024 cm^−1^ were nitro compounds (-NO_2_) and bidentate-state, respectively (Fig. 6g). Other peak intensities around 3604, 1095, and 786 cm^−1^ were much higher than pristine g-C_3_N_4_, further demonstrating the efficient absorption and activation of NO on the Nv-CN surface. However, its NOH peak intensity was much weaker, suggesting NO and O_2_ were rapidly absorbed on Nv-CN, rather than generating the less active terminal N–H bonds. Furthermore, once irradiation, Nv-CN also showed new peaks at around 1500–1600, 1226, and 1192 cm^−1^, assigning to the monodentate nitrate bidentate nitrate, and bidentate nitrite, respectively (Fig. 6h) [119]. Additionally, peaks assigned to other types of nitrates and peroxo species of Nv-CN were much stronger than those of unmodified g-C_3_N_4_, implying the boosted photocatalytic activity owing to the presence of N_2C_ vacancies. Based on the above DRIFTS analysis, one can conclude that the different reaction pathways (Fig. 6i): (1) For pristine g-C_3_N_4_, it showed a poorer absorption and activation ability of NO and O_2_, delivering a primary and less-active pathway of ${\text{NH}}\to {\text{NOH}}\to {\text{OOH}}\to {{\text{NO}}}^{-}$ and surface peroxo species. (2) For Nv-CN, it showed a more efficient redox pathway of directly generating bidentate states into –NO_2_, then to NO_3_^−^ in the presence of ·O_2_^−^. This promoted NO removal activity of Nv-CN was ascribed to its significantly enhanced adsorption energy toward O_2_ (− 5.99 eV) and NO (− 5.91 eV) with spontaneously bond breaking than pristine g-C_3_N_4_ (0.48 and 0.29 eV) according to DFT calculations. Additionally, the authors also claimed the N vacancy concentration was critical to boost the best photocatalytic NO removal efficiency. Otherwise, these would become photocarrier traps, leading to severe photocarrier recombination. This meaningful work has paved researchers with new insight to redox mechanism along with more precise controls are needed for future vacancy study.

Taking the best Nv-CN sample for example, its in situ DRIFTS confirmed that the new peaks around 1350/1299 cm^−1^ and 1024 cm^−1^ were nitro compounds (− NO_2_) and bidentate-state, respectively (Fig. 6g). Other peak intensities around 3604, 1095, and 786 cm^−1^ were much higher than pristine g-C_3_N_4_, further demonstrating the efficient absorption and activation of NO on the Nv-CN surface. However, its NOH peak intensity was much weaker, suggesting NO and O_2_ were rapidly absorbed on Nv-CN, rather than generating the less active terminal N–H bonds. Furthermore, once irradiation, Nv-CN also showed new peaks at around 1500–1600, 1226, and 1192 cm^−1^, assigning to the monodentate nitrate bidentate nitrate, and bidentate nitrite, respectively (Fig. 6h) [119]. Additionally, peaks assigned to other types of nitrates and peroxo species of Nv-CN were much stronger than those of unmodified g-C_3_N_4_, implying the boosted photocatalytic activity owing to the presence of N_2C_ vacancies. Based on the above DRIFTS analysis, one can conclude that the different reaction pathways (Fig. 6i): (1) For pristine g-C_3_N_4_, it showed a poorer absorption and activation ability of NO and O_2_, delivering a primary and less-active pathway of ${\text{NH}}\to {\text{NOH}}\to {\text{OOH}}\to {{\text{NO}}}^{-}$ and surface peroxo species. (2) For Nv-CN, it showed a more efficient redox pathway of directly generating bidentate states into -NO_2_, then to NO_3_^−^ in the presence of ·O_2_^−^.

**Fig. 7:**
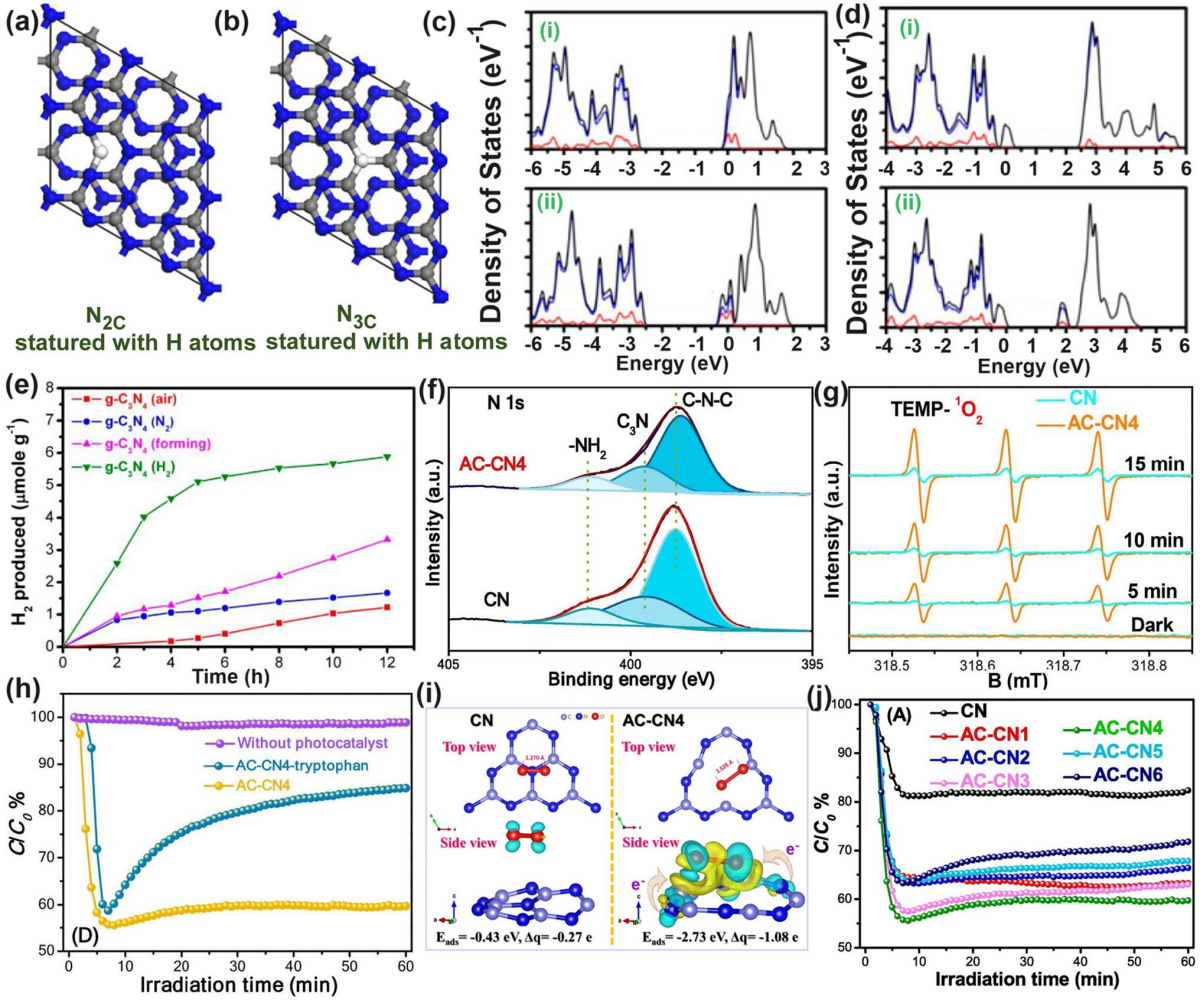
Defect control of N_2C_ and N_3C’_ vacancies. Calculated models of g-C_3_N_4_ with **a** N_2C_ statured with H atoms and **b** N_3C_ statured with H atoms; **c** PDOS of (i) N_3C_ and (ii) three-coordinated H substitution at N_3C_ site; **d** PDOS of (i) N_2C_ and (ii) two-coordinated H substitution at N_2C_ site. Color code: red (s orbital), blue (p orbital), and black (total); **e** Photocatalytic HER comparison of various g-C_3_N_4_ samples [120]. Copyright 2019, American Chemical Society.** f** N 1* s* spectra; **g** TEMP (2,2,6,6-tetramethylpiperidinooxy) spin-trapping EPR spectra for^1^O_2_ of CN and AC-CN4 in dark and under visible-light (λ ≥ 420 nm); **h** NO oxidation comparison of AC-CN4 with the addition tryptophan scavenger; **i** adsorption energy and charge density difference of CN and AC-CN4 with NO; **j** photocatalytic NO oxidation curves over different photocatalysts [121]. Copyright 2020, Elsevier

Chen’s group has compared the N_2C_ and N_3C_ vacancies in affecting the electronic band structures of g-C_3_N_4_ by DFT calculations using the simple triazine-based framework as calculated models [120]. As the unstable nature of N vacancies, they also employed the H atoms to statured with these N defects in theoretical analysis and used the H_2_ atmosphere to get the N vacant g-C_3_N_4_ with amino group in experiment (Fig. 7a). Regarding the pure N_3C_ vacant g-C_3_N_4_, it showed larger bandgap values than the N_2C_ one, indicating its inferior role in enhancing the solar light harvesting ability (Fig. 7b). So as the H statured N-deficient g-C_3_N_4_. However, we observed the latter had a much lower bandgap than the former, which was attributed to the band-like defect states below the CBM. This explained why the above-mentioned CN−*x* had new defect states under the same H_2_ atmosphere (Figs. 7c–e and 6c). As a result, the experimental N-vacant g-C_3_N_4_ with edge H atoms prepared under H_2_ (g-C_3_N_4_ (H_2_)) exhibited the highest HER rate, which was 4.8 times higher than pristine g-C_3_N_4_. Recently, Li et al*.* prepared the N_3C_ vacant AC-CNx through the calcination of melamine/azodicarbonamide (AC), where x is the mass of AC while the melamine mass was kept at 10 g [121]. Compared to pristine g-C_3_N_4_, AC-CN4 showed a reduced XPS intensity of –C_2_N and –C_3_N, and increased –N_2_C/–N_3_C peak-area ratio by 0.867 than those of bulk g-C_3_N_4_ (CN), further indicating the N vacancies were located at –N_3_C sites (Fig. 7f). Interestingly, during the NO removal activity, these N vacancies played a critical role in boosting the concentration of singlet O_2_ (^1^O_2_), which was verified to be the active species as reflected by the TEMP spin trapping EPR spectra (Fig. 7g) and active species confirmation experiment (Fig. 7h). According to the theoretical calculations, in contrast with CN, AC-CN4 exhibited an enhanced NO and ^1^O_2_ adsorption energy by 1.12 and 2.3 eV, respectively (Fig. 7i). This was ascribed to the strong electronic polarization effect, which contained electron-rich and electron-poor areas, giving a polar chemical interaction with other gas. Thus, the redox kinetics were accelerated. In addition, the incorporated N vacancies as the reactive sites in AC-CN4 also quenched the adsorption of intermediates (NO_2_)/final products (NO_2_^–^ and NO_3_^–^). All these factors rendered AC-CN4 with a significantly improved NO removal rate of 40.3%, which was 2.28-fold higher than CN, reflecting the efficient role of N vacancies (Fig. 7j).

**Fig. 8:**
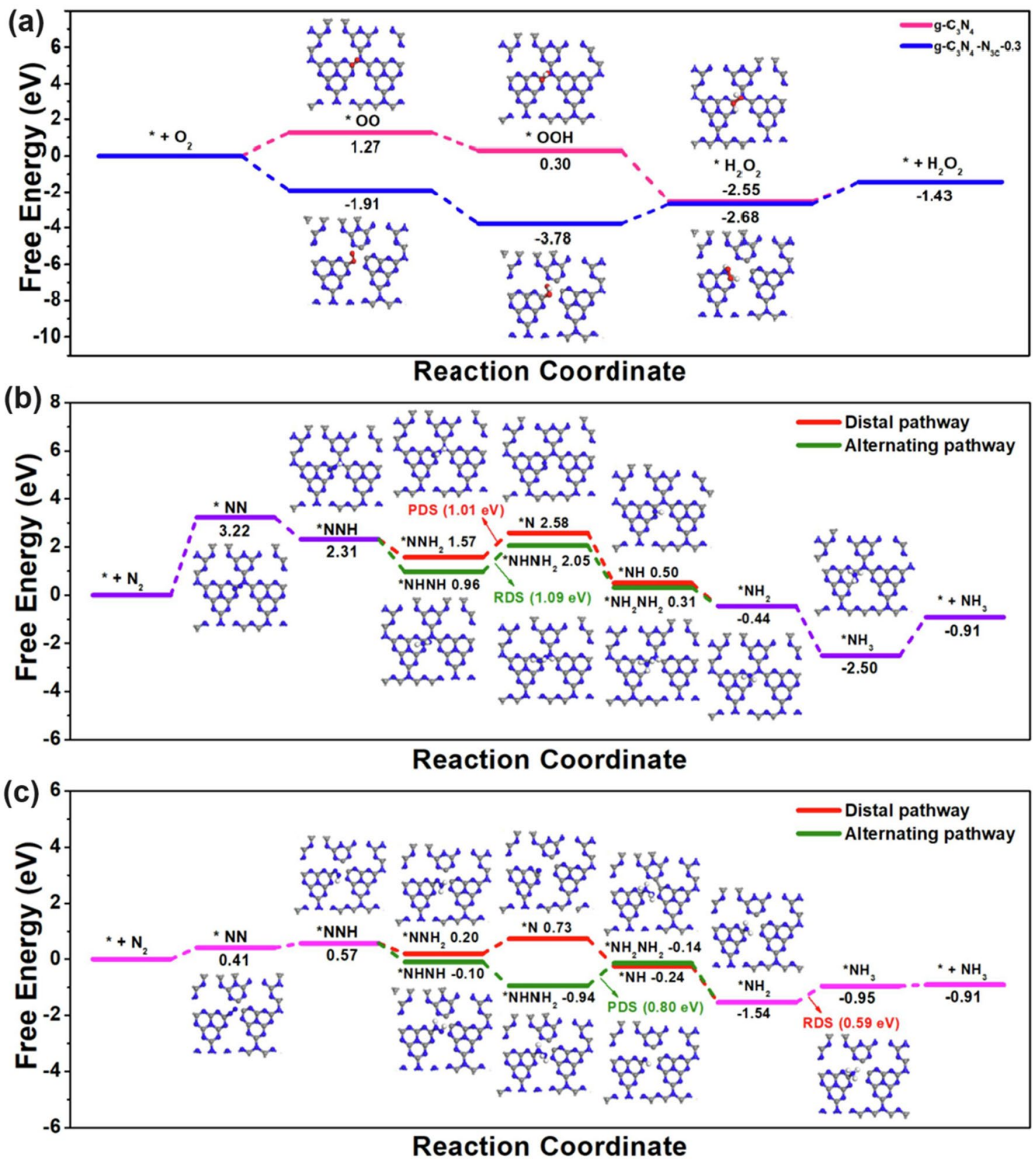
Defect control of N_3C_ vacancies. **a** Gibbs free energy diagrams for photocatalytic H_2_O_2_ production of g-C_3_N_4_ and g-C_3_N_4_ with N_3C_ vacancies; Gibbs free energy diagrams of the distal and alternating pathway of N_2_ reduction process of **b** g-C_3_N_4_ and **c** g-C_3_N_4_ with N_3C_ vacancies [122]. Copyright 2023, Elsevier

Similarly, Tian and co-workers synthesized the N_3C_ vacant g-C_3_N_4_ via the polymerization of urea (10 g) and ammonium acetate (0.1–0.5 g), of which the product was named g-C_3_N_4_-N_3C_-*X* (*X*: mass of ammonium acetate) [122]. It is worth mentioning that the decomposition of ammonium acetate would generate CO_2_ and NH_3_, which was critical to etch the N_3C_ lattices, leaving the g-C_3_N_4_ with gas bubbles during the pyrolysis process, and thus produced a porous nanosheets structure. In comparison with pure g-C_3_N_4_, g-C_3_N_4_-N_3C_-*X* displayed a reduced peak area ratio of N_3C_/N_2C_ from 0.42 to 0.31, further evidencing the formation of N_3C_ vacancies. Remarkably, when the ammonium acetate mass reached 0.3 g, the N vacant g-C_3_N_4_ achieved the highest H_2_O_2_ and N_2_ fix rates of 1098 and 1086 μmol g^−1^ h^−1^, which was 11.1 and 15.5 times higher than pure g-C_3_N_4_. The authors further employed DFT calculations to reveal the reaction mechanism of H_2_O_2_ and N_2_ reduction, respectively. As shown in Fig. 8a, we observed a much smoother reaction pathway of O_2_ reduction on g–C_3_N_4_–N_3C_-0.3 with the highest Gibbs free energy change (Δ*G*) of 1.1 eV from *OOH to *H_2_O_2_ step, which was 0.17 eV smaller than that of pure g-C_3_N_4_ to form *OO specie. Regarding to the NRR reaction, the situation was much more complex as the reaction mechanisms can be classified into the distal pathway and alternating pathway. In detail, for the former pathway, one can see the rate-determining step of pure g-C_3_N_4_ was between *NNH_2_ to *N with a Δ*G* of 1.01 eV, which was 0.42 eV higher than that of g-C_3_N_4_-N_3C_-0.3 for the conversion from *NH_2_ to *NH_3_ (Fig. 8b–c). For the latter pathway, a similar result was also reflected by the 0.29 eV lower Δ*G* for g-C_3_N_4_-N_3C_-0.3. Additionally, O_2_ preferred to be spontaneously absorbed onto the g-C_3_N_4_-N_3C_-0.3 surface, and N_2_ exhibited a dramatically reduced absorption and active barrier on its surface than pure g-C_3_N_4_ due to the much lower or even negative Δ*G* up to − 1.91 eV, strongly suggesting the ultra-active sites of the N_3C_ vacancy.

**Fig. 9:**
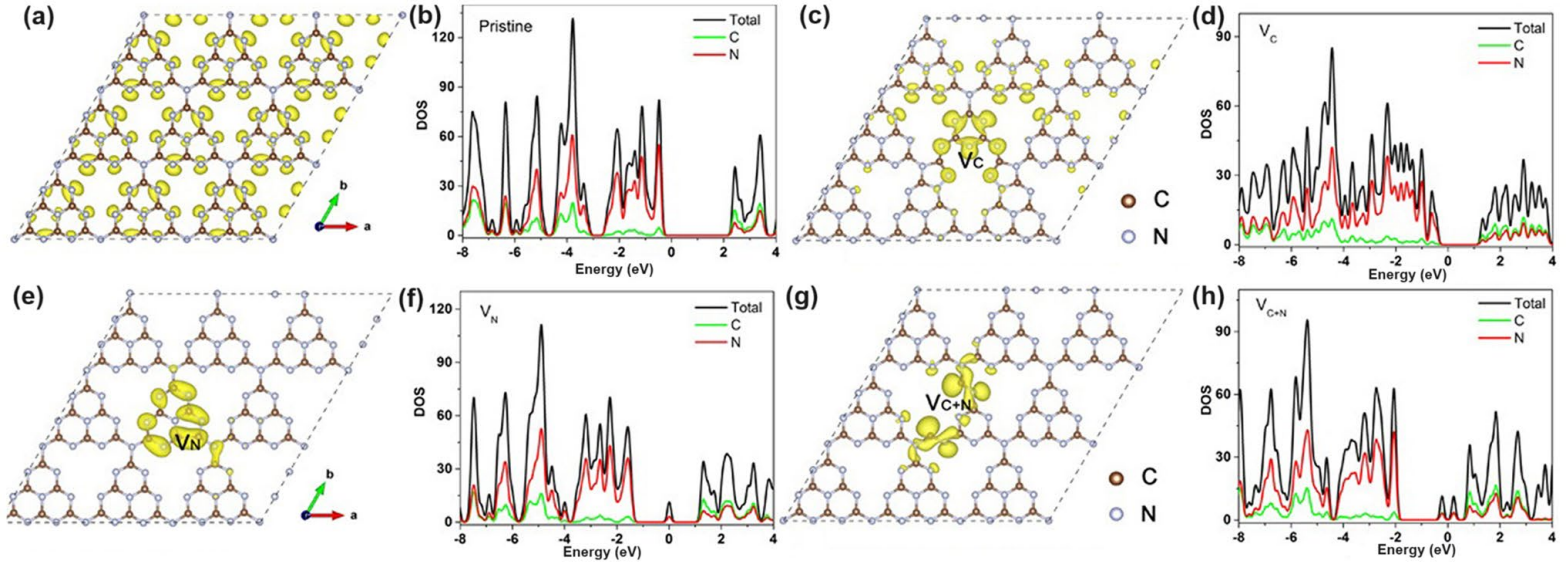
Defect control of C_3N’_ and N_2C_ vacancies. Optimized atomic structures and DOSs diagrams of **a, b** pure g-C_3_N_4_; **c, d** C vacant g-C_3_N_4_; **e, f** N vacant g-C_3_N_4_; **g, h** C vacant and N vacant g-C_3_N_4_. The Fermi levels are located at 0 eV [123]. Copyright 2019, Wiley–VCH

Apart from the single C vacancies and single N vacancies in the g-C_3_N_4_ matrix, researchers started to explore the synergistic effect of both vacancies on photocatalytic performance. A typical synthesis and theoretical work were carried out by Ren’s group [123]. They used a very ingenious He^+^ ion irradiation method to avoid the impurities from extra chemicals. The irradiation ions with a certain energy E_0_ would hit the atoms and gradually lose energy after a series of collisions with target atoms. If the hitting energy was larger than the displacement energy, the C/N atoms would be sputtered out. In this case, the input energy was high enough to hit both C and N atoms out, and this non-chemical selectivity made both vacancies exist simultaneously. By controlling the hitting parameters with total energy fluence from 0 to 86.25 × 10^13^ ions cm^−2^, the C/N vacancies gradually increased, of which the N defect concentration was much higher than C according to the experimental XPS analysis and theoretical Stopping and Range of Ions in Matter (SRIM) simulations. Despite the experimental failure to obtain single C or single N vacant g-C_3_N_4_, their DFT calculations explained the influence of single vacancy and C/N vacancies on the electronic band structures (Fig. 9). Compared to the bulk g-C_3_N_4_, one can see that the C vacant g-C_3_N_4_ had a dramatically reduced bandgap by 0.97 eV, only 1.48 eV, extending the optical absorption (Fig. 9a–d). For the V vacant g-C_3_N_4_ case, its bandgap was slightly reduced by 0.07 eV and formed the C–C bond into a five-ring unit to keep the structure stable. Interestingly, defect states were lying below the CBM of V vacant g-C_3_N_4_ (Fig. 9e), which was also consistent with the obvious tail absorption in the DRS result. The calculated electronic density results indicated the electrons preferred to localize around the N vacancies (Fig. 9f–h). Notably, the presence of both C and N vacancies enabled g-C_3_N_4_ with slight bandgap narrowing and more defect states localized around the vacancies. The authors also claimed that this was good for electron-trapping to enhance the photocarrier separation. In summary, the C vacancy was more effective in narrowing the bandgap while the N vacancy was more useful in creating defect states. Taking together, we can observe both optimized solar harvesting ability and photocarrier transfer. As a result, the C/N vacancy co-modified g-C_3_N_4_ exhibited a significant HER rate of 1271 μmol g^−1^ h^−1^, which was 19 times higher than the bulk g-C_3_N_4_.

Therefore, based on the above review, we can come to a summary of this vacancy section. The C vacancies and N vacancies realized by annealing bulk g-C_3_N_4_ or modified precursors under different atmospheres or physical treatments such as plasma environments are both beneficial to optimize the electronic band structure with enhanced solar light absorption and photocarrier transport. Additionally, recent reports on defective g-C_3_N_4_ with vacancies at different positions toward various solar applications are listed and comparable in Table 1. We believed this would help readers to find clues for more precise control of vacancy creation. Despite significant progress has been made, there also remain some ambiguities that need to be resolved in the near future. Firstly, a single vacancy and both vacancies cannot be obtained with precise concentration control via one synthetic strategy. Their deep relationships are hard to distinguish, such as to which extent can the synergistic effect reach the optimized state. Secondly, the study on vacancy-associated defect states (midgap states) is still in the beginning, and defect control on tuning their position needs to be specified. Thirdly, the photocarrier transfer dynamics in the bulk-phase and surface are different, which urgently needs to be discussed in depth. Since the vacancies might exist in both bulk and surface, a more advanced time-resolved spectrum should be paid into this section to reveal the detailed photocarrier separation and transfer progress.

**Table 1:** Comparison of vacant g-C_3_N_4_ toward various solar applications

Photocatalyst	Defect type	Light source	Solar applications	Photocatalytic activity	Refs.
N-deficient g-C_3_N_4_	N_2C_	*λ* > 400 nm	HER	455 μmol g^−1^ h^−1^	[114]
g-C_3_N_4_	N_2C_	*λ* > 420 nm	HER	5.7 μmol g^−1^ h^−1^	[120]
RCNO	N_2C_	simulated sunlight	AO7 removal	0.01 min^−1^ g^−1^	[124]
g-C_3_N_4_	N_2C_	*λ* > 420 nm	HER	2015.5 μmol g^−1^ h^−1^	[125]
DCN-15A	N_3C_	*λ* > 420 nm	H_2_O_2_ formation	96.8 μmol g^−1^ h^−1^	[84]
**20KCSCN**	N_2C_	*λ* > 420 nm	HER	652 mmol g^−1^ h^−1^	[126]
ECNV-2.5	N_2C_	*λ* > 420 nm	acetone formation	467.6 ppm min^−1^ g^−1^	[127]
gcnse-1	N_2C_	*λ* > 420 nm	HER	1.16 mmol g^−1^ h^−1^	[128]
**CNCN**	N_3C_	*λ* > 420 nm	HER	3591 μmol g^−1^ h^−1^	[129]
g-C_3_N_4_	N_2C_	*λ* > 420 nm	HER	1194.8 mmol g^−1^ h^−1^	[130]
ODN-CN2	N_2C_	*λ* > 420 nm	HER	5833.1 μmol g^−1^ h^−1^	[131]
ZCNQ50	N_2C_	simulated sunlight	HER	4368 μmol g^−1^ h^−1^	[132]
**SA-Cu-CN + Nv**	N_2C_	*λ* > 420 nm	HER	605.15 μmol g^−1^ h^−1^	[133]
NV-g-C_3_N_4_	N_2C_	Visible light	HER	3259.1 μmol g^−1^ h^−1^	[134]
**VCN**	N_2C_	*λ* > 420 nm	Pollutant removal (NO_2_)	0.0183 min^−1^ g^−1^	[135]
CN-M-630	N_2C_	*λ* > 420 nm	HER	5304.3 μmol g^−1^ h^−1^	[136]
Nv-CNN-3	N_2C_	*λ* > 420 nm	H_2_O_2_ formation	1768 μmol g^−1^ h^−1^	[137]
**PHCN3**	N_3C_	*λ* > 420 nm	HER	3631 μmol g^−1^ h^−1^	[138]
FNGK-10	N_2C_	*λ* > 420 nm	BPA removal	0.327 min^−1^ g^−1^	[139]
C_3_N_4_-Cl_4_	N_2C_	*λ* > 300 nm	Water spitting	9640 μmol g^−1^ h^−1^	[140]
HCN-C_0.5_	N (n/a)	*λ* > 400 nm	MPB removal	1.1 min^−1^ g^−1^	[141]
g-C_3_N_4_ − x/g- C_3_N_4_	N_2C_	Visible Light	Atrazine removal	0.239 min^−1^ g^−1^	[117]
NFS/NCN-2	N (n/a)	Visible Light	Cr^4+^ removal	1.272 min^−1^ g^−1^	[142]
CN-CV	C_3N_	Visible light	H_2_O_2_ formation	8143.5 μmol g^−1^ h^−1^	[143]
g-C_3_N_4_-V	C_3N’_	simulated sunlight	NRR	84 μmol g^−1^ h^−1^	[144]
V_C_-g-C_3_N_4_	C_3N’_	Visible light	HER	450 mmol g^−1^ h^−1^	[145]
HGCN	C_3N’_	simulated sunlight	NRR	25.54 ppm g^−1^_cat_ h^−1^	[146]
**VCN**	C_3N_	*λ* > 420 nm	HER	5.12 mmol g^−1^ h^−1^	[147]
C_1.0_CN	C_3N_	*λ* > 420 nm	BPA removal	3.37 min^−1^ g^−1^	[148]
3DM C/g-C_3_N_4_	C_3N_	simulated sunlight	HER	64.7 μmol g^−1^ h^−1^	[149]
g-C_3_N_4_	C_3N_	*λ* > 400 nm	HER	10.14 μmol g^−1^ h^−1^	[150]
VCN	C_3N_	*λ* > 420 nm	HER	3304.5 μmol g^−1^ h^−1^	[151]
PCN	C (n/a)	*λ* > 420 nm	Phenol removal	4375 μmol g^−1^ h^−1^	[152]
GCN	C_3N_	*λ* > 420 nm	H_2_O_2_ formation	507.82 mmol g^−1^ h^−1^	[153]
Ti_3_C_2_T_x_/Vc-CN	C_3N’_	*λ* > 400 nm	CRR	20.54 μmol g^−1^ h^−1^	[154]
CCN	C (n/a)	*λ* > 420 nm	Lidocaine removal	10.1 min^−1^ g^−1^	[155]
CN-DEG	C (n/a)	Visible Light	HER	540 μmol g^−1^ h^−1^	[156]
HTCN	C_3N’_	*λ* > 420 nm	HER	1534.3 μmol g^−1^ h^−1^	[157]

n/a indicates there is no discussion on vacancy sites in original publications

### Non-metal Dopants with Optimized Band Structures and Electronic Density

The metal-free merit of g-C_3_N_4_ can also be maintained by non-metal doping with heteroatoms such as C, P, S, O, B, and F [158]. Similarly, similar to C and N vacancies, these non-metal dopants also enable g-C_3_N_4_ with optimized electronic structure, enhanced visible-light harvesting, and high charge separation efficiency.

#### C Dopants with Electronic Delocalization

The C dopants, replacing the bridging N atoms in the g-C_3_N_4_ matrix, have been demonstrated to improve the bulk electronic conductivity due to the presence of delocalized big *π* bonds between the hexatomic rings and substituted C [104]. In a typical work, Zhao and co-workers fabricated the C-doped g-C_3_N_4_ using melamine and melamine-based resin foam as precursors [159]. After the thermal decomposition, C atoms were in situ doped into the g-C_3_N_4_ framework. The enhanced conductivity was verified by the reduced charge transfer resistance (*R*_ct_) according to the EIS measurement. Additionally, the C-doped g-C_3_N_4_ also shows extended solar absorption from visible light to near-infrared (800 nm). As a result, this defective C-doped g-C_3_N_4_ exhibits an excellent NO photodegradation constant of 0.95 min^−1^. A similar study was also reported by Zhang and colleagues, which employed a hydrothermal method to obtain the C-doped g-C_3_N_4_ with glucose and melamine as precursors [160].

To further boost solar light absorption and suppress photocarrier recombination, the C-rich g-C_3_N_4_ with both N vacancies and porous structure was designed [161]. Different from previously isolated C dopants, these C dopants existed in the form of C rings, which were realized by the additive of conjugated methyl-cyclodextrin. The g-C_3_N_4_ photocatalyst consisted of three layers with the pure carbon nitrides in the core, the carbon dopant layer in the middle, and the carbon layer in the outermost layer. The unique structure of gradual C-doped g-C_3_N_4_ endowed itself with not only enhanced electronic conductivity but also a narrower bandgap and stronger solar light absorption. More importantly, the C dopants and N vacancies induced the formation of mid-gap states, which could further lower the photoexcitation energy which is smaller than the bandgap. In addition, the mid-gap states can act as a temporary reservoir to accept the migrated electrons from CB, and thus the recombination process of electrons and holes was suppressed. Therefore, the C defective g-C_3_N_4_ displayed an exceptional solar-driven HER rate of 125.1 μmol h^−1^ g^−1^, which was over 21-fold as high as the pristine g-C_3_N_4_.

#### N Dopants with Defect States

**Fig. 10:**
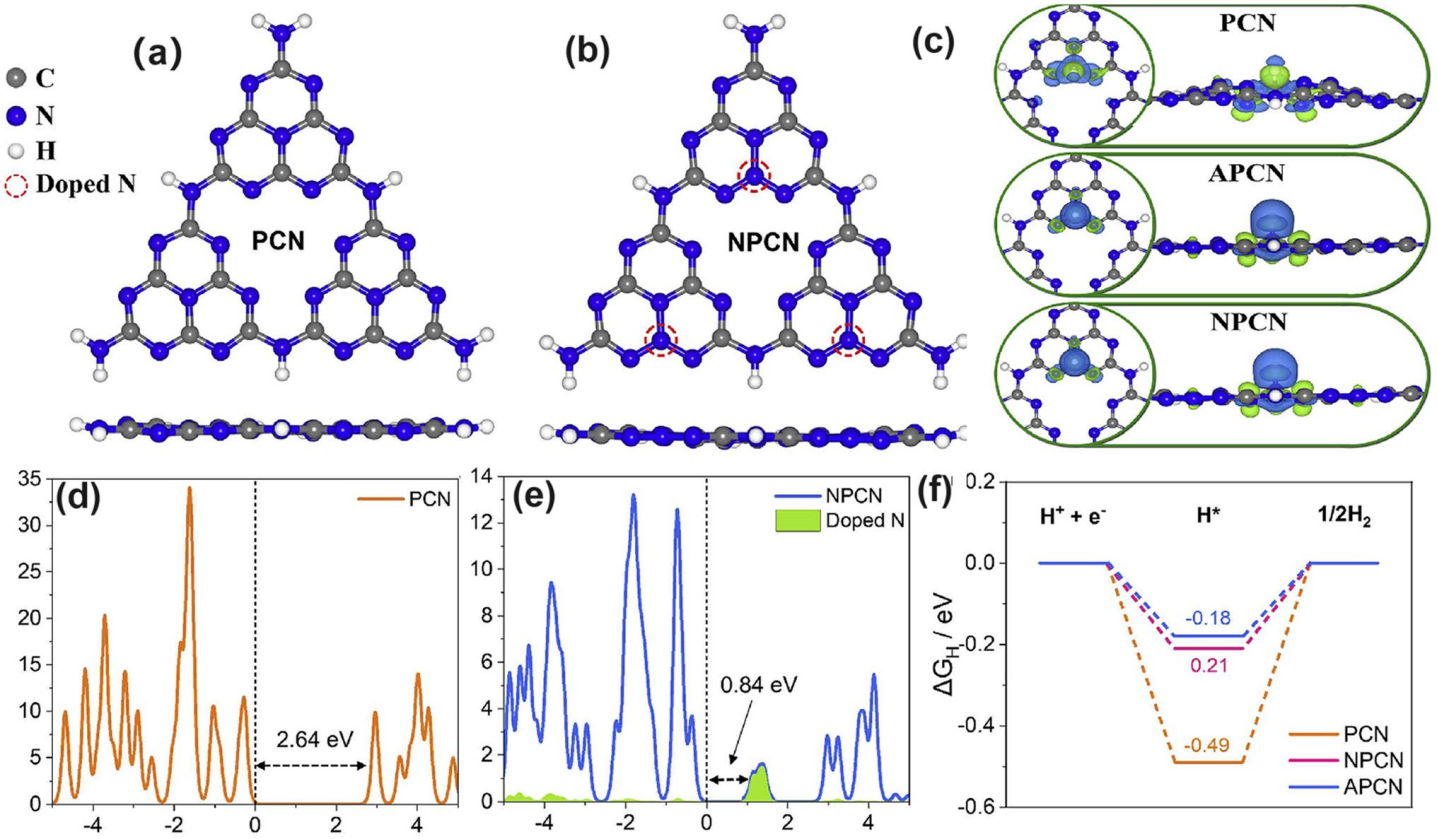
Defect control of N dopants. **a, c** Optimized electronic structure and **b, d** DOS of PCN and NPCN; **e** three-dimensional charge density distribution (blue color denotes electron accumulation, whereas green color to depletion region); **f** calculated hydrogen adsorption energy on the PCN, APCN, and NPCN catalyst [163]. Copyright 2023, Elsevier

Recently, N-doped g-C_3_N_4_ has been proposed via the annealing of melamine cyanurate supermolecules via the hydrothermal reaction of melamine and aminourea hydrochloride [162]. Doping at an edge three-coordinated C site, the doped N atoms induced defect states in the electronic band structure near CBM, thus extending the solar harvesting ability to almost 550 nm. Additionally, the N-deficient g-C_3_N_4_ exhibited an improved TC removal rate of 93.3% within 60 min. Despite the different N-doping sites of the inner three-coordinated C atom, Umare et al. explained the reason for enhanced photocatalytic HER activity of N-rich g-C_3_N_4_ in depth using the DFT calculations [163]. Before this, in the experiment, they successfully synthesized the polymerized g-C_3_N_4_ (PCN) by directly annealing melamine in air. Those prepared with lower N-doping levels employing aminoguanidine hydrochloride/urea as the precursor and higher N-doping levels with urea/aminoguanidine hydrochloride/melamine as the precursor were named APCN and NPCN. In increasing order of PCN < APCN < NPCN, we saw a gradually enhanced photocatalytic HER rate of 5.81, 6.97, and 40.32 mmol g^−1^, respectively. The authors then gave three calculated models to simulate g-C_3_N_4_ with different N-doping concentrations in terms of band structures, charge density distribution, as well as Δ*G* change (Fig. 10). Compared to PCN, NPCN displayed new energy levels that were also called defect states/midgap states in the forbidden band, mainly due to the existence of new N dopants (Fig. 10a–d). This was also true for APCN. The authors claimed that this might be more advantageous for photocarrier separation from these defect states and CBM. The electronic density pictures also revealed there were more electrons transferred from N dopants to H atoms, further accelerating the photocarrier transfer kinetics (Fig. 10e). The H_2_ evolution pathway also confirmed a smoother H* adsorption/desorption process due to the slight Δ*G* change of − 0.18 eV, which was 0.31 and 0.03 eV smaller than those of PCN and APCN, respectively (Fig. 10f). Therefore, we can conclude that N dopant is also beneficial to the photocatalytic ability optimization of g-C_3_N_4_, which was even similar to the work-principle of N vacancies as above mentioning [123].

#### P Dopants with Defect States

**Fig. 11:**
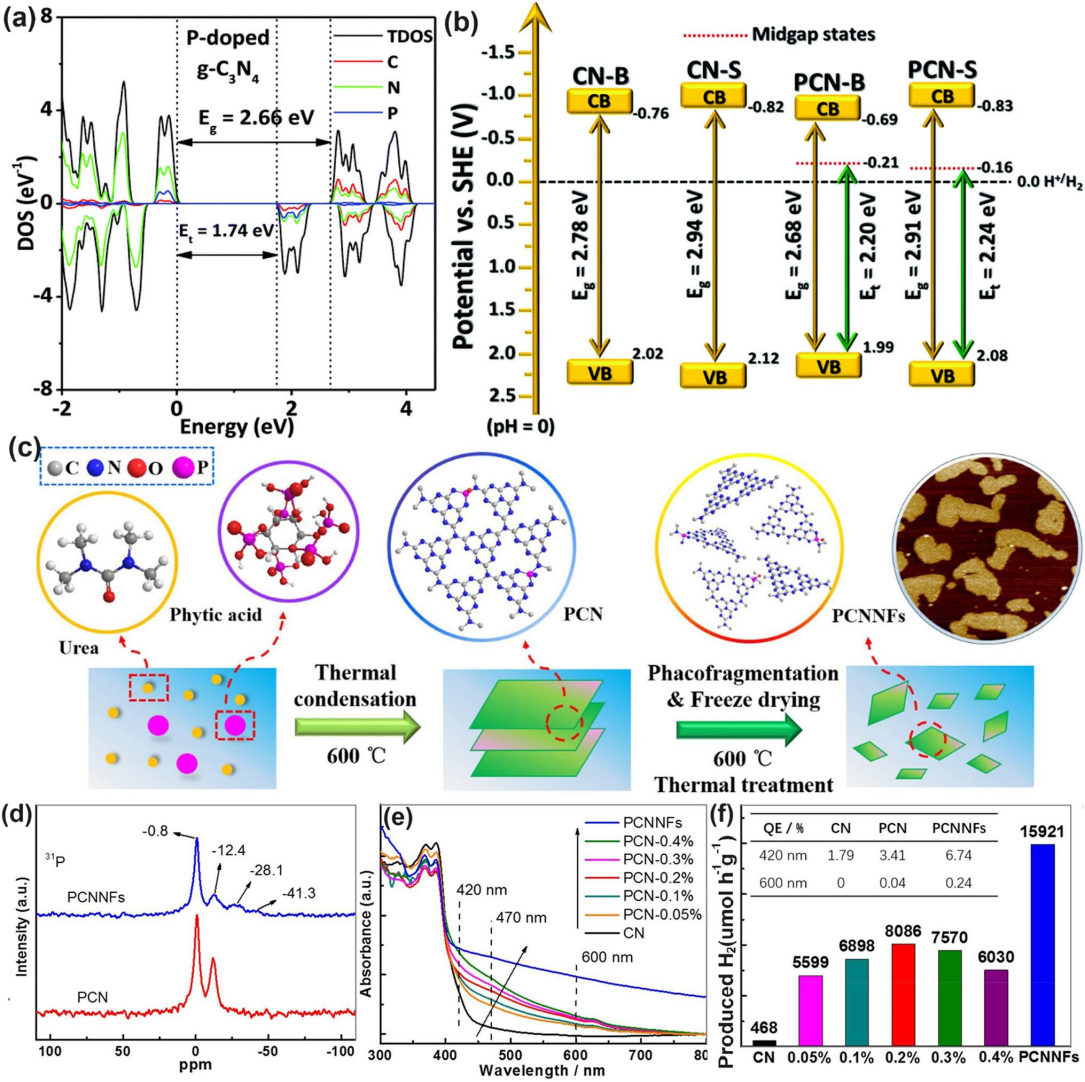
Defect control of P dopants.** a** Density of states (DOSs) of P-doped g-C_3_N_4_; **b** Electronic band structure of P-doped g-C_3_N_4_, CN-B, and CN-S [110]. Copyright 2018, Elsevier.** c** Illustration of the preparation process of PCNNFs;** d** ^31^P MAS NMR spectra of PCN and PCNNFs; **e** UV–vis DRS and **f** photocatalytic HER rates under visible-light irradiation (λ > 420 nm, inset: QE comparison at 420 and 600 nm) for CN, PCN-*x*, and PCNNFs [102]. Copyright 2015, Royal Society of Chemistry

Similar to the above-mentioned gradual C-doped g-C_3_N_4_, Ran and colleagues demonstrated that P atoms could also induce the formation of mid-gap states in g-C_3_N_4_ with an extended solar light absorption to 557 nm and high photocarrier separation efficiency both theoretically and experimentally (Fig. 11a) [110]. In addition, due to the more extensive thermal etching of protonated precursor, the porous P-doped g-C_3_N_4_ nanosheets (PCN-S) also presented a quantum size effect with an enlarged bandgap by 0.23 eV higher than the bulky P-doped g-C_3_N_4_ (PCN-B, Fig. 11b). Intriguingly, a greater reductive driving force and promoted mass-transfer process for PCN-S was achieved owing to the more negative CBM and the macroporous structure, respectively. Therefore, this PCN-S exhibited an outstanding HER rate of 1596 μmol h^−1^ g^−1^ and an apparent quantum efficiency of 3.56% at 420 nm. Researchers also found an interesting result that the phosphorous precursors played an important role in the P-doping sites [158]. For instance, the P atoms were prone to replace the bay or corner C sites in the tri-s-triazine units to form the P–N bonding when using 1-butyl-3-methylimi-dazolium hexafluorophosphate (BmimPF_6_) as P source [99]. The P atoms were found to be doped into the g-C_3_N_4_ lattices to form a P–N bond when using (NH_4_)_2_HPO_4_ as a P precursor [164]. Despite the different P doping sites, both situations can achieve excellent solar performance due to the narrowed bandgap and accelerated photocarrier transfer kinetics.

In follow-up work, Fang’s group synthesized a variety of P-doped g-C_3_N_4_ nanoflakes (PCNNFs) by a former thermal condensation of phytic acid and urea (product: PCN−*x*, *x* is the mass ratio of phytic acid/urea), followed by a nanostructure tailoring of phaco-fragmentation and freeze-drying (Fig. 11c) [102]. The porous PCNNFs exhibited fragmentized nanoflakes with significantly improved BET surface area of 223.2 m^2^ g^−1^, which shortened the interfacial diffusion path of active species and thus accelerated the transfer and separation of photocarriers. The P substituted site was proved to be the corner C connected to the tertiary amine according to the two distinctive peaks around − 0.8 and − 12.4 ppm as shown in the ^31^P solid-state magic angle spinning nuclear magnetic resonance (MAS NMR) spectra (Fig. 11d). Moreover, the excellent visible-light absorption of PCNNFs was substantially extended to 800 nm, superior to those of bulk g-C_3_N_4_ and PCN−*x*, indicating the efficiency of P-doping and advanced nanostructure (Fig. 11e). It is worth mentioning that the quantum efficiency at 600 nm of PCCNFs was about 0.24%, far more exceeding its counterparts. This was also due to the narrowed sub-bandgap from VB to midgap states induced by the P dopants. As a result, irradiated with visible light, the photocatalytic HER rate of PCNNFs was up to 15,921 μmol g^−1^ h^−1^, which was 34-folder higher than that of bulk g-C_3_N_4_ (Fig. 11f). In another typical work, Wu et al*.* used urea and butyl phosphate as precursors to prepare P-doped g-C_3_N_4_ (P_*x*_C_3_N_4_, where *x* = 1, 2, 3 corresponds to the butyl phosphate volume of 2, 5, and 8 mL) [165]. Due to the introduction of the P element, the edge of P_3_C_3_N_4_ nanosheets was smoother and more regular compared with that of bulk g-C_3_N_4_. It displayed the narrowest bandgap of 2.49 eV, which was dramatically reduced than bulk g-C_3_N_4_ (2.7 eV). Due to the P-doping, the solar absorption reached 470 nm, and thus an increased photocatalytic UO_2_^2+^ removal rate of 84% within 20 min was witnessed.

#### S Dopants with Improved Redox Driving Force

**Fig. 12:**
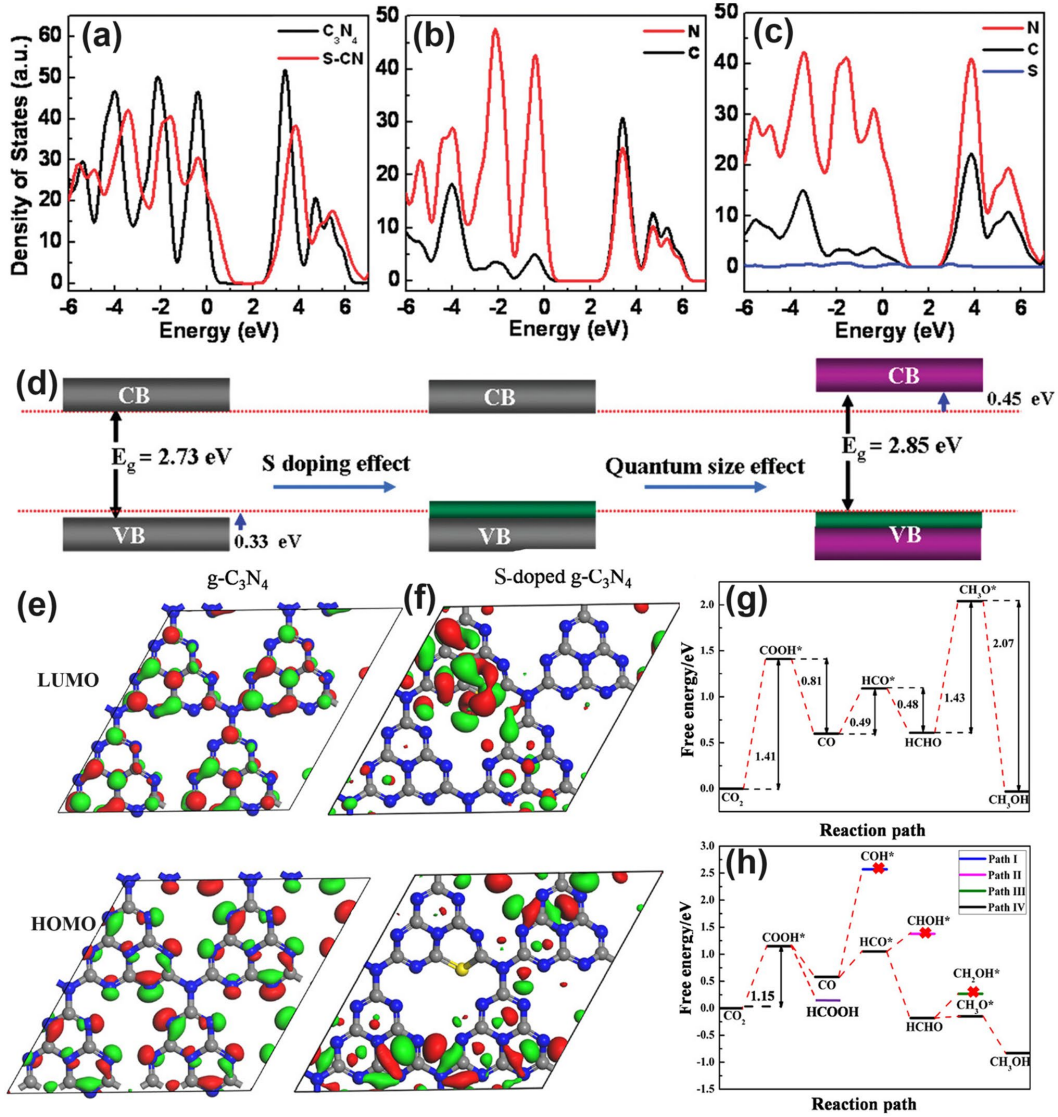
Defect control of S dopants.** a** Total DOSs of pristine C_3_N_4_ and C_3_N_4−*x*_S_*x*_; partial DOSs of **b** C_3_N_4_ and **c** C_3_N_4−*x*_S_*x*_; **d** graphic illustration of band structure change of C_3_N_4_ by S-doping and quantum confinement effect (QCE) [167]. Copyright 2010, American Chemical Society. LUMOs and HOMOs of **e** g-C_3_N_4_ and **f** S-doped g-C_3_N_4_; calculated free energy diagram to the reaction paths followed by CRR on **g** g-C_3_N_4_ and **h** S-doped g-C_3_N_4_ [55]. Copyright 2018, American Chemical Society

Early theoretical research work in 2012 revealed that the S atoms prefer to replace the two-coordination N sites in the aromatic ring and induce an impurity energy level just below the CB, which is beneficial to cause a red shift of solar light absorption threshold and improve the electronic conductivity of g-C_3_N_4_ [166]. Afterward, Chen et al*.* proposed an exceptional work on S-doped g-C_3_N_4_ employing the H_2_S atmosphere as the S feeding source to achieve a homogenous S-doping at the atomic level with the pyrolyzed product labeled as C_3_N_4−*x*_S_*x*_ [167]. Its homogenous doping was confirmed by the almost same and stable XPS signal of S 2*p* spectra during Ar^+^ sputtering from 20 to 420 s. The authors claimed that this was of vital importance to achieving: (i) the localized states induced by S dopants and (ii) the elevation of VBM through the mixing of S 3*p* states with N 2*p* states (Fig. 12a–c). The extended VB width was believed to accelerate the mobility of holes, boost charge transfer kinetics, and thus give rise to a better photo-oxidation efficacy (Fig. 12d). Furthermore, due to the unique synthetic strategy, the grain size of C_3_N_4−*x*_S_*x*_ was also dramatically reduced, inducing a remarkable quantum confinement effect (Fig. 12d). This could render it a higher driving force for redox reaction due to the more positive VBM and more negative CBM positions. Therefore, C_3_N_4−*x*_S_*x*_ showed an overwhelming phenol removal activity under irradiation with *λ* > 400 nm. Other precursors such as thiourea [168–170] and urea/benzyl disulfide [171, 172] have also been reported to work as the S source, which was much “green” than the toxic and corrosive H_2_S gas.

In another typical work, the S-doped g-C_3_N_4_ has demonstrated its superior role in boosting the photocatalytic CRR activity by altering the rate-determining step and reducing the Gibbs free energy from 1.43 to 1.15 eV [55]. Detailed theoretical calculations have been carried out using the pristine g-C_3_N_4_ and S-doped g-C_3_N_4_ molecules containing four C_6_N_7_ units as models. According to the HOMO and LUMO diagrams from Fig. 12e-f, one can see the electrons in HOMO were distributed on N atoms only. While for LUMO, electrons were localized in both C atoms and N atoms. However, no electrons appeared around the bridging N atoms, which indicated the electron in g-C_3_N_4_ would only be excited and transferred within one C_6_N_7_ unit, thus increasing the photocarrier possibility of being recombination (Fig. 12e). In contrast, the electrons in HOMO and LUMO were distributed on the undoped units and S-doped units, respectively (Fig. 12f). This implied the electrons in S-doped g-C_3_N_4_ can migrate within the surrounding C_6_N_7_ units and the photocarrier separation efficiency could be significantly enhanced. Furthermore, the accelerated thermodynamics was verified by the optimized CRR pathway. For g-C_3_N_4_, the rate-determining steps were the conversion of CO_2_ to COOH* and HCHO to CH_3_O* with Δ*G* values of 1.41 and 1.43 eV, respectively (Fig. 12g). In contrast, the determining step for S-doped g-C_3_N_4_ is the formation of COOH* only with a reduced Δ*G* of 1.15 eV (Fig. 12h), suggesting the more favorable CRR progress which was also in good accordance with previous reports [168].

Ke et al*.* employed urea and benzyl disulfide as precursors to obtain the S-doped g-C_3_N_4_ (SC_3_N_4_-*X*, *X* = 1, 2, 3 presenting the annealing temperature of 560, 600, and 650 °C) [171]. The S dopants have been found to overcome the stronger planar hydrogen bond between the tri-s-triazine unit and NH/NH_2_ group, favoring the layered g-C_3_N_4_ exfoliation into nanosheets. Thus, SC_3_N_4_-3 exhibited the highest BET surface area up to 298.2 m^2^ g^−1^, providing abundant sites for redox reactions. Consisting with previous studies, we observed that the S dopants also rendered SC_3_N_4_-3 with a reduced bandgap of 2.10 eV, which was 0.64 eV smaller than bulk g-C_3_N_4_ with an extended visible-light absorption from 458 to 530 nm. As a result, the photodegradation constant and UO_2_^2+^ removal efficiency of SC_3_N_4_-3 achieved 0.16 min^−1^ and 92%, which was 1.78 and 1.58-folder better than those of other g-C_3_N_4_ materials. Cao and co-workers further synthesized the porous S-doped g-C_3_N_4_ nanosheets with C vacancies (SCNNS_S_) by facile pyrolysis of thiourea [32]. The thickness of SCNNS_S_ was only 2.5 nm, revealing a significantly improved BET surface area of 75.24 m^2^ g^−1^. Although the presence of C vacancies broadened the bandgap of SCNNS_S_, its CB position was lifted from − 0.95 to − 1.04 eV, endowing it with a promoted driving force toward the photocatalytic NRR activity. Therefore, the nitrogen fixation rate of SCNNS_S_ reached 5.99 mM h^−1^ g_cat_^−1^, which was 2.8-fold the amount of bulk g-C_3_N_4_ (2.13 mM h^−1^ g_cat_^−1^), confirming the critical role of S doping.

#### O Dopants with Electronic Polarization

Chen and co-workers first reported that O atoms were prone to substitute the two-coordinated N atoms next to sp^2^-hybridized C atoms, forming the N–C–O and C–O bond [173]. These O dopants could significantly optimize the electronic band structure of g-C_3_N_4_ with a reduced bandgap of 0.21 eV in comparison with that of the bulk one. However, their VBM remained the same, indicating the VBM of the O-doped g-C_3_N_4_ primarily depended on the N 2*p* orbitals. Owing to the electronegativity discrepancy between N and O, more charge density would be presented near O atoms. Therefore, this would cause additional defect-related surface energy levels below the CBM, accelerating the photocarrier transfer and separation in O-doped g-C_3_N_4_. As a result, both excellent MB (methyl blue) photodegradation and H_2_ evolution rate were achieved for the defective g-C_3_N_4_. Another work presented by Zhang’s group reveals that O dopants can shorten the C–N/C = N bonds due to the more negative electronegativity of O, which shortens the charge diffusion pathway from bulk to surface and boosts the charge transfer rate [75]. Additionally, the theoretical differential charge density diagram clearly showed the electronic polarization effect aroused by O atoms, similar to the “inner-built electric field”, which gave the electron an extra transfer driving force. Thus, the HER rate was significantly improved.

**Fig. 13:**
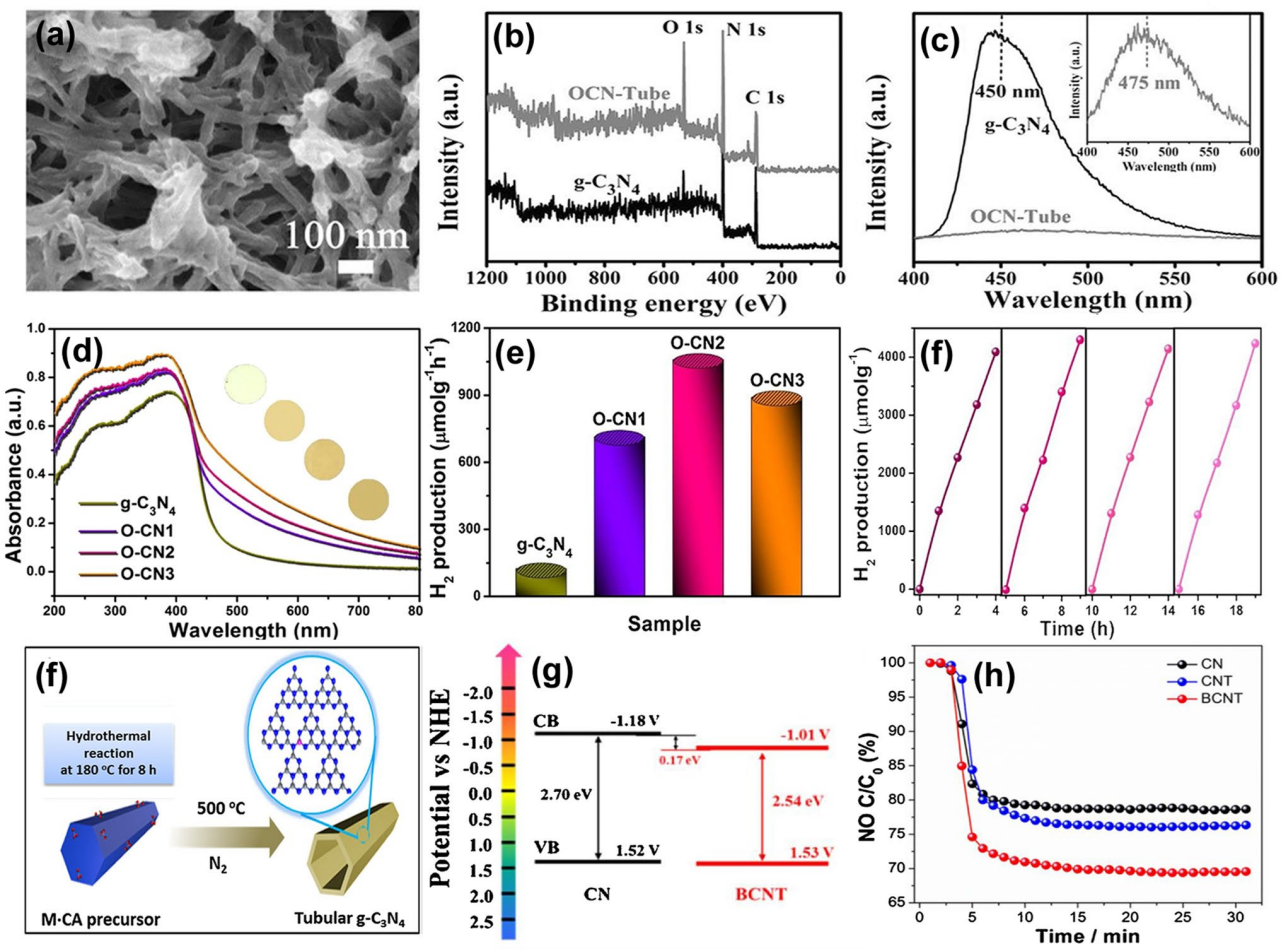
Defect control of O/B dopants.** a** Typical FESEM of OCN-Tube;** b** XPS survey spectra and** c** PL spectra of g-C_3_N_4_ and OCN-Tube [174]; Copyright 2017, Wiley–VCH. **d** UV–vis DRS and** e** HER rates of g-C_3_N_4_ and O-CNx samples;** f** stability test of O-CN2 under visible-light irradiation (*λ* > 420 nm) [175]; Copyright 2018, Elsevier.** g** Schematic synthetic diagram of tubular BCNT; **h** band structure of CN and BCNT;** i** photocatalytic NO removal activities of CN, CNT, and BCNT under visible-light irradiation [176]. Copyright 2018, Elsevier

Fu and colleagues prepared the hierarchical porous O-doped nanotubes (OCN-Tubes) by the successive high-temperature etching and curling-condensation of bulk g-C_3_N_4_ [174]. Due to the defect regulation, OCN-Tubes were not only doped by O atoms but exhibited a porous nanotube structure with an enhanced specific surface area of 36 m^2^ g^−1^ (Fig. 13a, b). Furthermore, it also displayed an enhanced visible-light absorption as the PL peak increased from 450 to 475 nm. Also, the suppressed photocarrier recombination of OCN-Tubes was shown according to the dramatically reduced PL intensity (Fig. 13c). The CH_3_OH yield of OCN-Tube in CRR activity is 0.88 μmol g^−1^ h^−1^, far more exceeding that of bulk g-C_3_N_4_ (0.17 μmol g^−1^ h^−1^). Lu et al*.* prepared O-doped g-C_3_N_4_ (O-CN*x*, *x* = 1, 2, 3 representing the molar ratio of ammonium acetate/melamine of 5, 10, and 30) via a direct thermal polymerization of melamine and ammonium acetate [175]. In good accordance with the above-mentioned studies, the O-CN2 showed a dramatically reduced bandgap by 0.52 eV in comparison with the bulk one. This was also verified by the extended visible-light absorption edge in Fig. 13d, which was inferred to boost the photocatalytic performance. As expected, the as-prepared O-CN2 displayed a 10-time-higher HER of 1062.4 μmol g^−1^ h^−1^ than that of bulk g-C_3_N_4_ (Fig. 13e). In addition, good cycling stability of O-CN2 for photocatalytic H_2_ production is also observed in Fig. 13f.

#### B Dopants with Narrowed Bandgap

Wang’s group synthesized the B-doped g-C_3_N_4_ nanotubes (BCNT) via thermal pyrolysis of H_3_BO_3_ and melamine (Fig. 13g) [176]. The unique BCNT structure with 0.3 μm thickness of the tube wall further improved its BET surface area from 17.8 to 27.9 m^2^ g^−1^. Compared with bulk CN, BCNT showed a similar VB position at around 1.53 V vs. NHE but a more negative CB position by 0.17 eV (Fig. 13h), indicating a narrowed bandgap that would allow more electrons to be generated under the same circumstances, and thus there were more ·O_2_^−^ radicals for NO removal. Due to the B-doping, the photocatalytic NO degradation rate of BCNT was, therefore, the best value of 30.4% within 30 min when irradiated by visible light, which was 10% larger than that of bulk g-C_3_N_4_ (20.8%, Fig. 13i).

#### Halogen Dopants with Narrowed Bandgap and Electronic Polarization

Halogen doping (F, Cl, I, Br) has been the research hotspot since the first pioneering work on F-doped g-C_3_N_4_ by Wang’s group in 2010 [177]. They speculated the F atoms preferred to bind with C atoms due to the electronegativity difference, and this would lead to the partial conversion of C-*sp*^2^ to C-*sp*^3^ followed by a decreased in-planar order. Their DFT calculations demonstrated that the F dopants in the bay C sites extended both the HOMO and LUMO to higher positions. While the corner C sites made the LUMO to higher energy levels, the HOMO lower energy levels. The experimental results showed the F-doped g-C_3_N_4_ boosted the photocatalytic oxidization of benzene to phenol in the presence of visible light. These results indicated that F dopants were critical to changing the electronic band structure of g-C_3_N_4_ and provided the basement for further redox modification. This work was also consistent with the published work by Ding and co-workers, who also investigated the F-doped g-C_3_N_4_ had a larger bandgap of 2.81 eV than bulk g-C_3_N_4_ of 2.68 eV [178]. Moreover, the authors also claimed that the B/F co-doped g-C_3_N_4_ not only met the demand of non-induced recombination centers plus enhanced solar light absorption but satisfied the requirement of overall water splitting with overpotentials.

**Fig. 14:**
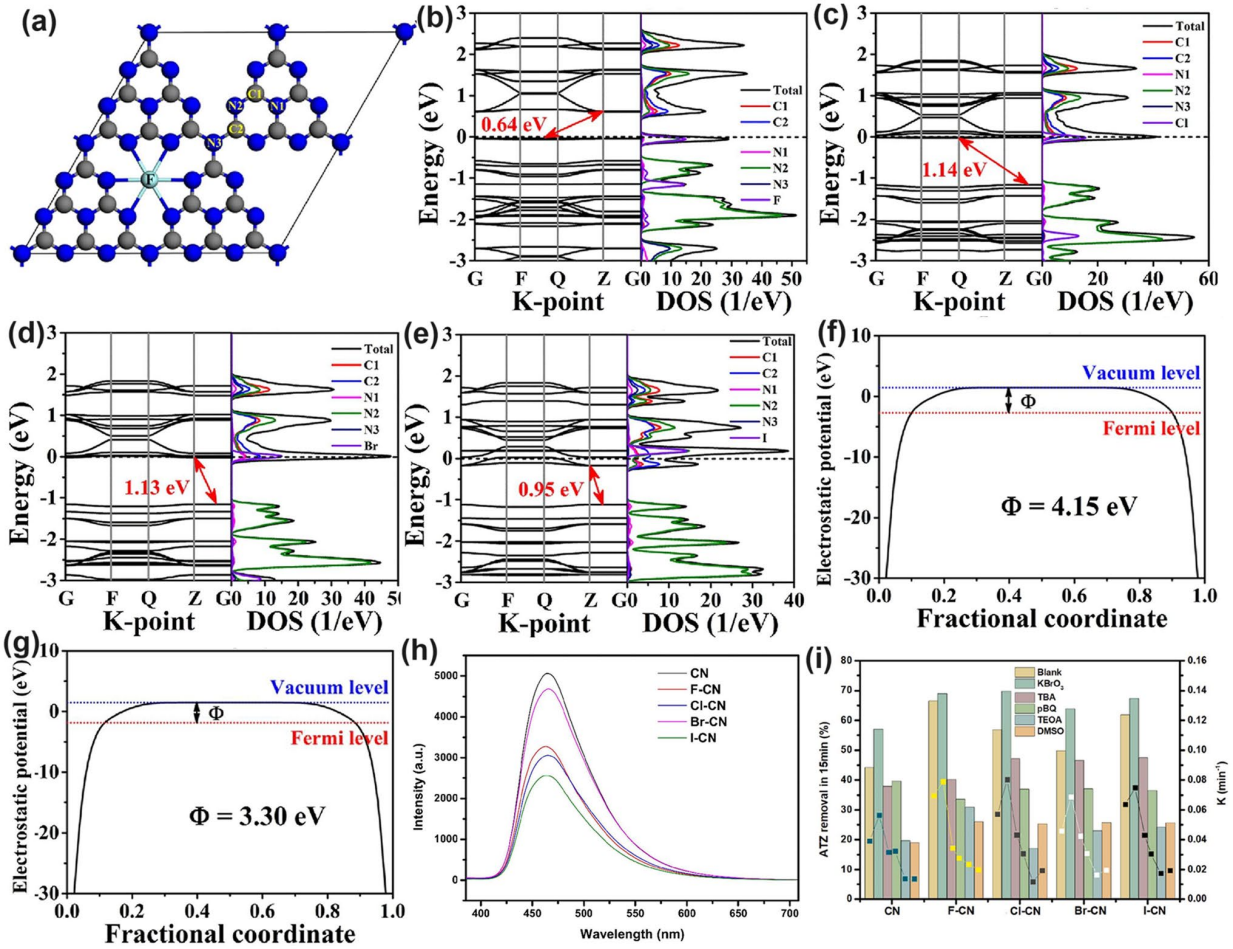
Defect control of halogen dopants. **a** Calculated F-doped g-C_3_N_4_ models in interstitial space; calculated band structures and DOSs of **b** F-C_3_N_4_; **c** Cl-C_3_N_4_; **d** Br-C_3_N_4_; **e** I-C_3_N_4_; work functions of **f** F-C_3_N_4_; **g** I-C_3_N_4_ [109]. Copyright 2017, Elsevier. **h** PL intensity; **i** ATZ removal rate comparison of various halogen atom-doped g-C_3_N_4_ [179]. Copyright 2022, Elsevier

Yu et al. found different halogen-doping positions in the g-C_3_N_4_ monolayer using the first principle investigation [109]. Specifically, they found F and Cl atoms preferred to be presented in the interstitial space due to the smallest formation energy of 1.15 and 3.52 eV, which was particularly dramatically lower than the N3/C2 sites, respectively (Fig. 14a). Actually, this was not strictly truth which they were not impossible to doping into N2 sites owing to the slightly higher formation energy (1.53 and 3.77 eV) compared to those of the interstitial space. Other halogen atoms of Br and I have a 1.6–2.6 times larger atomic radius than F and Cl, making them very unstable if directly displacing C and N atoms. Therefore, all halogen atoms were the most thermally stable in the interstitial space. Additionally, the electronic bandgaps were, in a rating order of 0.64, 0.95, 1.13, and 1.14 eV for F, I, Br, Cl-doped g-C_3_N_4_, which suggested a promoted solar harvesting ability than bulk g-C_3_N_4_ (Fig. 14b–e). Furthermore, with the bigger atomic number and higher electronegativity, the work function (Ф, calculated using the equation of $\Phi ={E}_{{\text{Vac}}}-{E}_{F}$ (where *E*_vac_ and EF are positions of vacuum level and Fermi level) became smaller from 4.15 to 3.30 eV (Fig. 14f–g), implying the easier for electrons to escape. As a result, these halogen atoms doped g-C_3_N_4_ exhibited an extended light absorption even to 1000 nm.

Recently, a deep and systematic study on the halogen-doped g-C_3_N_4_ has also been reported on photocatalytic ozonation (PCO) to remove the atrazine (ATZ) [179]. An increasing ATZ removal order for CN < Br–CN < Cl–CN < I-CN < F–CN was witnessed, which was also basically consistent with the bandgap order from the above-mentioned calculations by Yu’s group [109]. The reasons were ascribed to (1) All halogen-doped g-C_3_N_4_, particularly the F-CN, showed a narrowed bandgap at around 2.61 eV, enhancing its visible-light absorption; (2) DFT calculations revealed that the uneven distribution of electrons on halogen-doped g-C_3_N_4_ benefited the gas absorption. Among them, F-CN showed the highest O_3_ and O_2_ absorption energies of − 5.53 and − 4.55 eV, which further boosted the redox reaction kinetics; (3) the F-CN also displayed the largest water contact angle of 54.8°, which implied the optimized hydrophobicity merit that was more favorable for O_3_ absorption; (4) the photocarrier recombination of these doped g-C_3_N_4_ was significantly suppressed as reflected by the reduced PL intensity when compared to bulk g-C_3_N_4_ (Fig. 14h). These four factors combined to render the halogen-doped g-C_3_N_4_ with an effective ATZ removal rate of up to 66.5% under visible light (Fig. 14i). Other works reported in recent years agreed well with this discovery, revealing the promising applications of halogen doping on g-C_3_N_4_ for improving photocatalytic performance [177, 180–182]. Table 2 summarizes recent reports on non-metal doped g-C_3_N_4_ toward various solar applications.

#### Co-doping with Synergistic Effects

**Fig. 15:**
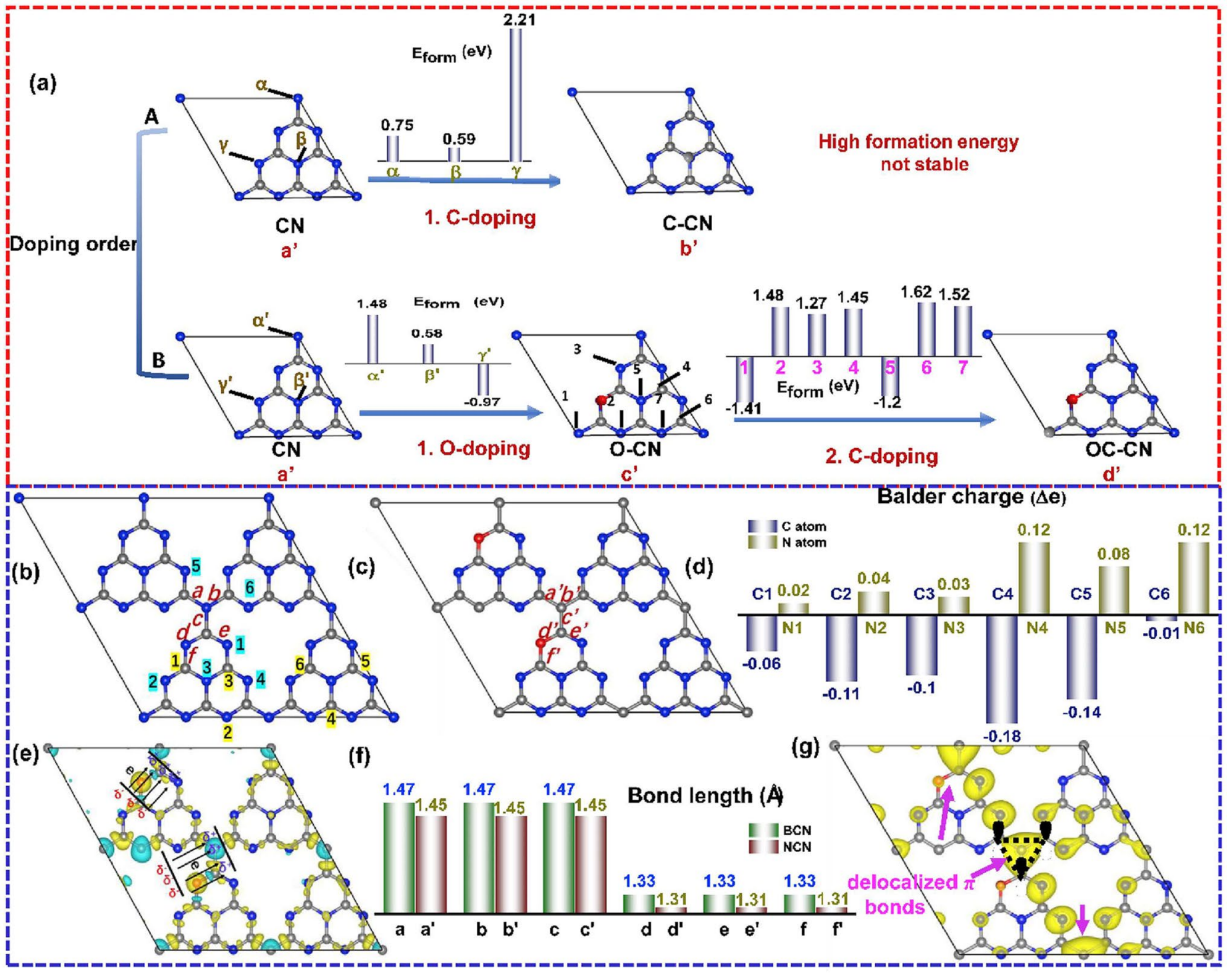
Defect control of co-doping. **a** Illustration of various C(O)-doped g-C_3_N_4_ with different doping order: (a’) CN; (b’) C–CN; (c’) O-CN and (d’) OC-CN; **b** configuration of original non-doped BCN; **c** optimized configuration of NCN; **d** Bader charge change; **e** differential charge density between BCN and NCN; **f** bond lengths of BCN and NCN; **g** *π* orbital distribution (VBM-5) of NCN (C, N and O atoms are shown in grey, blue and red. Olive and cyan illustrate the increase and decrease of electron distributions) [104]. Copyright 2019, Elsevier

Single-element doping enabled the optimization of the electronic band structure and photocarrier transfer progress of g-C_3_N_4_. Intriguingly, the heteroatomic co-doping that could combine the merits of these single dopants is also efficient in boosting its photocatalytic activity [158, 183, 184]. For instance, Ma et al. prepared P and O co-doped g-C_3_N_4_ that exhibited enhanced RhB photocatalytic degradation efficiency [185]. The B/F co-modified g-C_3_N_4_ also showed promoted HER performance[186]. A typical C/O-doped g-C_3_N_4_ synthesized from the calcination of protonated melamine has also attracted extensive research attention as its detailed information on both experimental results and calculations including the doping sites, bond length, and changed charge density distribution [104]. Taking the doping position firstly for example, Gao et al. found O dopants might be more favorable than C dopants at the first doping progress due to the unstable C-doped g-C_3_N_4_ with positive formation energy from 0.75 to 2.21 eV (Fig. 15a). As for first O-doping and then C-doping, the values for defective g-C_3_N_4_ could reach the least values of − 0.97 and − 1.2 eV, indicating a more spontaneous doping process. After comparing the electron density around the defects for both bulk g-C_3_N_4_ and C/O-doped g-C_3_N_4_, one can see more electrons were accumulating on the N4 sites and fewer electrons around C2 sites, indicating an enhanced electronic polarization effect (Fig. 15b–d). This would act as an “inner-built electric field” that can accelerate electron transfer with a driving force like Coulombic force (Fig. 15e). In addition, the bond length around these O and C dopants were all shortened by 0.02 Å, which was more beneficial for mass diffusion and charge transfer (Fig. 15f). More importantly, due to the C-doping into the N_3C_ sites, a delocalization *π* bond was formed, which enabled the excited electrons transfer among the tri-s-triazine units with better electronic conductivity (Fig. 15g). As a result, we observed a boosted photocurrent density and HER rate of 320 μA cm^−2^ and 830.1 μmol g^−1^ h^−1^, which were 60 and 7 times higher than those of bulk g-C_3_N_4_.

#### Dopants and Vacancies with Synergistic Effects

**Fig. 16:**
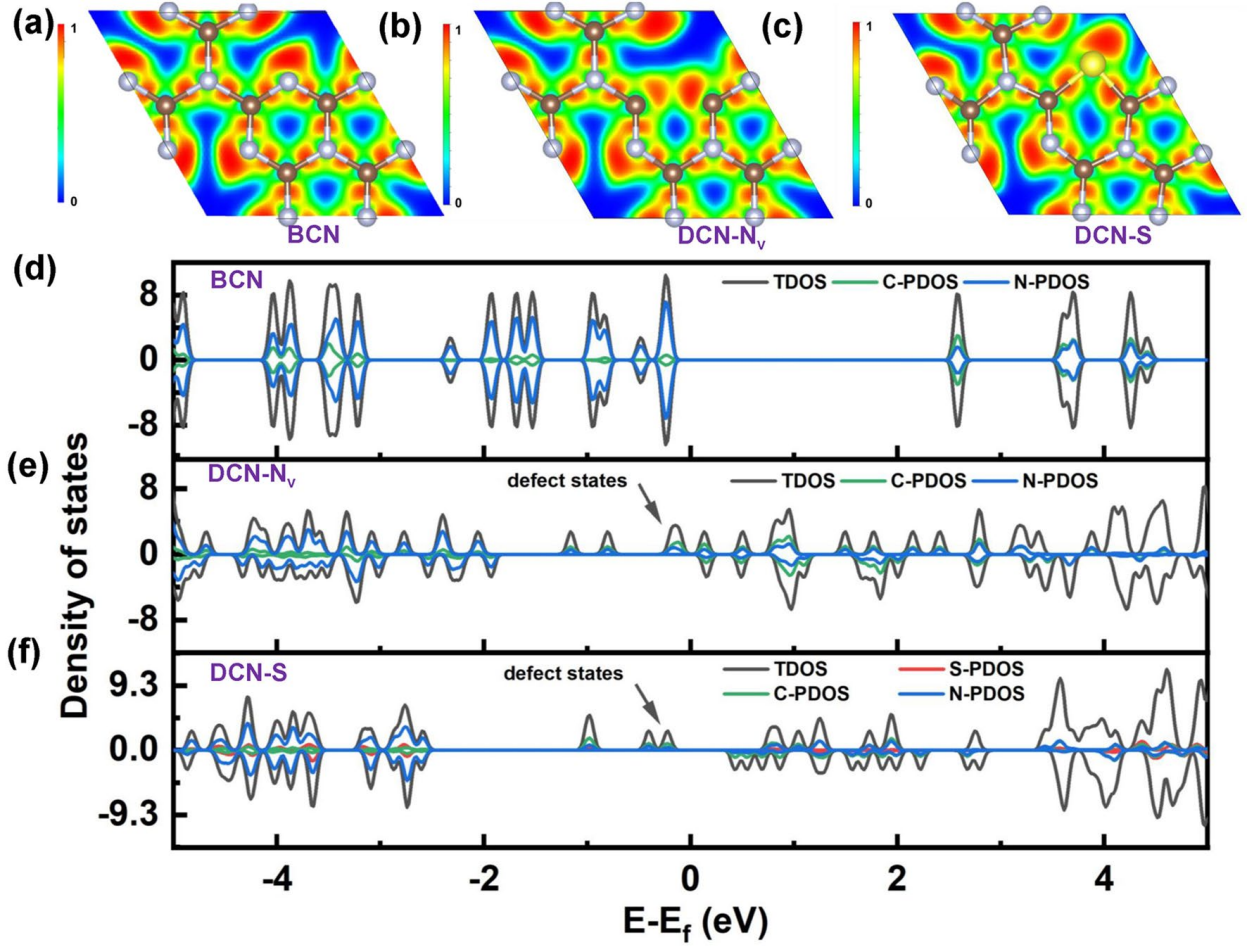
Defect control of dopant and vacancy. ELF plots of **a** BCN; **b** DCN-Nv; **c** DCN-S; total density of states (DOS) and partial density of states (PDOS) of **d** BCN; **e** DCN-Nv, and **f** DCN-S [91]. Copyright 2023, Wiley–VCH

Inspired by the research work combining different vacancies and dopants, our group also proposed a novel defective g-C_3_N_4_ (DCN) with both N vacancies and S dopants via a dual-solvent-assisted synthetic strategy [91]. Employing the protonated melamine obtained in the presence of glycol via a solvothermal reaction as the precursor followed by a subsequent annealing process with molten sulfur at 550 °C under N_2_ atmosphere, we determined DCN with an S-doping level of 0.5% and a moderate N vacancy concentration. With this defect control, DCN also exhibited a porous prisms nanostructure of 500 nm and an enhanced BET surface area of 169.10 m^2^ g^−1^, boosting the active sites for photocatalytic HER activity. Furthermore, the glycol and molten sulfur solvents were both critical to inducing N vacancies and S dopants and induced both shallow defect states and optimized surface states. The former could be revealed by the experimental defective energy levels which were 0.49 eV to the CBM of DCN. More importantly, in theoretical calculations, bulk g-C_3_N_4_ (BCN, Fig. 16a), g-C_3_N_4_ unit with one N vacancy (DCN-N_V_, Fig. 16b), and g-C_3_N_4_ unit with one S dopant (DCN-S, Fig. 16c), were proposed to unveil the different roles of N vacancy and S dopant. According to the ELF results (Fig. 16a–c), DCN-S showed the densest electron density toward the C_3_N_4_ unit cell void, corresponding to one of the lone pair electrons of S. This indicated DCN-S was more favorable to boost the electron polarization effect that could enable an accelerated photocarrier transport. As for the DOSs, we can see both DCN-Nv and DCN-S displayed new defect states around the Fermi level (Fig. 16d–f). However, the N vacancies in DCN pushed these additional energy levels closer to the VBM in comparison with the S dopants, indicating too much N vacancy concentration would worsen the electron band structure with deep localized states to severely recombine the photocarriers. This was also in good line with the XPS result that DCN displayed a moderate peak area ratio of –C_3_N/C=N–C at around 0.287. Furthermore, this S-doped and N vacant g-C_3_N_4_ also reveal optimized surface states with the highest surface trapping resistance (*R*_trapping_) of 9.56 × 10^3^ Ω cm^2^ and the slowest decay kinetics of surface carriers (0.057 s^−1^), which guaranteed the smooth surface charge transfer rather than being the recombination sites. As a result, it exhibited a superior H_2_ evolution rate of 4219.9 µmol g^−1^ h^−1^, which was 29.1-fold higher than unmodified g-C_3_N_4_.

**Fig. 17:**
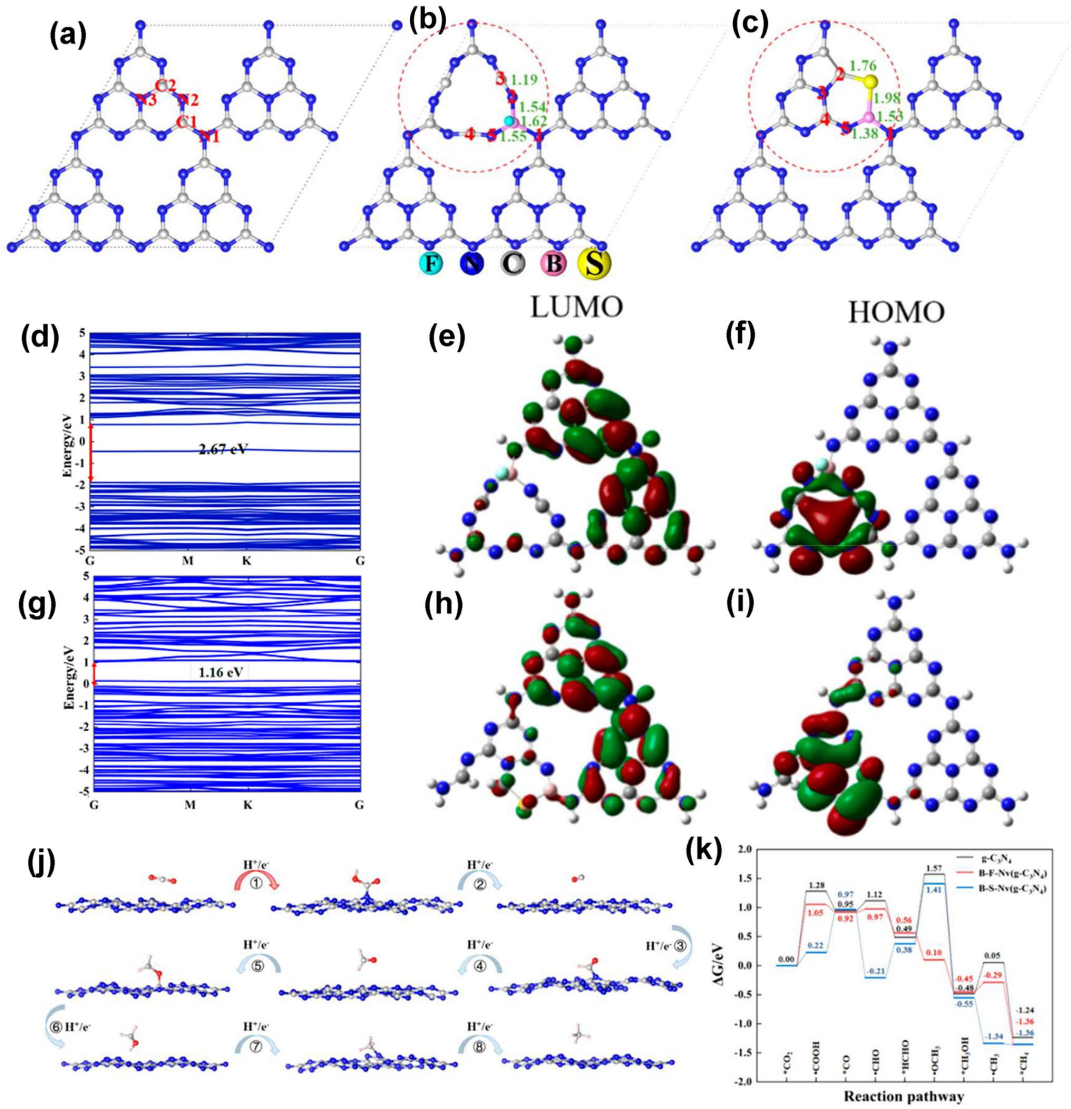
Defect control of multiple defects type in g-C_3_N_4_. **a** Top view of pristine g-C_3_N_4_ geometries; Optimized geometries of **b** B–F–N_v_ and **c** B–S–N_v_; calculated band structure of **d** B–F–N_v_; **e** LOMO for B–F–N_v_; **f** HOMO for B–F–N_v_; **g** calculated band structure of B–S–N_v_; **h** LOMO for B–S–N_v_; **i** HOMO for B–S–N_v_; **j** Gibbs free energy diagrams of photocatalytic CO_2_ reduction; **k** representative geometries of the stable points in the specific reduction process for CO_2_ on g-C_3_N_4_ [187]. Copyright 2022, Multidisciplinary Digital Publishing Institute

In another typical work, multiple defects-modified g-C_3_N_4_ catalysts with B-F or B-S co-doping combined with N vacancies (donated as B–F–N_v_, B–S–N_v_) have also shown great potential for the optimization of electronic band structure and enhancement of photocatalytic CRR performance [187]. As shown in Fig. 17a, there were two C and three N doping positions assigned to C1, C2, N1, N2, and N3 which could be doped or vacant for B/F/N_v_. According to the formation energy, B and F preferred to be presented in the C1 site and connected with the N2 site. However, after introducing the Nv at N3 site, the F atoms would transfer from N2 to B side due to its strong electronegativity, leaving the rest C–N becoming into sp hybridization (Fig. 17b). However, as for B–S–N_v_, B and S atoms preferred to be at the C1 and N2 sites while Nv presented at new N2 site next to this unit, forming a five-ring unit to keep the structure stable (Fig. 17c). According to the DOSs diagrams, the bandgap of B/F co-doped g-C_3_N_4_ was found to be 3.06 eV, significantly higher than 2.77 eV of g-C_3_N_4_. However, due to the presence of N_v_, the bandgap of B–F–N_v_ material reduced to 2.67 eV, suggesting the N vacancy’s role in narrowing the bandgap with extended solar absorption (Fig. 17d). Interestingly, the S dopant was also significant in further reducing the bandgap of B–S–N_v_ to 1.16 eV than F dopant of B–F–N_v_ (Fig. 17g). Additionally, the HOMO and LUMO of B–F–N_v_ and B–S–N_v_ have little overlap (Fig. 17e, f, h, i), which can effectively facilitate the separation of photogenerated electrons and holes. Furthermore, new electron distribution of HOMO and LUMO on bridging N atoms could promote the migration of photogenerated charge carriers, thereby enhancing solar utilization efficiency. The photocatalytic CO_2_ reduction reaction is a complex process that usually generates multiple products. Figure 17j shows the specific reaction pathway of the photocatalytic CO_2_ reduction reaction:

*CO_2_ → *COOH → *CO → *HCHO → *OCH_3_ → *CH_3_OH → *CH_3_ → *CH_4_. Based on this, it can be concluded that the products generated in the photocatalytic CO_2_ reduction reaction were CO, HCHO, CH_3_OH, and CH_4_. Figure 17k provides the Gibbs free energies required for different catalysts at different stages of the photocatalytic CO_2_ reduction reaction. According to Δ*G* change, we can infer the main product of B–F–N_v_ for photocatalytic CO_2_ reduction reaction was CH_3_OH due to the following uphill energy (0.16 eV) for the conversion of *CH_3_OH to ·CH_3_. On the other hand, B–S–N_v_ had a lower Δ*G* change for catalyzing the CO_2_ reduction reaction, facilitating the reaction to proceed to the final step and generate CH_4._ This would inspire future researchers to tune the defect types to enhance product selectivity.

**Table 2:** Comparison of non-metal doped g-C_3_N_4_ toward various solar applications

g-C_3_N_4_	Dopant	Light source	Solar application	Photocatalytic activity	Refs.
CNS	S (N_2C_)	*λ* > 400 nm	HER	121.6 mmol g^−1^ h^−1^	[169]
CN-MT	S (N_2C_)	*λ* > 400 nm	HER	5000 mmol g^−1^ h^−1^	[188]
Coral-2.5B-CN	B (N_3C_)	*λ* > 420 nm	H_2_O_2_ formation	314.55 μmol g^−1^ h^−1^	[189]
SC_3_N_4_	S (N_2C_)	*λ* > 420 nm	Pollutant removal (UO_2_^2+^)	1.6 min^−1^ g^−1^	[171]
SCNNS_S_	S (N_2C_)	Visible light	NRR	5.99 mmol g^−1^ h^−1^	[32]
CN-Br	Br (N_3C_)	*λ* > 400 nm	HER	2400 μmol g^−1^ h^−1^	[190]
MCN	O (N_2C_)	420–800 nm	HER	1204 mmol g^−1^ h^−1^	[75]
g-C_3_N_4_	O (N_3C_)	*λ* > 420 nm	HER	1430 μmol g^−1^ h^−1^	[191]
CN0.75	N (C_3N_)	*λ* > 400 nm	TC removal	26.94 min^−1^ g^−1^	[162]
p-CN	O (N_3C_)	*λ* > 420 nm	HER	395.96 μmol g^−1^ h^−1^	[192]
OPCN	O (N_2C_)	420–780 nm	BPA removal	0.7 min^−1^ g^−1^	[193]
O-CNC	O (N_3C_)	*λ* > 420 nm	H_2_O_2_ formation	2008.4 μmol g^−1^ h^−1^	[194]
CNS-TiO_2_/g-C_3_N_4_	S (n/a)	Visible light	RhB removal	1.6 min^−1^ g^−1^	[195]
O-CN	O (N_2C_)	*λ* > 420 nm	HER	1062.4 μmol g^−1^ h^−1^	[196]
PCNNF_S_	P (n/a)	*λ* > 420 nm	HER	9546 μmol g^−1^ h^−1^	[197]
PC_3_N_4_	P (N_2C_)	*λ* > 420 nm	Pollutant removal (UO_2_^2+^)	1.1 min^−1^ g^−1^	[198]
OPCN	O (N_3C_)	*λ* > 400 nm	H_2_O_2_ formation	16.7 μmol g^−1^ h^−1^	[199]
CN-SP	P (N_3C_)	*λ* > 420 nm	HER	570 μmol g^−1^ h^−1^	[200]
B-g-C_3_N_4_	B (n/a)	*λ* > 420 nm	Pollutant removal (UO_2_^2+^)	0.52 min^−1^ g^−1^	[201]
BCN-0.75	B (C_3N_)	*λ* > 420 nm	HER	1639.28 μmol g^−1^ h^−1^	[202]
SOCN	S (N_2C_)/O(N_3C_)	Sun light	MO removal	0.29 min^−1^ g^−1^	[203]
P-Nv-C_3_N_4_	P (N_3C_)	simulated sunlight	NRR	1686.4 μmol g^−1^ h^−1^	[204]
P/UN-CNS	P (C_3N’_)	*λ* > 420 nm	HER	9653 μmol g^−1^ h^−1^	[205]
S-g-C_3_N_4_-E	S (N_3C,_N_2C,_N_3c’_)	*λ* > 420 nm	HER	5548.1 μmol g^−1^ h^−1^	[204]
BCN	B (C_3N_)	*λ* > 420 nm	HER	1.64 mmol g^−1^ h^−1^	[206]
CN-T-U	S (n/a)	*λ* > 420 nm	RhB removal	0.7352 min^−1^ g^−1^	[207]
4Ce/CN	N/O (n/a)	Visible light	NO removal	1.34 min^−1^ g^−1^	[208]

n/a indicates there is no discussion on dopant sites in original publications

## Metallic doping with Active Coordinate Environment

Generally speaking, the metallic dopants in g-C_3_N_4_ normally induce enhanced solar light absorption, fast electron transfer, and high photocarrier separation efficiency [158]. Metal doping is usually realized by the thermal pyrolysis of the mixture of g-C_3_N_4_ precursors and a soluble metal salt. In the early stage of metallic doping, researchers have not specified the metal existence form of either in nanoclusters aggregation or atomic distribution as the limits of ordinary TEM and XPS technologies. This situation has changed since the employment of the special aberration-corrected transmission electron microscope (AC-TEM) and K-edge X-ray absorption fine structure (EXAFS) that can distinguish the metal morphology at the atomic level with very high resolution and identify the metal coordination environment with both interaction and bonding species [209]. For the former, one can clearly see whether metal is in small aggregation or atomic well-dispersion. For the latter, researchers need to analyze the spectrums to figure out the only metal interaction such as metal-N peaks without any other peaks such as metal–metal peak, metal-oxide peak, and so on. Based on the above knowledge, the development of metallic-doped g-C_3_N_4_ toward various photocatalytic applications was extended as follows:

### ***Alkali Metallic doping with M***–N_***x***_*** Bonding***

The typical alkali metals, such as K and Na dopants, were found to exhibit different roles in regulating the electronic band structure and optical properties of g-C_3_N_4_ [210–212]. In a detailed theoretical study, Xiong et al*.* found both K and Na atoms can narrow the bandgap and strengthen the solar light absorption of g-C_3_N_4_ [210]. Additionally, K atoms preferred to be presented in the interlayer space and thus provided electrons with better vertical transfer pathways. In contrast, the Na atoms preferred to chemically bond with the in-planar N atoms via ion bond due to the easy escaping of Na 3 s electrons. Based on their experimental results, the K-doped g-C_3_N_4_ reflected a better photocatalytic NO removal activity than the Na-doped one, suggesting the prominent role of K-doping over Na-doping.

**Fig. 18:**
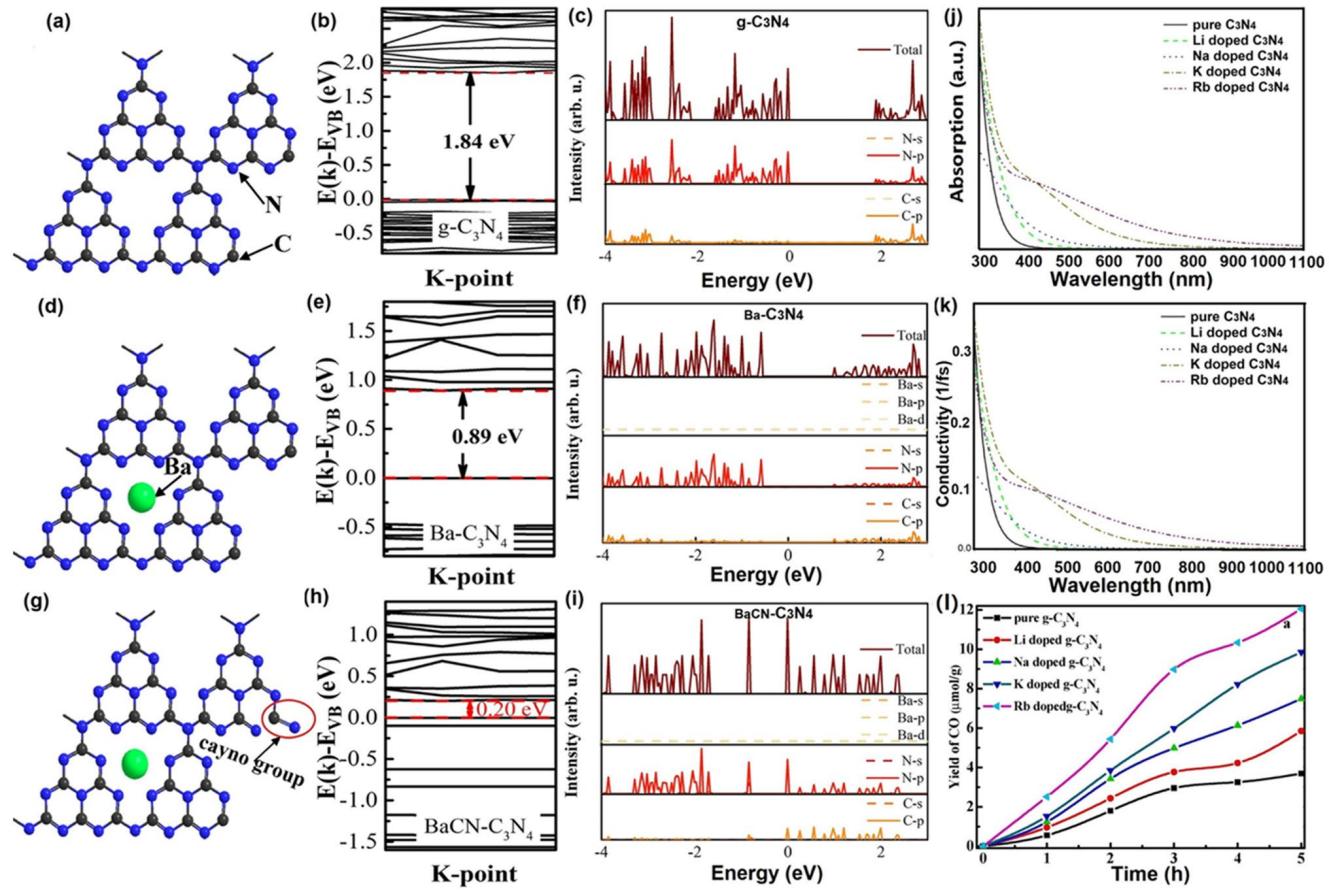
Defect control of alkali metallic doping. **a, d, g** Structural models; **b, e, h** calculated band structures and **c, f, i** PDOS spectra of g-C_3_N_4_, Ba-C_3_N_4_ and BaCN-C_3_N_4_ [89]. Copyright 2021, Elsevier.** j** Calculated UV–vis DRS; **k** calculated conductivity curves and** l** CO evolutions of pure g-C_3_N_4_ and various alkali metal doped g-C_3_N_4_ under full-spectrum irradiation [213]. Copyright 2020, Elsevier

Other alkali metals, such as Ba and Rb, have also been verified to be efficient in boosting the solar activity of g-C_3_N_4_ [89, 213]. For instance, Hu et al*.* employed a facile salt-assisted method to synthesize the Ba-doped g-C_3_N_4_ (BaCN-C_3_N_4_) [89]. The successful Ba-doping was revealed by the obvious Ba 3*d* signal according to the full-scan XPS spectrum. Compared to the original g-C_3_N_4_, BaCN-C_3_N_4_ displayed an extended optical absorption edge by 10 nm and its bandgap was slightly narrowed by 0.05 eV, showing the improved visible-light absorption due to Ba dopants. The DFT calculations revealed that due to the insert of Ba dopants into the g-C_3_N_4_ heptazine ring cavity, its bandgap can be dramatically reduced from 1.84 to 0.89 eV (Fig. 18a-f). It is worth mentioning that the presence of BaCl_2_ during the pyrolysis process could induce the formation of cyano groups, which further lowered the BaCN-C_3_N_4_ to 0.2 eV (Fig. 18g-i). However, the CBM and VBM of these samples were all contributed by C 2*p* and N 2*p* orbits, and Ba was not involved in the construction of the band edge structure (Fig. 18c, f, i). The authors also claimed that this narrowed bandgap of BaCN-C_3_N_4_ might be aroused by the reduced corrugation amplitude due to Ba incorporation. Combined with the electronic polarization around Ba dopants and cyano groups, the charge transfer dynamics can be further boosted. As a result, the 7%-BaCN-C_3_N_4_ achieved an excellent tetracycline (TC) degradation rate and HER rate of 63.6% and 10,316 μmol g^−1^ h^−1^, significantly, exceeding the bulk g-C_3_N_4_. Another piece of systematic work proposed by Zhang et al*.* indicated that Rb atoms are the best alkali dopants in promoting the photocatalytic activity of g-C_3_N_4_ [213]. It can be seen from Fig. 18j that Rb-doped g-C_3_N_4_ exhibited the most extended solar harvesting ability with the lowest bandgap of 2.0 eV, which was 0.65 eV smaller than pristine g-C_3_N_4_, implying the substantially improved visible-light absorption. Moreover, the Rb-doped g-C_3_N_4_ also reflected the best conductivity in the solar range from 300 to 1100 nm, demonstrating the fastest charge transfer kinetics, which might favor the redox reaction (Fig. 18k). Along with its merit of the lowest electron transport barrier, Rb-doped g-C_3_N_4_ displayed the highest CO yield of 12.1 μmol g^−1^ (Fig. 18l). Therefore, based on the above experimental results and DFT calculations, we can conclude that the alkali metals, particularly Rb-doped g-C_3_N_4,_ hold great prospect for g-C_3_N_4_-based solar applications.

### ***Transition Metallic doping with M***–N_***x***_***, M-C***_***2***_***N***_***2***_***, M–O Bonding***

Apart from alkali metals, transition metals such as Co, Cu, Fe, Ce, and Bi have also been verified to promote the photocatalytic activity of g-C_3_N_4_ [158, 183]. For example, Deng et al*.* used nickel formate and urea as raw materials to prepare the Ni-doped g-C_3_N_4_ samples (NCN-X) [214]. For NCN-1, it presented the porous nanosheet structure with folded and rolling edges with a thickness of 10 nm. The Ni dopants were well distributed on the g-C_3_N_4_ surface as reflected by the energy dispersive spectroscopy (EDS). With the increasing Ni content, NCN-X displayed not only an enhanced harvesting ability of solar light but also a longer absorption edge with decreasing bandgaps of 2.73, 2.68, 2.61, and 2.45 eV for CN, NCN-1, NCN-2, and NCN-3, respectively. Combined with the Mott–Schottky results, the VB potentials of NCN-2 reached the maximum value of 1.59 eV, which was conducive to the transfer of photoexcited carriers and charge separation progress. Therefore, we can observe NCN-2 displayed the highest MO degradation rate of 97.3% within 90 min and the highest HER rate up to 155.71 μmol g^−1^ h^−1^ among all the g-C_3_N_4_-based photocatalysts.

**Fig. 19:**
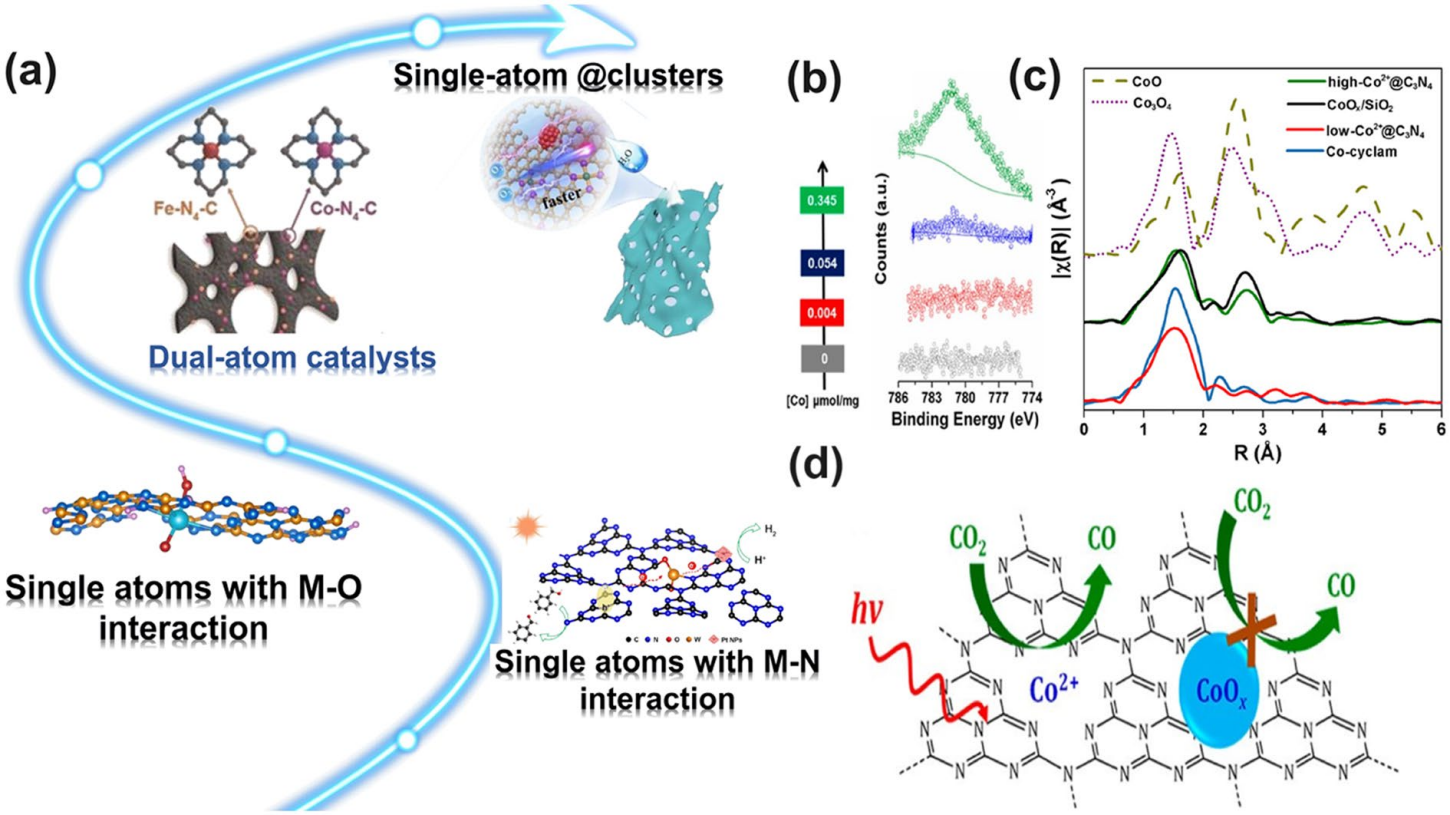
Defect control of SAs-doped g-C_3_N_4_. **a** Research evolution of single atoms-doped g-C_3_N_4_ [223–226]; **b** XPS of bare C_3_N_4_ and Co^2+^@C_3_N_4_; **c** Fourier transform magnitude of k^2−^ weighted Co K-edge EXAFS spectra; **d** schematic diagram of photocatalytic CO_2_ reduction mediated by a single CO^2+^ site on C_3_N_4_ [227]. Copyright 2018, American Chemical Society

Although significant advances have been made in the research fields of metallic-doped g-C_3_N_4_ toward various solar applications[215, 216], the precise active sites between metallic dopants and g-C_3_N_4_, and the existing form of metals were still not clear. Recently, metal single atoms (M-SAs) have exhibited a promising prospect with stable stability in electrochemical catalysis such as water splitting [217, 218], CO_2_ reduction[219, 220], and so on [221, 222]. Since then, more research attention has been devoted to the M-SAs doped g-C_3_N_4_ systems, including identifying the metallic-nitrogen (M–N) interaction [223], metallic-oxygen interaction [224], forming the dual-atom catalysts [225] or even SAs@metal clusters catalysts (Fig. 19a) [226].

Taking the M–N interaction first, Co single atoms (SAs) were successfully doped into the g-C_3_N_4_ matrix forming the Co–N bonding via a microwave method in the presence of triethanolamine (TEA), and the resultant sample was labeled as Co^2+^@C_3_N_4_ [227]**.** The XPS confirmed the presence of Co^2+^ with an obvious peak assigning to Co 2p_3/2_ transition at 781 eV when the Co-doping level increased from 0.004 to 0.345 μmol per 1 mg g-C_3_N_4_ (Fig. 19b). Interestingly, the Co SAs were detected only in low-Co^2+^@C_3_N_4_ as reflected by the Co–N interaction at 1.55 Å along with the absence of Co–Co at 2.7 Å according to the Fourier transform magnitude Co K-edge X-ray absorption fine structure (EXAFS) spectrum (Fig. 19c). While the high-Co^2+^@C_3_N_4_ samples showed similar peaks with CoO_x_/SiO_2_, implying the Co SAs were prone to aggregate into nanoparticles at high precursor concentrations. Furthermore, the g-C_3_N_4_ substrate was inferred to be the C-doped type rather than the O-doped type as reflected by the superior CRR performance of C-doped g-C_3_N_4_, which also coincided with the previous study [105]. Additionally, when the Co content reached 0.128 μmol mg^−1^, the CO yield achieved the highest value of 1.056 μmol mg^−1^. However, excessive Co content resulted in the formation of CoO_x_, endowing a lower CRR activity with low CO selectivity, demonstrating the superior redox selectivity of the single atomic Co^2+^ sites (Fig. 19e).

**Fig. 20:**
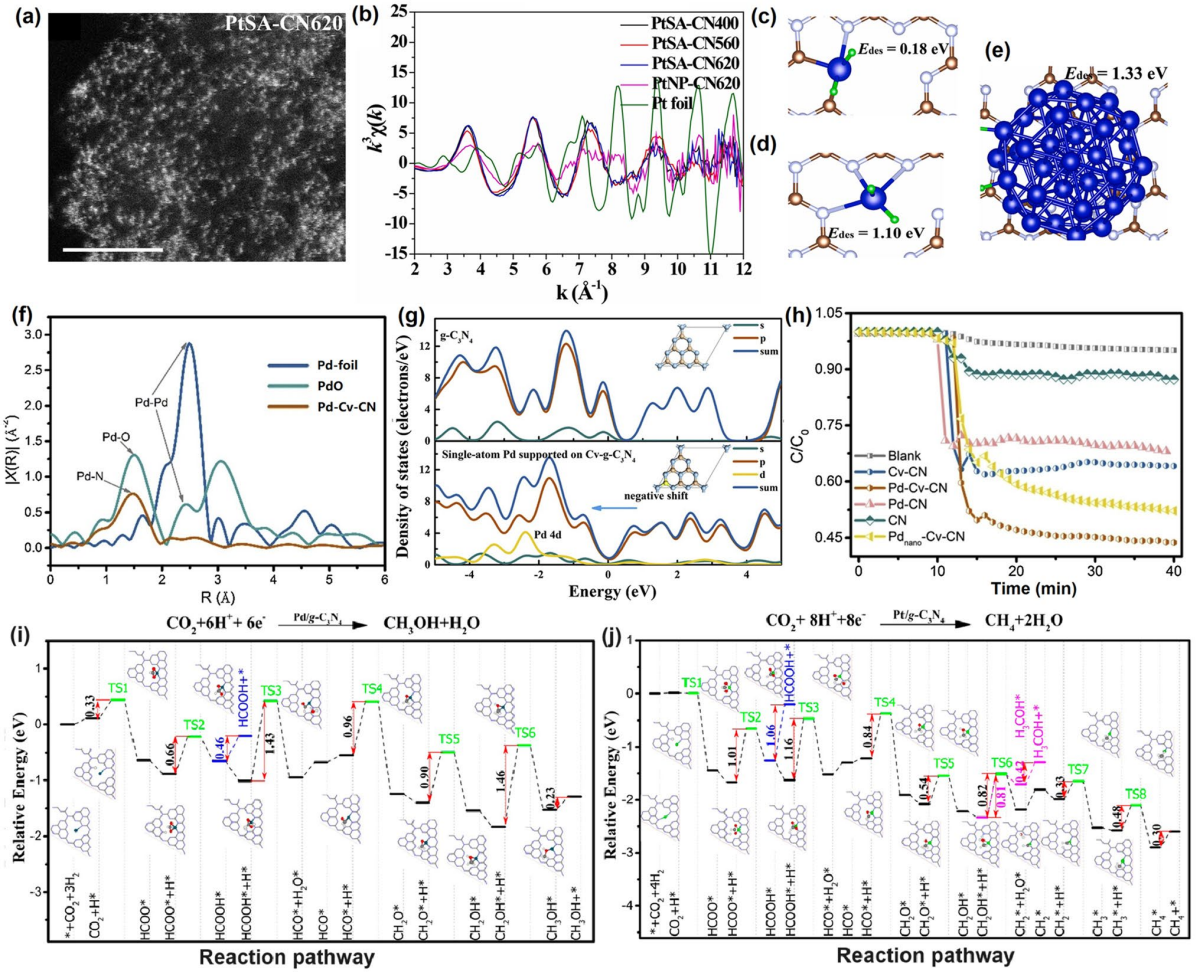
Defect control of single atoms doping. **a** High-angle annular dark-field scanning transmission electron microscope (HAADF-STEM) of PtSA-CN620;** b** k^3−^weighted FT spectra at k space of Pt foil and various PtSA-CN samples; H_2_ desorption on PtSA at** c** C_2C_ and **d** N_2C_ sites and **e** PtNP-C_3_N_4_ [228]**.** Copyright 2019, Elsevier.** f** FT of Pd-CV-CN, Pd foil, and PdO at Pd K boundary;** g** DOSs diagram of g-C_3_N_4_ and Pd-Cv-CN;** h** photocatalytic NO removal activities of various g-C_3_N_4_-based samples [97]**.** Copyright 2021, Elsevier. Reaction pathways for CO_2_ reduction to **i** HCOOH and CH_3_OH on Pd/g-C_3_N_4_ and **j** HCOOH, CH_3_OH, and CH_4_ on Pt/g-C_3_N_4_ catalyst (color code: Pd, pine green; C, gray; O, red; H, white) [229]. Copyright 2016, American Chemical Society

In another typical M-SAs work, the N vacant g-C_3_N_4_ synthesized at different temperatures were chosen as the metal deposition substrates as the N vacancies could stabilize Pt single atoms (PtSA) [228]. The as-prepared samples were named PtSA-CNX, where X was the annealing temperature. It is not surprising to see the resulting PtSA-CN620 exhibited a uniform PtSA coverage density of 0.35 mg m^−2^ without any obvious aggregates from the HAADF-STEM image (Fig. 20a), which was much better than those obtained in a lower temperature at 400 and 560 °C. According to the Fourier transform K^3^ weighted (EXAFS) spectrum, the wavelet transform (WT) maximum at 5.61 Å^−1^ of PtSA-CN620, PtSA-CN560, and PtSA-CN400 revealed that the existence of PtSA in the form of Pt-C, Pt–N, and Pt-O (Fig. 20b). Furthermore, the calculated H_2_ desorption energies on Pt nanoparticles (PtNP), and PtSA next to the two-coordination N (N_2C_) and C (C_2C_) sites were 1.33, 1.10 and 0.18 eV, respectively (Fig. 20c–e). The authors ascribed this to the high proton-reduce degree and shorted H distance on the C_2C_ sites, which was beneficial to the H_2_ evolution kinetics. For the optical property, the solar absorption of this PtSA-modified g-C_3_N_4_ was enhanced as the annealing temperature increased. Among them, PtSA-CN620 also displayed the most extended absorption edge to 640 nm with the smallest bandgap of 2.17 eV and the fastest photocarrier transfer dynamics with the lowest average photocarrier time of 3.40 ns, further demonstrating the superior role of PtSA. Thus, we observed the highest HER rate of 174.5 mmol g^−1^ h^−1^ per PtSA for PtSA-CN-620.

Inspired by this, the C-vacancy-rich g-C_3_N_4_ obtained at 600, 550, and 500 °C was also fabricated as the matrix for Pd SAs with the corresponding defective samples abbreviated as C_V_-CN, C_V_-CN-1, and C_V_-CN-2, respectively [97]. Owing to the large specific surface area of Cv-CN at around 92.0 m^2^ g^−1^, a high Pd SAs coverage density can be achieved. A deeper discussion on the chemical interaction was identified to be the Pd–N bond located near 2.03 Å according to the XAFS spectrums (Fig. 20f). This was due to the stabilization effect of C vacancies with Pd SAs, which also agreed well with the previous report [105]. The DOSs were analyzed to interpret how the Pt SAs affect the electronic band structure of Cv-CN. Compared to g-C_3_N_4_, Pd-Cv-CN exhibited more negative peaks with the changed peak shape on the negative side but similar peaks on the positive side (Fig. 20g). Therefore, Pd-Cv-CN showed lifted energy levels with a narrower bandgap. In addition, the impurity energy levels were ascribed to the regulation of Pd 4*d* orbit, which was supposed to boost solar utilization. Specifically, an inhibited PL intensity was shown for Pd-Cv-CN in contrast with Cv-CN, suggesting improved photocarrier separation. As a result, Pd-Cv-CN showed the highest NO removal efficiency of 56.3% within 30 min, which was 10.3% higher than the Pd nanoparticles-modified one (Fig. 20h), validating the critical role of Pd SAs toward photocatalysis.

Gao et al*.* compared the possible reaction pathways of photocatalytic CRR on the Pd SAs- and Pt SAs-modified g-C_3_N_4_ via the DFT calculations [229]. For Pd SAs-g-C_3_N_4_ (Fig. 20i): (1) The rate-determining step was the hydrogeneration of HCOOH* with a reaction barrier of 1.46 eV, which was believed to happen thermodynamically according to previous reports [230]. (2) Compared to the following high barrier (1.46 eV) for CH_3_OH generation, HCOOH^+^* was more likely to leave from Pd SAs-g-C_3_N_4_ due to its dramatically reduced desorption barrier of 0.46 eV. Therefore, the resulting final product for Pd SAs-g-C_3_N_4_ was HCOOH. However, regarding the Pt SAs-g-C_3_N_4_ (Fig. 20j): (1) The barrier of HCOOH* hydrogeneration was about 0.27 eV lower than that of Pd SAs-g-C_3_N_4_, indicating a more favorable reaction on Pt SAs surface. (2) The higher HCOOH^+^* desorption energy of 1.06 eV might imply the following hydrogeneration pathways were more thermodynamically favorable for both CH_3_OH^+^* and then CH_4_^+^* generation. Thus, the final CRR product on Pt SAs-g-C_3_N_4_ was suspected to be CH_4_. This intriguing work suggested that different types of M-SAs might have different roles in tailoring the redox selectivity, which was critical for future photocatalyst design.

**Fig. 21:**
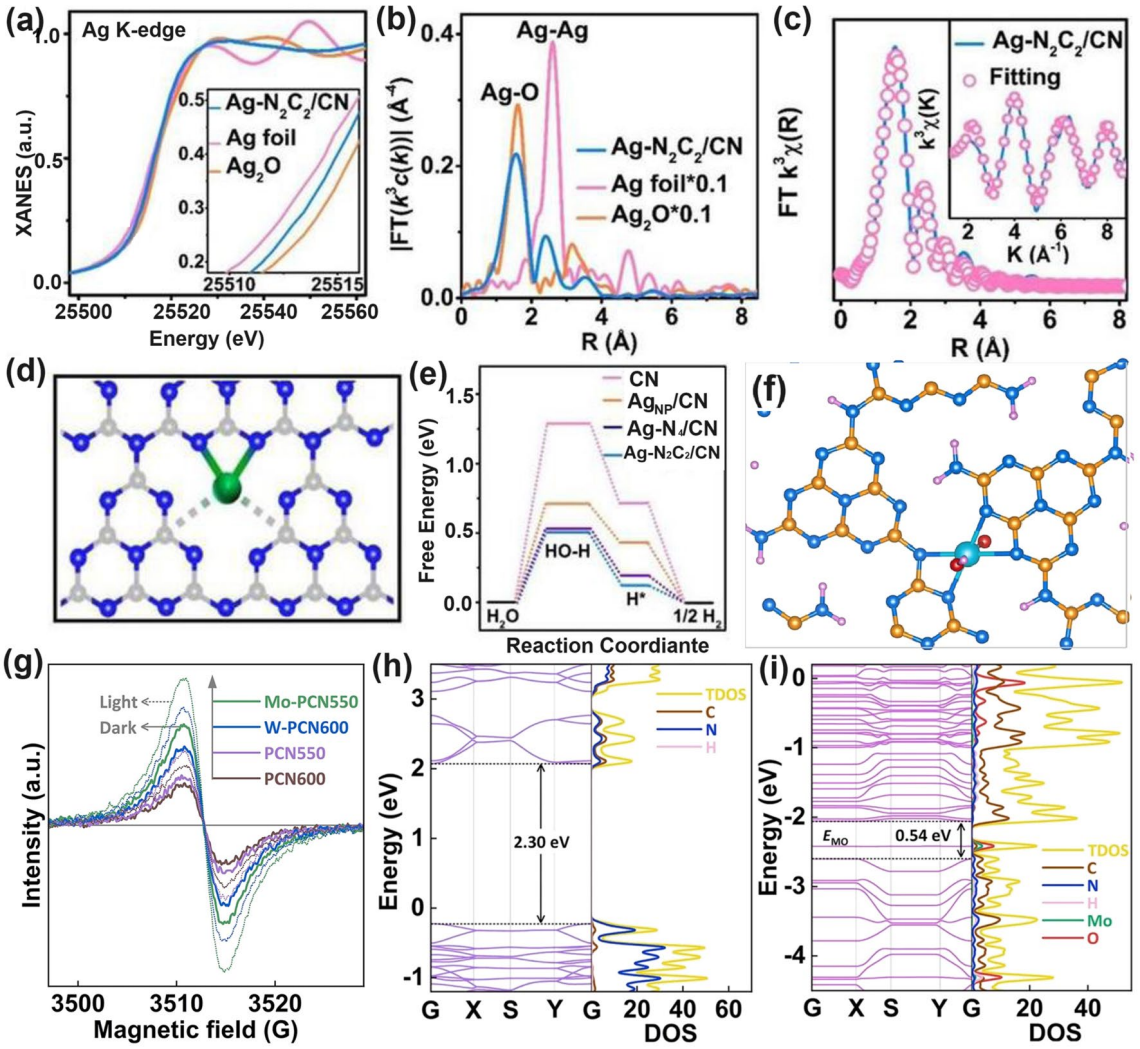
Defect control of single atoms dopants with other coordination types. **a** Ag K-edge XANES; **b **Fourier transform of Ag K-edge EXAFS spectra; **c **EXAFS fitting curve in R space and **d **Structure model of Ag–N_2_C_2_/CN (Ag green, N blue, C grey); **e** Free energy profiles for H_2_ evolution reactions over the as-prepared catalysts [111]. Copyright 2020, Wiley–VCH. **f** Simple framework structure of MO-PCN; **g** EPR spectra of the samples in dark and under irradiation; DOSs plots of **h** PCN and** i** Mo-PCN550 monolayers [224]. Copyright 2023, Elsevier

Apart from the widely investigated M–N interaction between SAs and the g-C_3_N_4_ matrix [231], researchers have found that M–N_2_C_2_ coordination for Ag SAs was more favorable to boosting the photocatalytic HER activity [111]. Specifically, the Ag–N_2_C_2_-modified g-C_3_N_4_ (Ag–N_2_C_2_/CN) was prepared via a novel annealing process with the self-assembled melamine cyanurate and Ag-containing salt. As reflected by the Ag K-edge XANES (Fig. 21a), the absorption line of Ag–N_2_C_2_/CN lied between Ag foil and Ag_2_O, indicating the oxidation state of Ag was between 0 to + 1 due to the strong interaction between Ag and tri-s-triazine units. According to the k^3^-weighted EXAFS result, there were only two main peaks at 1.55 and 2.41 Å, corresponding to the first coordination shell of Ag–N and the second coordination shell of Ag-C, respectively (Fig. 21b). Further EXAFS fitting result evidenced the coordination numbers of Ag–N and Ag-C were 2.3 and 1.8, demonstrating the Ag SAs generation to be coordinated with N_2_C_2_ interaction (Fig. 21c–d). This interaction significantly contributed to the electronic polarization effect around the Ag–N_2_C_2_ sites which was stronger than Ag–N and Ag nanocluster sites, indicating its superior role in accelerating the photocarrier transfer and separation. The Gibbs free energy required for each step in the reaction process of H_2_O → HO-H → H^*^ → H_2_ was also calculated. It was found that Ag–N_2_C_2_/CN had the lowest energy requirement for each step in the photocatalytic water splitting process (Fig. 21e)_,_ thus leading to an enhanced photocatalytic performance of 1.8 mmol g^−1^ h^−1^, much higher than Ag–N/CN.

Very recently, Yu et al. discovered that the single-atom metal–oxygen bonding was favorable for the photocatalytic OER activity, which paved a promising resolution to future efficient water splitting [224]. The single atom metal–oxygen doped polymerized g-C_3_N_4_ (MO-PCN−*x*, where M can be Mo and W, X was annealing temperature) was mainly obtained by the annealing of protonated melon absorbed MO_x_^n−^ ions powder, and its possible calculated structure is also given in Fig. 21f. According to the EPR result, we can see that, in comparison with PCN, the MO-PCN exhibited enhanced signals both in dark and under irradiation, indicating the increased delocalized electronic density and enhanced photoexcitation process, respectively (Fig. 21g). The authors claimed it was the higher Mo–O content than W–O in PCN that induced the stronger EPR signal of Mo-PCN550 than that of W-PCN600. Furthermore, the calculated bandgap of Mo-PCN550 was much smaller than that of PCN (2.3 eV), measuring only 0.54 eV (Fig. 21h-i), indicating the promoted solar harvesting ability of Mo-PCN550, which was also consistent with experimental results. Benefiting from the reduced bandgap and promoted photocarrier transfer and separation of M–O bonding, W-PCN600 exhibited the highest overall water splitting rates of H_2_/O_2_ production rate of 76.9/3.4 μmol h^−1^ m^−2^, significantly surpassing PCN600.

**Fig. 22:**
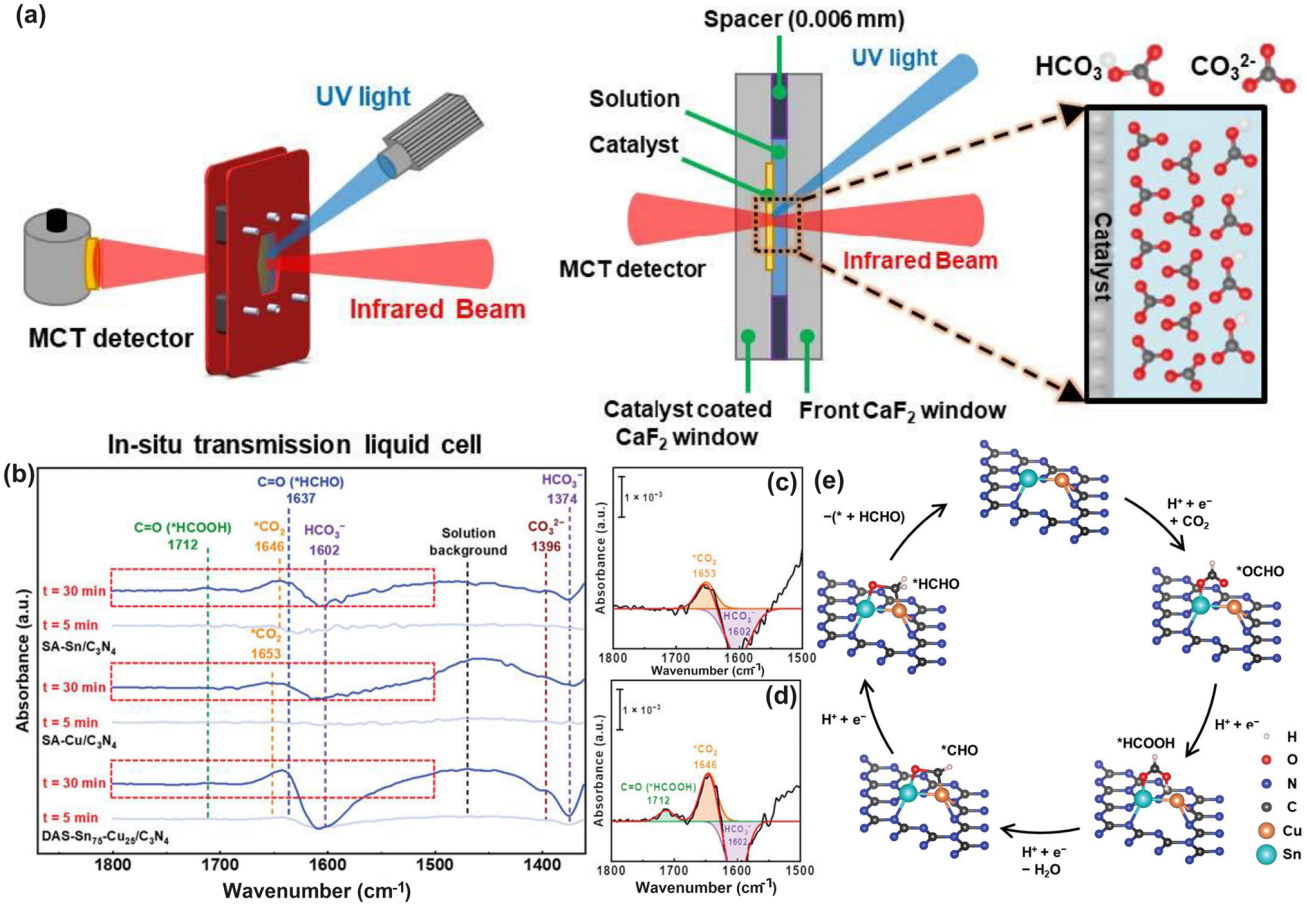
Defect control of bimetallic single atoms doped g-C_3_N_4_. **a** Schematic illustration of setup for in situ photochemical transmission infrared absorption spectroscopy; **b** in situ FTIR spectra of SA-Sn/C_3_N_4_, SA-Cu/C_3_N_4_, and DAS-Sn_75_–Cu_25_/C_3_N_4_ photocatalytic systems at 5 and 30 min in CO_2_-saturated 0.1 m KHCO_3_ and 10 vol% TEA in D_2_O solution. Each spectrum at 0 min was used as a baseline; deconvoluted spectra in the range of 1500–1800 cm^−1^ of **c** SA-Cu/C_3_N_4_ and **d** DAS-Sn_75_–Cu_25_/C_3_N_4_ at 30 min; **e** proposed mechanism of CO_2_ conversion to HCHO over DAS-Sn_75_–Cu_25_/C_3_N_4_ photocatalyst [103]. Copyright 2022, Wiley–VCH

As for the bimetallic single-atom doping, Choi’s group successfully synthesized the single-atom catalyst with dual-atom-sites featuring neighboring Sn(II) and Cu(I) centers embedded in C_3_N_4_ framework (DAS-Sn_x_-Cu_100−x_/C_3_N_4_) by annealing of mixture of urea and Sn– and Cu-acetylacetonate (x was the mass ratio of Sn-acetylacetonate in the mixture of Sn– and Cu-acetylacetonate) [103]. Through the STEM and XANES characterization, the best DAS-Sn_75_-Cu_25_/C_3_N_4_ sample has demonstrated its dual-atom-sites with the coordination environment of M–N_*x*_ bonding without the presence of any M–O or M-M bonding. Importantly, they also employed the in situ transmission FTIR spectroscopy for monitoring the photocatalytic CRR processes, which was similar to that of Fig. 6d-f. Specifically, the reaction cell was sealed with CaF_2_ windows and a spacer, and located in the FT-IR instrument with a mercury cadmium telluride (MCT) as detector (Fig. 22a). The transparent window with a thin layer of g-C_3_N_4_ samples and a solution of 10 vol% TEA in CO_2_-saturated 0.1 m KHCO_3_ well mimicked the CRR process. Interestingly, the in situ FT-IR results indicated the C = O stretching mode at around 1712 cm^−1^ assigned to *HCOOH was found for both DAS-Sn_x_-Cu_100−*x*_/C_3_N_4_ and SA-Sn/C_3_N_4,_ not for SA-Sn/C_3_N_4_ (Fig. 22b-d). Additionally, the most significant peak at 1637 cm^−1^ assigned to the C = O stretch of *HCHO intermediate was only found for DAS-Sn_x_-Cu_100−*x*_/C_3_N_4_, indicating its main product of HCHO, not HCOOH. Furthermore, the NMR test demonstrated the carbon source of HCHO originated from outer CO_2_ rather than g-C_3_N_4_, implying the stability of DAS-Sn_x_-Cu_100−*x*_/C_3_N_4_. Using the triethylamine (TEA) as a proton donor, DAS-Sn_x_-Cu_100−*x*_/C_3_N_4_ first combined with CO_2_ and electron to form *OCHO, then the *HCOOH leaving H_2_O to form *CHO, and finally with proton to become HCHO with the highest produced rate of 259.1 µmol g^−1^ and a selectivity of 61% after 24 h irradiation, far more exceeding its counterparts of single-atom based photocatalysts.

**Fig. 23:**
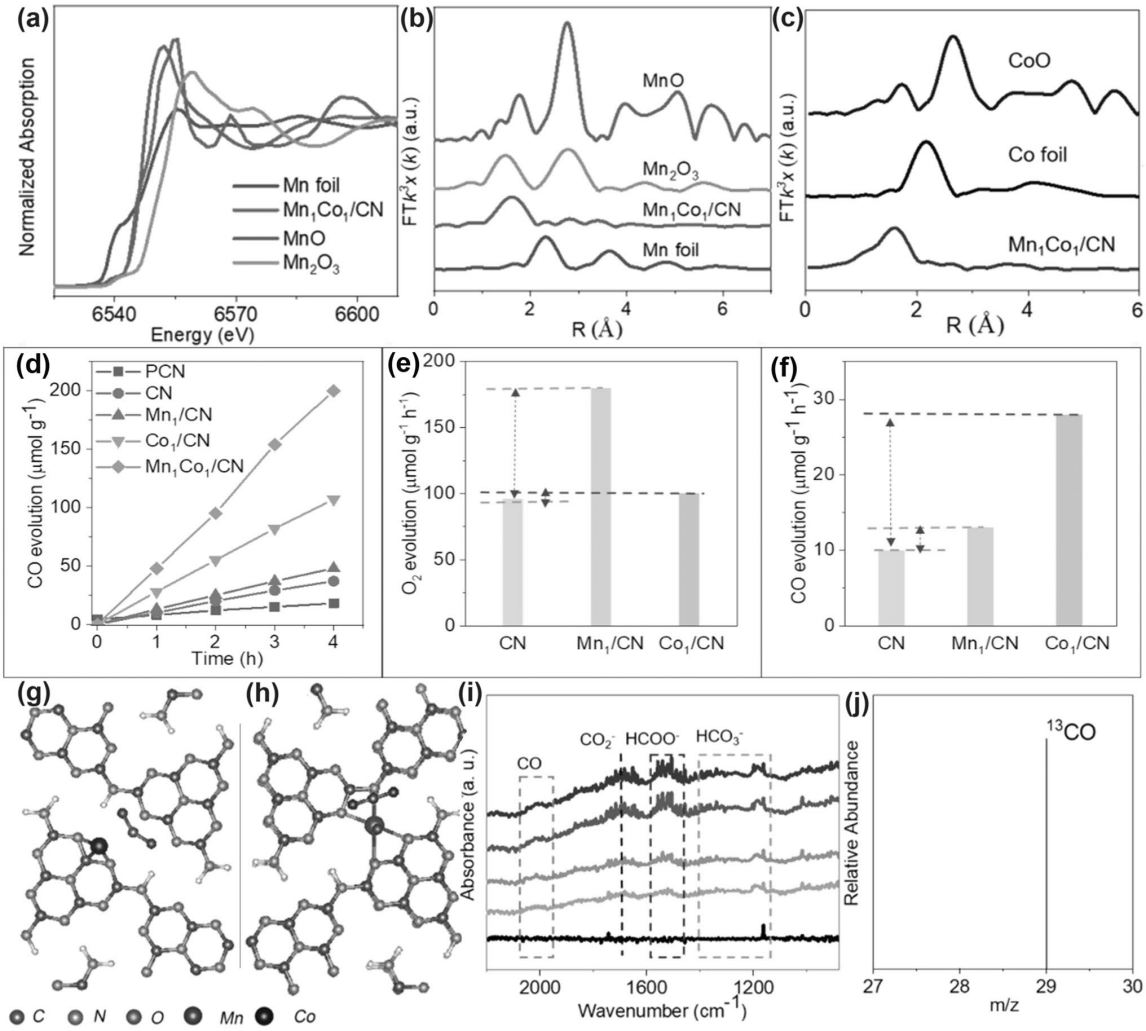
Defect control of bimetallic single atoms doped g-C_3_N_4_. **a** Mn K-edge XANES spectra of the Mn foil, Mn_2_O_3_, MnO and Mn_1_Co_1_/CN; **b** k^3^-weighted Fourier-transform Mn K-edge EXAFS spectra of the samples; **c** k^3^-weighted Fourier transform Co K-edge EXAFS spectra of the samples; Photocatalytic activities of samples with **d** double single-atom active sites, **e** single-atom oxidation sites and **f** single-atom reduction site in CO_2_ reduction; Structure of** h** Co_1_/CNCO_2_ and Mn_1_/CNCO_2_; **i** in situ DRIFTS result of Mn_1_Co_1_/CN for CO_2_ reduction; **j** Mass spectra of ^13^CO driven for Mn_1_Co_1_/CN during light irradiation under ^13^CO_2_ atmosphere [232]. Copyright 2022, Wiley–VCH

In another typical bimetallic single-atom work, Ning and colleagues proposed a Co and Mn SAs co-doped g-C_3_N_4_ (Mn_1_Co_1_/CN) via the atom confinement and supramolecular self-assembly strategy [232]. To determine the forming existence of Co and Mn elements, researchers studied the Mn K-edge XANES spectra and k^3^-weighted Fourier-transform Mn K-edge EXAFS spectra of the samples. As shown in Fig. 23a, the Mn_1_Co_1_/CN peaks located between Mn foil and Mn_2_O_3_, suggesting the valance state of the Mn species lied between 0 and + 3. Figure 23b clearly shows Mn_1_Co_1_/CN had only one main peak located at 1.63 Å without the Mn–Mn bonding, indicating the Mn–N coordination and Mn existed in the form of SAs rather than nanoparticles. This was also true for Co SAs as there was only one Co–N interaction signal located at around 1.73 Å (Fig. 23c). Due to the synergistic effect of Mn and Co SAs, the CO produced rate of Mn_1_Co_1_/CN reached 47 μmol g^−1^ h^−1^ (Fig. 23d). To identify the roles of single atoms in photocatalytic CRR activity, the O_2_ evolution and CO production tests were further performed. It was observed that Mn SAs were beneficial to O_2_ generation (Fig. 23e) leading to the final oxidization of H_2_O_2_ while Co SAs for CO formation (Fig. 23f). According to the DFT calculations, it was evident that CO_2_ molecules were more easily adsorbed near Co atoms in Co_1_/CN as reflected by the bigger bond bending angle and longer bond length in comparison with that of Mn_1_/CN (Fig. 23g-h). This verified that Co SAs were the CO_2_ absorption and activation sites. For the H_2_O oxidization reaction, this was the opposite that Mn SAs were the active sites. According to the in situ DRIFTS measurement, multiple intermediate products such as CO_2_^−^, HCOO^−^, and HCO_3_^−^ were generated, making it easy to form byproducts such as HCOOH and HCO_3_H (Fig. 23i). The combined effect of Co and Mn greatly reduced the production of these byproducts and improved the CRR selectivity. To confirm that the generated CO coming from the reduction of CO_2_ rather than other carbon-containing reactants, the researchers used an isotope of C, ^13^C, to label the C atom (Fig. 23j). The mass spectrometry signal of the obtained product has an m/z value of 29, corresponding to ^13^CO, thus proving that CO was derived from CO_2_ feeding gas. The synergistic effect of Mn and Co SAs indicated the advantage of bimetallic single atoms doping over single-atom doping for photocatalytic CRR performance. In addition, we summarize recent advances in the metallic doped g-C_3_N_4_ for various solar activities in Table 3.

**Table 3:** Recent studies on the metallic-doped g-C_3_N_4_ for photocatalytic performance

Photocatalyst	Element type	Light source	Solar application	Photocatalytic activity	Refs.
Na-CNTs	Na	*λ* > 400 nm	RhB removal	2.47 min^−1^ g^−1^	[233]
Na-CNT_S_	Na	*λ* > 400 nm	RhB removal	2.34 min^−1^ g^−1^	[233]
Cu–TiO_2_/g-C_3_N_4_	Cu	UV–visible light	MB removal	0.22 min^−1^ g^−1^	[234]
K-CN	K	*λ* > 400 nm	HER	1337.2 μmol g^−1^ h^−1^	[95]
CN/K/OH/Fe	Fe/K	*λ* > 420 nm	TC removal	0.547 min^−1^ g^−1^	[235]
NvrCN	Mg	*λ* > 420 nm	No scavenger removal	0.61 min^−1^ g^−1^	[236]
Cu–C–CN	Cu	*λ* > 410 nm	MB removal	0.882 min^−1^ g^−1^	[237]
Fe-g-C_3_N_4_	Fe	*λ* > 420 nm	RhB removal	5.11 min^−1^ g^−1^	[238]
Cu-CNK-OH	Cu/K	*λ* > 410 nm	RhB removal	9.11 min^−1^ g^−1^	[239]
Cu- g-C_3_N_4_	Cu	*λ* > 420 nm	HER	3.02 mmol g^−1^ h^−1^	[240]
NiSCN	Ni/S	*λ* > 420 nm	HER	2021.3 μmol g^−1^ h^−1^	[241]
g-C_3_N_4_/Au/CdS	Au/CdS	*λ* > 420 nm	HER	1060 μmol g^−1^ h^−1^	[242]
NZCC-30	Ni	Visible light	HER	336.08 μmol g^−1^ h^−1^	[14]
K/O@ CN	K/O	Visible light	HER	33.38 μmol g^−1^ h^−1^	[243]
B-ECN	P	Sunlight	TC removal	0.087 min^−1^ g^−1^	[244]
MCNCo5	Co	*λ* > 400 nm	TC removal	15.96 min^−1^ g^−1^	[245]
CdS/CN-30	CdS	*λ* > 400 nm	HER	2120 μmol g^−1^ h^−1^	[246]
Ni_4%_/O_0.2_tCN	Ni/O	*λ* > 420 nm	H_2_O_2_ formation	2460 μmol g^−1^ h^−1^	[247]
GO/FeGCN	Fe	Visible light	RhB removal	0.3 min^−1^ g^−1^	[248]
Mo/Nv-TCN	Mo	*λ* > 420 nm	TC removal	0.98 min^−1^ g^−1^	[249]
PACN	P/O	Simulated sunlight	HER	6437.65 μmol g^−1^ h^−1^	[210]
CN-K	K	*λ* > 420 nm	NO removal	0.078 min^−1^ g^−1^	[212]
K-CN	K	Visible light	RhB removal	0.22 min^−1^ g^−1^	[250]
[WO_4_]^2−^-CN	[WO_4_]^2−^	*λ* > 420 nm	RhB removal	0.22 min^−1^ g^−1^	[251]
Zn/C_3_N_4_	Zn	*λ* > 420 nm	HER	297.5 μmol g^−1^ h^−1^	[252]
Fe (0.5%)/P-CN	Fe	Visible light	Pollutant removal (RhB)	0.0245 min^−1^ g^−1^	[253]
Cu/mpg- C_3_N_4_	Cu	*λ* > 400 nm	MO removal	0.39 min^−1^ g^−1^	[254]
22% KI	K	*λ* > 420 nm	Phenol removal	7.2 min^−1^ g^−1^	[255]
Mo-CN	Mo	Simulated sunlight	CRR	106.44 μmol g^−1^ h^−1^	[256]

## Grafted Functional Groups with Optimized Band Structure

Grafting the organic functional groups onto the g-C_3_N_4_ has also been verified as one of the most promising defect controls for tuning its physicochemical properties with optimized band structure, enhanced solar absorption as well as fast photocarrier transport [257]. Previously reported functional groups included the -C≡N (cyano group), O = C–NH_2_ (urea-like group), -COOH, -C = O, -OH (O-containing groups), and various aromatic rings, of which -C≡N is the most widely investigated one (Table 4).

### Cyano Groups (-C≡N) with Defect States

For example, Zhang and co-workers first proposed a universal alkali hydroxide-assisted preparation of defective g-C_3_N_4_ with abundant -C≡N groups and N vacancies using urea as a precursor [258]. With the increasing content of alkali hydroxide, the resulting g-C_3_N_4_ showed a progressively narrowed bandgap to 2.36 eV with promoted visible-light absorption to 525 nm. A similar phenomenon was also observed for other g-C_3_N_4_ precursors such as melamine, thiourea, and urea. The DFT calculations also confirmed the narrowed bandgap induced by -C≡N modification, while the coexistence of -C≡N and N vacancies would further lead to additional defect energy levels. Besides, a high photocarrier separation efficiency was also achieved by the -C≡N-modified g-C_3_N_4_, rending it with enhanced photocatalytic HER rate up to 6.9 mmol g^−1^ h^−1^.

**Fig. 24:**
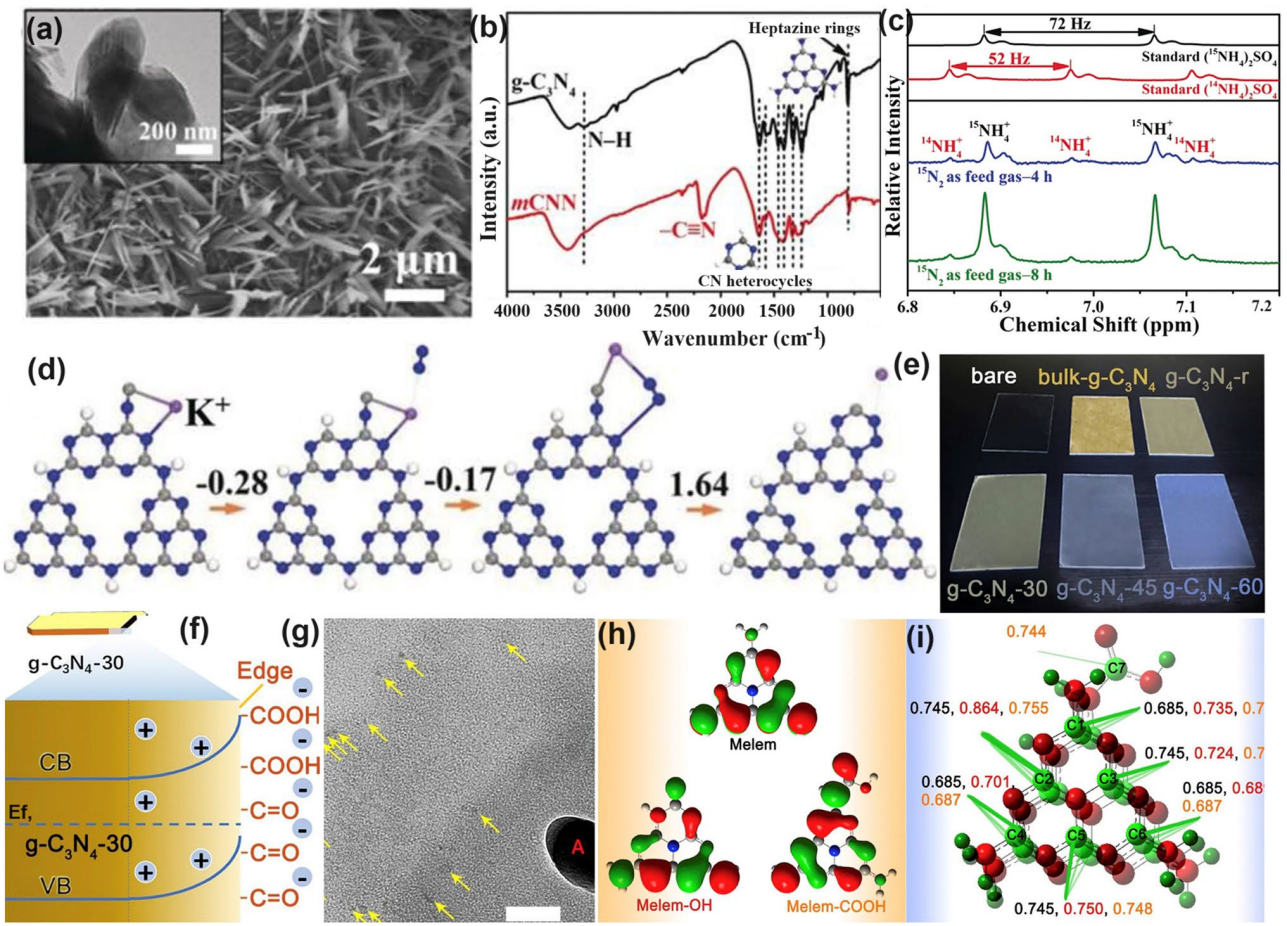
Defect control of cyano groups grafting.** a** SEM and TEM (inset) images of *m*CNN; **b** FT-IR spectra and **c** ^1^H NMR spectra of photocatalytic reaction mediums with ^15^N_2_ as feed gas for different reaction times and standard (^14^NH_4_)_2_SO_4_ and (^15^NH_4_)_2_SO_4_ samples; **d** K^+^-assisted –C/N regeneration process. Blue: N, grey: C, white: H, purple: K^+^ [89]. Copyright 2019, Wiley–VCH. **e** Optical images of bulk g-C_3_N_4_, oxidized g-C_3_N_4_ samples (g-C_3_N_4_−*x*, *x* is the oxidized time), and g-C_3_N_4_-r (reducing g-C_3_N_4_-60 with NH_2_NH_2_·H_2_O); **f** Schematic diagram of possible depletion layer and the band-bending effects near the edge of g-C_3_N_4_-30; **g** TEM image of g-C_3_N_4_-30 after Pt deposition; **h** HOMO of melem^+^, melem-OH^+^, and melem-COOH^+^; **i** Mulliken charge distribution of different carbon atoms in melem^+^ (black), melem-OH^+^ (red), and melem-COOH^+^ (orange) [87]. Copyright 2019, Elsevier

Subsequently, the -C≡N grafted g-C_3_N_4_ nanoribbon (*m*CNN) was developed via the annealing of dicyandiamide and KOH followed by an ultrasonication treatment for photocatalytic NRR (Fig. 24a) [89]. The -C≡N bonding in *m*CNN was confirmed by the typical absorption peak around 2150 cm^−1^ according to the FT-IR spectra (Fig. 24b) and XANES spectra of N K-edge with the broad peak at around 406.3 eV, assigning to the electron transition from N 1* s* to C–N σ*orbital. Compared to pristine g-C_3_N_4_, *m*CNN showed an extended solar light absorption with apparent shoulder peaks at around 450 nm as evidenced by UV–vis DRS. Moreover, the absorption tail of *m*CNN was ascribed to the -C≡N induced subgap states, which extended its solar light absorption range to 700 nm, implying the significantly boosted solar utilization. As a result, the *m*CNN exhibited a promoted NH_3_ yield than pure g-C_3_N_4_ under both N_2_ and Ar atmosphere. A deeper mechanism investigation of the NRR pathway was further explored using an isotope labeling method identified with the ^1^H NMR technique and theoretical calculations. As depicted in Fig. 24c, both ^14^NH_4_^+^ and ^15^NH_4_^+^ were detected during the *m*CNN-based NRR activity, reflecting the ^14^N atoms in g-C_3_N_4_ were involved in the redox reaction. It’s worth mentioning that, with the increasing reaction time, the ratio of the collective integral area of ^15^NH_4_^+^ after 4 and 8 h was 1.65, indicating the continuous generation of ^15^NH_4_^+^ originating from outer ^15^N_2_ feeding gas. However, for ^14^NH_4_^+^, the ratio reduced to 1.07, suggesting the replaced N from *m*CNN was exhausted after a certain time. Considering the active sites of N defects in other systems [113], the authors proposed a possible NRR reaction mechanism that the -C≡N groups that acted as the part of C_2_N_4_ rings to gradually evaluated into NH_3_ with continued regeneration from the outer ^15^N source. Further DFT calculations on NH_3_ generation of *m*CNN confirmed the doped K^+^ was also critical for the stabilization of unsaturated C atoms to form C_2_N_4_ rings and the fixation of N_2_ (Fig. 24d).

### O-containing Groups with Optimized Electron Flow

The O-containing functional groups, such as the -COOH, -C = O, and -OH, have also endowed g-C_3_N_4_ with enhanced solar utilization [259–261]. Wang and colleagues prepared the edge functional g-C_3_N_4_ with -COOH and -C = O groups via an acid oxidization method which was similar to Hummers’ method for graphene exfoliation [87, 262]. With an increasing oxidization etching time, the color of g-C_3_N_4_ turned from yellow to blue, which indicated more O-bearing group species and enlarged bandgaps (Fig. 24e) [87]. Namely, a short time within 30 min rendered g-C_3_N_4_ with -COOH and -C = O groups, which were beneficial to build an optimized electron flow of which a thicker charge depletion layer and band bending were presented when compared to the pristine g-C_3_N_4_ and reduced g-C_3_N_4_-30 sample (Fig. 24f, g). The HOMO diagrams of melem, melem-OH, and melem-COOH imply that the electrons were prone to accumulate in the O atoms with less electron density probability around the neighboring C atoms (Fig. 24h). Consistently, the amount of positive charge on the edge of C atoms from O-containing groups-modified g-C_3_N_4_ was much higher than those away from the edge or those from pristine g-C_3_N_4_, further demonstrating the electrons tend to aggregate on the edge of g-C_3_N_4_-30 nanosheet (Fig. 24i). The authors believed this could accelerate the charge separation and narrow the bandgap, facilitating the generation of H_2_O_2_ for bacteria removal. In contrast, a long oxidization time of 60 min would induce the -OH groups, which was suspected of hindering the in-planar charge transfer and lowering the photocatalytic activity. However, the -OH has been demonstrated to be in good favor of other solar applications according to a previous report [263], which might suggest the complex situation of -OH-modified g-C_3_N_4_ due to the different synthetic methods or different functional groups combination.

### Aromatic Ring Groups with Enhanced Redox Driving Force

**Fig. 25:**
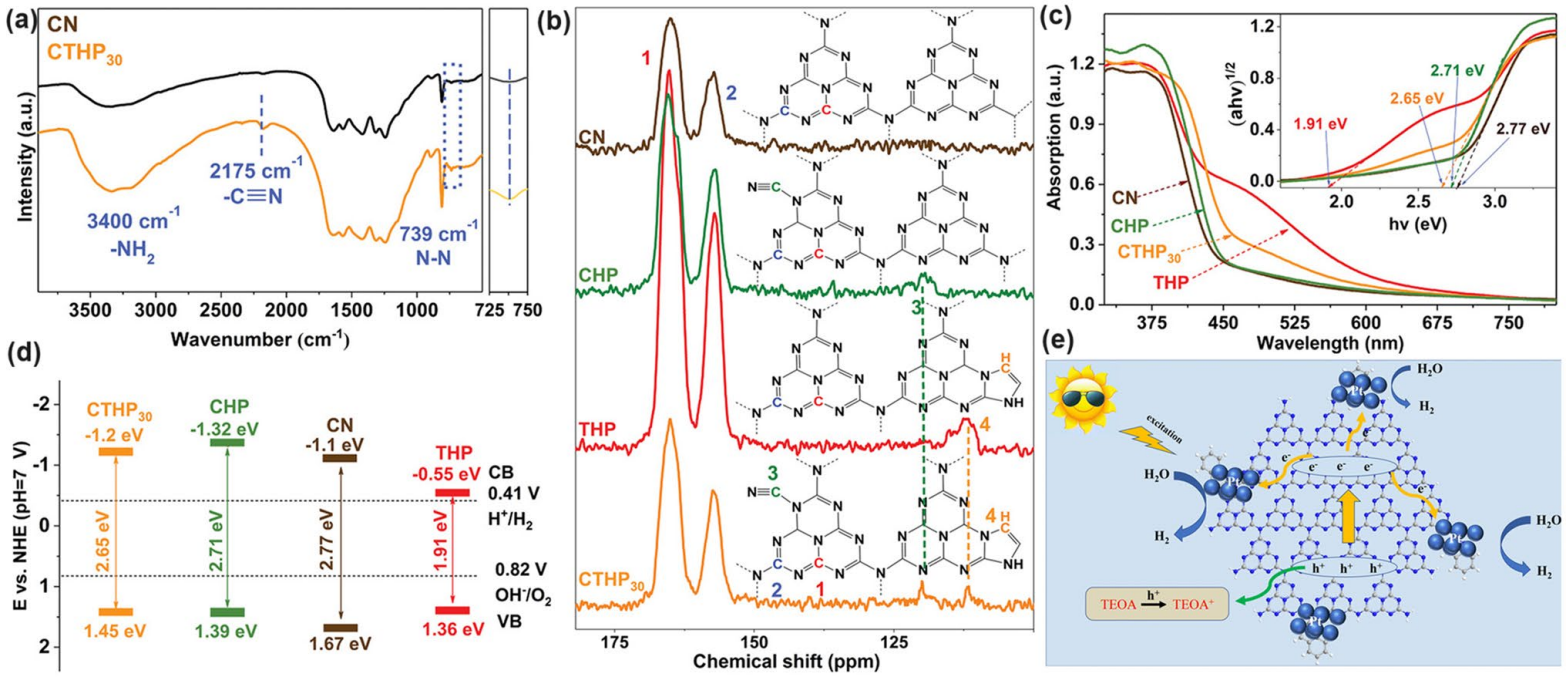
Defect control of aromatic ring groups grafting. **a** FT-IR of CN and CTHP_30_; **b** solid-state ^13^C MAS NMR spectra, **c** UV–vis DRS with Tauc plots (inset), **d** electronic band structure of the pristine CN, CHP, THP, and CTHP_30_ [264]. Copyright 2020, Wiley–VCH. **e** Schematic diagram of the possible photocatalytic reaction mechanism on quinoline ring and naphthalene ring-modified g-C_3_N_4_ under visible-light irradiation photocatalytic HER rates [265]. Copyright 2023, Royal Society of Chemistry

Other aromatic rings, taking triazole groups for instance, have also been investigated according to Wang and co-workers’ work [264]. Annealing the freeze-drying mixture of urea and 3-amino-1,2,4-triazole (3-AT), authors successfully obtained the porous cyanamide-triazole-heptazine polymer (CTHPx, x is the mass ratio of urea to 3-AT) with both triazole groups and -C≡N groups. Controlled samples were pristine g-C_3_N_4_ (CN), triazole groups-modified CN (THP), and cyanamide groups-modified CN (CHP). The corresponding triazole groups and -C≡N groups of CTHP_30_ were identified by the FT-IR spectrums with the typical peaks at around 3400, 2175, and 739 cm^−1^ assigning to –NH_2_, -C≡N, and N–N, respectively (Fig. 25a). More detailed peak affiliation was analyzed from ^13^C cross-polarization magic angle spinning (CPMAS) solid-state NMR spectra (Fig. 25b). According to the UV–vis DRS, we can observe the enhanced solar absorption of these triazole groups- or/and -C≡N-modified g-C_3_N_4_, of which THP without cyano groups displayed the best visible-light absorption, followed by CTHP_30_ (Fig. 25c). However, the CB position of THP was only slightly above the theoretical H_2_ evolution potential, which suggested an inferior driving force that was not beneficial for HER (Fig. 25d). In contrast, the CTHP_30_ exhibited a reasonably narrowed bandgap of 2.65 eV with sufficient driving force for HER reaction, indicating the necessity of multi-functional group modification on g-C_3_N_4_. Therefore, it showed the highest photocatalytic HER rate of 12,723 mmol h^−1^ g^−1^, which was 7.34-fold higher than CN. Similar phenomena were also observed on the quinoline ring and naphthalene ring-modified g-C_3_N_4_ with a five-fold higher HER rate than unmodified g-C_3_N_4_ (Fig. 25e) [265].

Very recently, it is worth mentioning that, similar to g-C_3_N_4_, the covalent organic frameworks (COFs) consisting of organic elements (such as C, N, O, H) via covalent *π* bonding to form a conjunction structure have boosted extensive research interest in the field of photocatalysis [266]. Amine-based COFs, as one of the most important COFs, have suffered from less chemical stability in harsh conditions and insufficient *π* conjugation system with inherent polarization [267]. Fortunately, similar to the boost photocatalytic activity induced by grafted function groups in g-C_3_N_4_ matrix, the poor photocatalytic performance can also be well improved by the substitution of linkages by introducing the azole linkages [268]. Furthermore, the grafted β-ketoenamine to imine moieties in the linkages was also efficient in generating a non-quenched excited state and a more favorable HOMO level, thus leading to an enhanced photocatalytic HER rate [269]. It is the most exciting study that Wang’s group has demonstrated that the triazine-containing COFs framework could significantly boost the H_2_O_2_ photosynthesis rate up to 2111 μM h^−1^ due to the high-speed photocarrier transfer pathway of dual donor–acceptor structure [270], which provided us a new thinking of the combination of g-C_3_N_4_ with functional COFs linkages groups.

## Crystallinity with Extended Conjugation System

As the concrete manifestation of broader and weakened (002) and (001) planes, the crystallinity of most defect-engineered g-C_3_N_4_ would reduce due to the disrupted periodicity induced by the internal vacancies or the external impurities during the intensive thermal annealing/etching process [104, 117, 271]. Accompanying with enhanced surface area, unique nanostructure, and defect formation, their overall solar activity is generally enhanced [171]. Being on the opposite side of defect engineering toward g-C_3_N_4_, a higher crystallinity normally indicates a more regular atomic arrangement with extended and fully condensed conjugation structure, which stabilizes the *π*-electron system for fast charge mobility and improves the solar utilization due to the reduced bandgap and less photocarrier traps induced by defects. Therefore, it would be fancy if paying great attention to see if there is a balance between crystallinity and defect. To this end, this section will begin with the ideal crystalline types of g-C_3_N_4_ including the poly-heptazine imides (PHI) type and poly-triazine imides (PTI) type, followed by giving typical samples of the combination of defect creation in high crystalline g-C_3_N_4_ toward enhanced photocatalytic activity [83, 88, 106, 272, 273]. The details were extended as follows:

### Poly-heptazine Imides (PHI) with Weakened Interlayered van der Waals Interaction

**Fig. 26:**
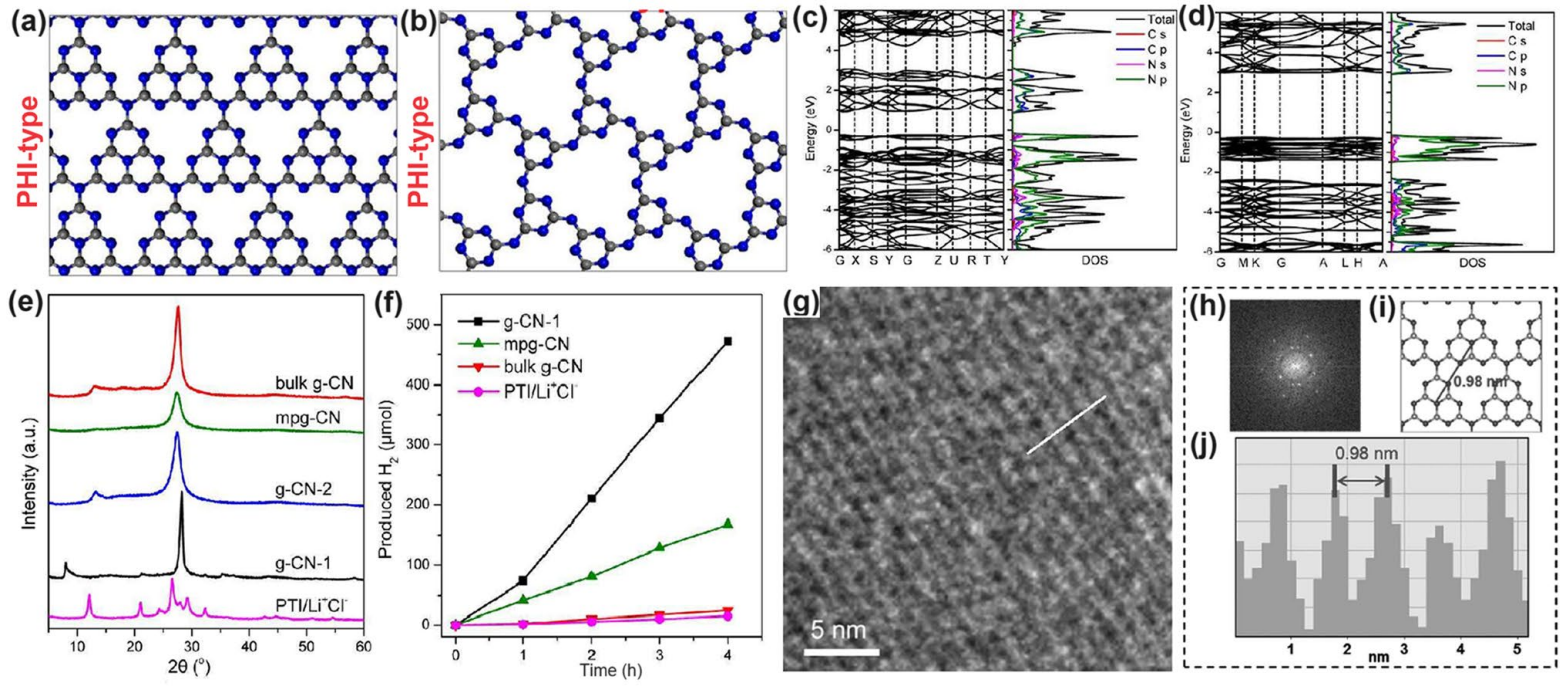
Defect control of crystallinity improvement (PHI type). Ideal crystalline g-C_3_N_4_ in **a** PHI-type and** b** PTI-type; DOSs of **c** PHI-CN and **d** PTI-CN;** e** XRD patterns and **f** photocatalytic HER performance of various g-C_3_N_4_ samples with different crystallinity [273]. Copyright 2016, American Chemical Society. **g** HRTEM images of PHI-CN nanosheet and **h** its corresponding FFT patterns;** i** crystalline structure; **j** thickness [83]. Copyright 2017, Wiley–VCH

The PHI-typed g-C_3_N_4_ (PHI-CN) is the most widely investigated model for the current research study, which is an infinite repeat of the tri-s-triazine unit (Fig. 26a). While PTI-typed g-C_3_N_4_ (PTI-CN) is composed of triazine unit connected with N atoms in the bridging site (Fig. 26b). Generally speaking, PHI-CN is normally synthesized via a simple ion-thermal strategy using bulk g-C_3_N_4_ and MCl (M = Li, Na, K) as precursors [83]. Compared to PTI-typed g-C_3_N_4_ (PTI-CN), most PHI-typed g-C_3_N_4_ (PHI-CN) materials have demonstrated a significant improvement in solar activity. In a pioneered work, Lin and co-workers found that the PHI-CN had a smaller calculated bandgap than PTI-CN (1.17 vs. 3.23 eV), indicating its superior visible-light response ability due to the extended conjugated *π* system (Fig. 26c–d) [273]. Additionally, the intercalated Li^+^ and Cl^−^ in PTI-CN have little influence on narrowing the band compositions. Regarding XRD patterns, the PHI-CN assigned to g-CN-1 exhibited much higher crystallinity with sharper (002) and (001) peaks moving in the opposite direction when compared to its bulk counterparts (bulk g-CN, mpg-CN, and g-CN-2, Fig. 26e). This was supposed to be the enhanced polymerization degree with fewer hydrogen bonds and strong chemical interaction between interlayers. While the PTI-CN assigned to PTI/Li^+^Cl^−^ showed a distinct XRD pattern, which was also consistent with the previous report [81, 88, 274–276]. Due to the structure and crystallinity difference, g-CN-1 displayed the highest photocatalytic HER rate of 770 μmol h^−1^, far more exceeding PTI/Li^+^Cl^−^, which indicated the advantage of PHI units over PTI motifs (Fig. 26f). A subsequent study was performed to unravel the relationship between HER performance and PHI nanostructure, which employed the ultra-thin PHI-CN nanosheets as the target material [83]. Its few-layered merit was reflected by the obvious (002) lattice fringes according to the HRTEM image and a layer thickness at around 0.98 nm (Fig. 26g–j). Compared to the bulk PHI-CN, PHI-CN nanosheets exhibited a 2.5-time higher HER rate under visible-light irradiation, further confirming that nanostructure engineering is also important for solar applications.

**Fig. 27:**
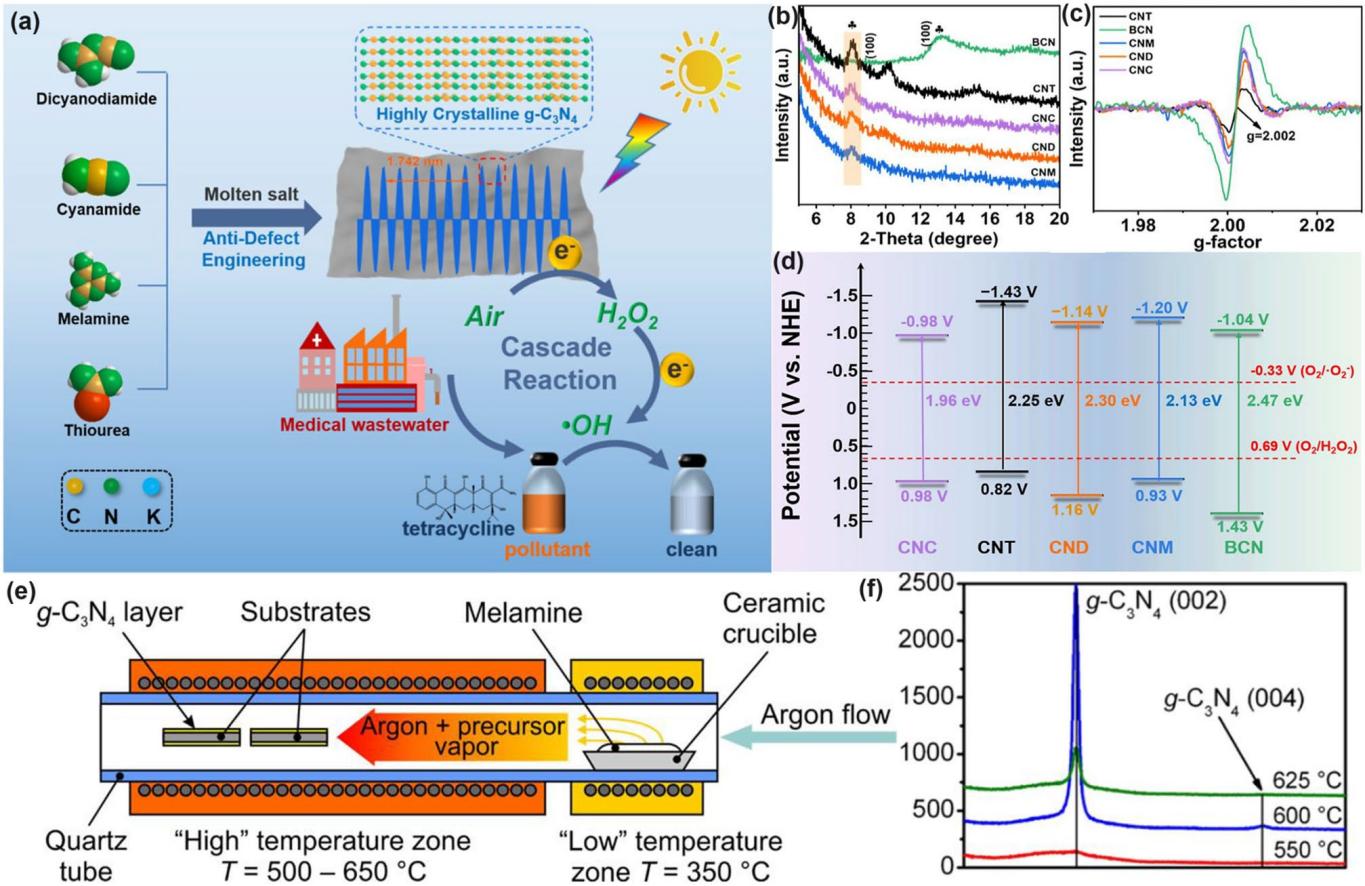
Defect control of crystallinity improvement (PHI type). **a** Schematic diagram of preparing crystalline g-C_3_N_4_ using different precursors for in situ reduction of H_2_O_2_ and wastewater purification; **b** HRTEM image and **c** ESR spectrum of CNT; **d** electronic band structures of BCN, CNC, CND, CNM, and CNT [277]. Copyright 2023, American Chemical Society. **e** Schematic diagram of open tube dual-zone furnace for preparing crystalline g-C_3_N_4_ films; **f** XRD patterns of crystalline g-C_3_N_4_ films with annealing temperatures [279]. Copyright 2022, American Chemical Society

Similar research has also been carried out by employing the ion-thermal strategy to prepare the high crystalline g-C_3_N_4_ [277]. As shown in Fig. 27a, various precursors including cyanamide, dicyandiamide, melamine, and thiourea were annealed in the presence of eutectic NaCl/KCl salts, and the corresponding g-C_3_N_4_ samples were donated as CND, CNC, CNM, and CNT, respectively. According to the HRTEM, one can see the obvious lattice stripes assigned to the (202) crystal plane of CNT, indicating its good crystallinity (Fig. 27b). Besides, compared to the bulk g-C_3_N_4_, their XRD patterns revealed a new peak at around 8.0°, ascribing to a large interplanar packing distance of 1.104 nm due to the molten salt condition during the pyrolysis [278]. Interestingly, the (002) peaks were witnessed with a positive shift, which evidenced the reduced interlayered distance. This was due to the induced high crystallinity that substantially suppressed the edge amino groups, weakening the interlayered van der Waals force and lowering the defect density, and thus the photocarrier separation efficiency would be boosted. This was in good accordance with the reduced EPR signal intensity of CND, CNC, CNM, and CNT at *g* = 0.002, attributing to dangling bonds or nitrogen defects (Fig. 27c). After the analysis of Mott–Schottky plots and DRS results, the authors gave their detailed electronic band structures as displayed in Fig. 27d. In contrast to the low crystalline bulk g-C_3_N_4_, all these crystalline g-C_3_N_4_ exhibited a narrowed bandgap by 0.22–0.51 eV, indicating an enhanced visible-light responsive ability. Accordingly, CNT had the most negative CB value of − 1.43 V, which was a sign for the best candidate for the generation of ·O_2_^−^ radical. However, its driving force for H_2_O_2_ generation was the lowest as its most lifted VBM at around 0.82 V. Fortunately, CNT displayed the maximum H_2_O_2_ production of 2.48 mmol g^−1^ h^−1^ with an apparent quantum efficiency of 22% (*λ* = 400 nm) among all samples, further suggesting the superior role of high crystallinity over the corresponding oxidization ability.

Different from the ion-thermal-assisted polymerization of PHT-CN, Chubenko et al. adopted a chemical vapor deposition (CVD) method to prepare the crystallized g-C_3_N_4_ thin film up to 1.2 μm on the glass and silicon substrate [279]. As shown in Fig. 27e, the CVD furnace was divided into the former lower-temperature zone below 350 °C and later high-temperature zone in the range of 500–650 °C. The later zone was heated to the target temperature with the substrates inside followed by the continued heating of a low-temperature zone containing melamine powder to provide the precursor atmosphere at 350 °C in dry Ar gas. Moreover, the crystallinity of g-C_3_N_4_ reached the highest level when the target heating temperature was 600 °C even using the tiny amount sample on the glass substrate as reflected by the strong (002) diffraction peak and obvious (004) peak signal as shown in Fig. 27f. Exhibiting the best crystallinity, g-C_3_N_4_ synthesized under 600 °C also owns a moderate bandgap of 2.87 eV, which was 0.16 eV smaller than that obtained under 550 °C, still showing potential for future photocatalytic activity.

It is worth mentioning that the crystalline g-C_3_N_4_ obtained via the ion thermal reaction using the metal salts as the solvent cannot avoid all surface defects, particularly the insertion of K atoms accompanying edge cyano groups. Although some research works aimed to achieve the anti-defect engineering goal, this is almost not possible to realize this. In most cases, they were enhanced crystalline g-C_3_N_4_ with tiny defects. Therefore, it provided us with an opportunity to optimize the photocatalytic activity of g-C_3_N_4_ by balancing the crystallinity and defect types. The following section focuses on the combination of crystallinity and defects toward enhanced photocatalytic activity.

**Fig. 28:**
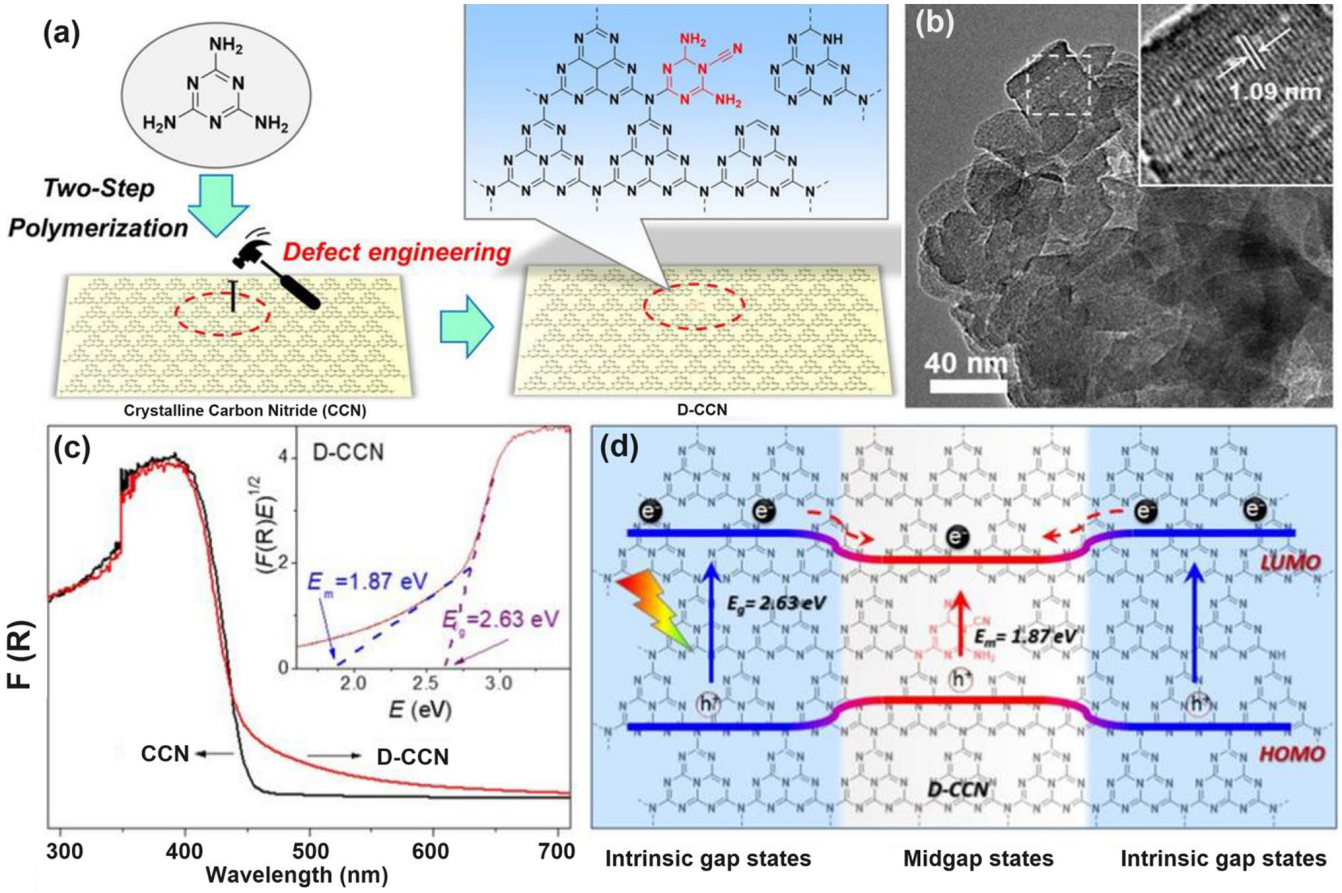
Defect control of crystallinity improvement (PHI type) with functional groups.** a** Illustration of fabrication strategy combining crystallinity and defect control; **b** HRTEM image of D-CCN; **c** DRS spectra of CCN and D-CCN, bandgap of D-CCN determined by Tauc plots (inset); **d** schematic illustration of the speculative band structure in D-CCN [106]. Copyright 2019, Wiley–VCH

In continuing work, a strategy combining high crystallinity and N defects was proposed to boost the photocatalytic HER of PHI-CN [106]. Herein, the resulting defective sample (D-CCN) with cyano group and the unpolymerized amino group was obtained after annealing the mixture of high crystalline g-C_3_N_4_ (CCN) and NaBH_4_ in N_2_ (Fig. 28a). Although the impurities have been imported into the D-CCN, they still preserved high crystallinity as evidenced by the clear lattice fringes of 1.09 nm assigned to the* d* spacing of in-plane (001) layers (Fig. 28b). Intriguingly, the high crystallinity of D-CCN could reduce the unblocked channels across the 2D conjugated *π* in-planes, which enabled smooth in-plane charge transport and easier excitation dissociation. While the decreased (002) spacing further shortened the lateral distance, it enhanced the charge transfer along the vertical direction. Additionally, the defects induced by the mid gaps could extend the visible-light absorption to 610 nm and promote the charge separation efficiency (Fig. 28c). The authors claimed that the defect-modified D-CCN might be composed of two parts: (1) main framework with PHI-CN exhibiting an intrinsic bandgap of 2.63 eV and (2) partial matrix of functional groups grafted defective g-C_3_N_4_ with a midgap of 1.87 eV. Due to the energy difference between these gaps, the electron flow could be expected as shown in Fig. 28d, giving rise to the significantly promoted photocarrier separation efficiency. Therefore, we observed a substantial improvement of photocatalytic HER performance for D-CCN to 64 μmol h^−1^, which was 8 and 40 times higher than those of CCN and bulk g-C_3_N_4_ with low crystallinity, respectively.

**Fig. 29:**
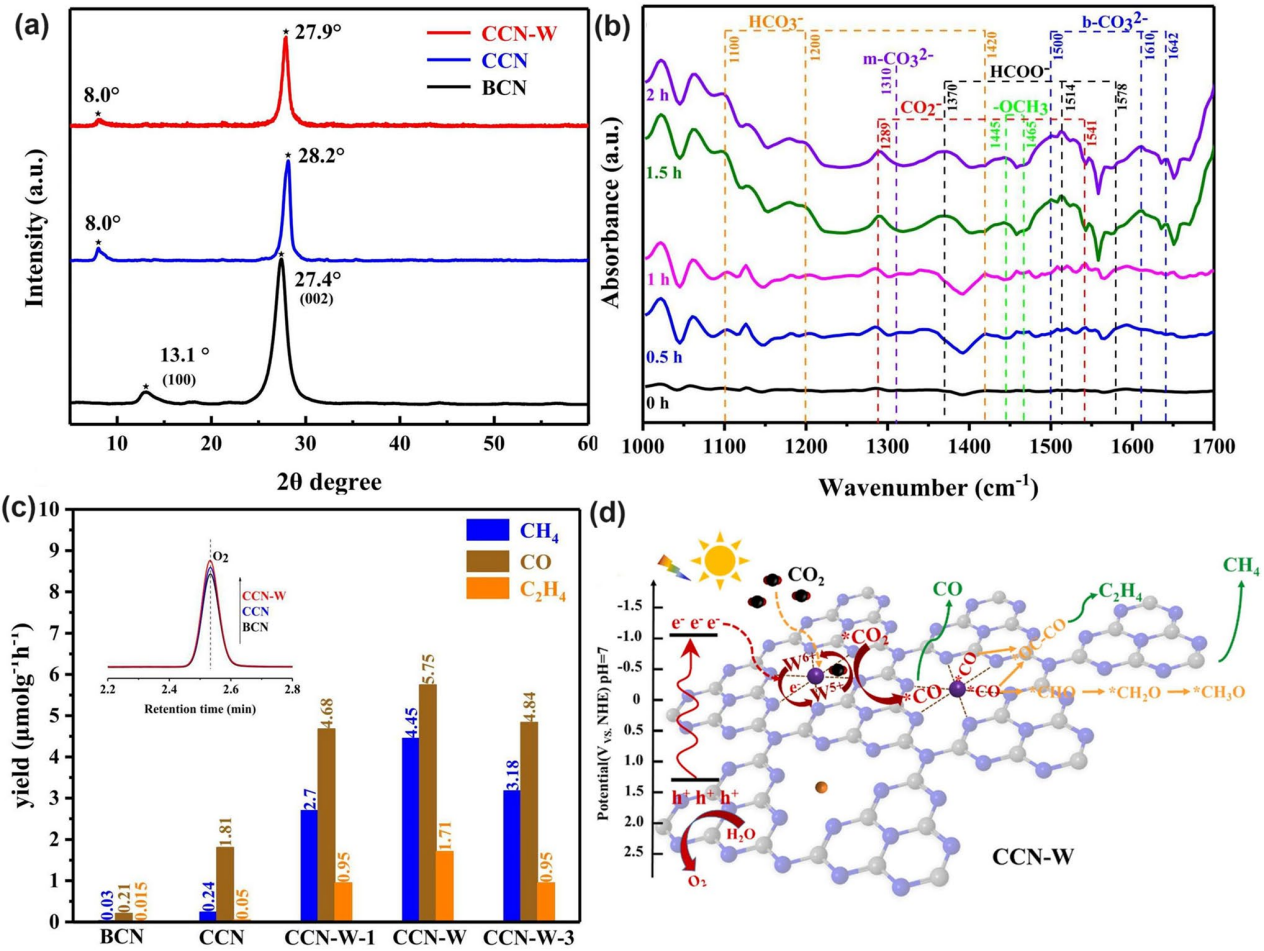
Defect control of crystallinity improvement (PHI type) with metallic doping. **a** XRD patterns of BCN, CCN, and CCN-W; **b** In situ DRIFTS of photocatalytic CRR intermediates in the absence (0–1 h) and presence (1–2 h) of LED illumination for CCN-W; **c** photocatalytic CRR products of various g-C_3_N_4_ samples under full spectral irradiation; **d** diagrammatic of multifunctional role of W-N_6_ active centers for photocatalytic CRR over CCN-W [280]. Copyright 2022, Elsevier

Liang et al. contributed outstanding research on combining the crystalline g-C_3_N_4_ with W-doping in the cell void sites of PHI units via a solvothermal reaction employing the crystalline g-C_3_N_4_ (CCN) and W(CO)_6_ as starting materials [280]. As we know, bulk g-C_3_N_4_ (BCN) obtained from the traditional pyrolysis method suffered from an overloading of edge amino groups, which dramatically destroyed its crystallinity, worsening the photocarrier transfer pathway along the in-plane direction. In contrast, CCN was observed with fewer surface defects such as K atoms in the void and few cyano groups on the edge, which was demonstrated to narrow its bandgap and boost the photocarrier transfer process. According to the SEM and HRTEM mapping result, the W elements were distributed evenly without obvious nanoclusters aggregations. Along with the large W atomic radius, the W-doping position should be similar sites with that of K atoms in the void position. It was clear that the crystallinity of W-doped crystalline g-C_3_N_4_ (CCN-W) did not change with a d-spacing of 0.98 nm assigned to the interlayered (100) planes, which was similar to that of CCN. Interestingly, an obvious XRD diffraction peak at around 8° was witnessed for both CCN and W-CCN, which were supposed to be the (100) planes due to the K-doping (Fig. 29a). Also, there was a slight (002) peak shifting from 28.2° to 27.9° of CCN over CCN-W, suggesting a larger interlayered distance of CCN-W owing to the bigger W atomic radius. Remarkably, the EPR signal of CCN-W was weaker and stronger than that of BCN and CCN, respectively. This indicated that W^6+^-doping was beneficial to balance the N vacancy concentration and crystallinity. When it comes to the photocatalytic CRR activity, the dominant species were CO^2−^, HCO_3_^−^, and m-CO_3_^2−^ before irradiation for CCN-W as reflected by the in situ DRIFTS spectra (Fig. 29b). The in situ FT-IR spectroscopy measurement recorded within 2 h of photocatalytic CRR activity further revealed the formation of bicarbonate b-HCO_3_^2−^, HCOO^−^ and –OCH_3_ groups with their peaks located at 1100/1200/1420, 1370/1514/1578, and 1450 cm^−1^, respectively. As a result, CCN-W delivered the highest CO, CH_4_, and C_2_H_4_ yields of 5.75, 4.45, and 1.17 μmol g^−1^ h^−1^, respectively (Fig. 29c). Notably, the introduction of active W-N_6_ sites into CCN-W not only enhanced and activated the adsorption capacity for CO_2_ and CO with a moderate affinity ability but also enriched photoelectrons, which was critically beneficial for the high collision possibility and low CRR barrier, thus leading to the main production of hydrocarbons with a high selectivity of 83%. In contrast, CCN displayed a lower CRR yield in comparison with the W-doped sample with a high selectivity of CO, further demonstrating the superior role of W-N_6_ in boosting the photocatalytic activity of crystalline g-C_3_N_4_ (Fig. 29d). Thus, these pieces of work provide us with a new pathway balancing both crystallinity and defect creation, which is of vital importance for the future design of g-C_3_N_4_-based photocatalysts for solar applications.

### Poly-triazine Imides (PTI) with Active {1010} Facets

**Fig. 30:**
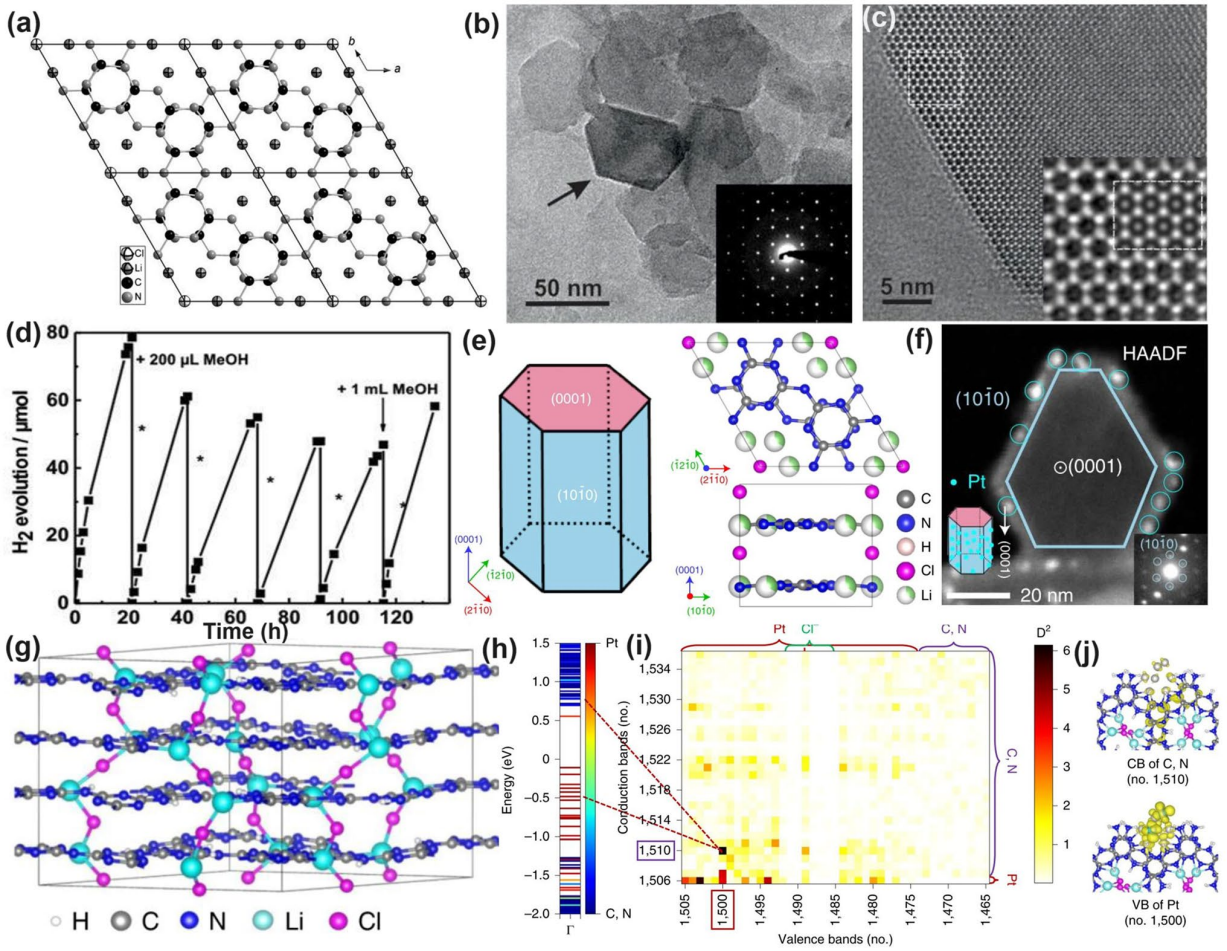
Defect control of crystallinity improvement (PTI type). **a** Parallel projection of PTI/Li^+^Cl^−^ structure [272]; Copyright 2011, Wiley–VCH.** b** TEM image of exfoliated ultrathin PTI/Li^+^Cl^−^ nanosheets; **c** HRTEM image of PTI/Li^+^Cl^−^ nanosheets viewed along (001) direction; **d** photocatalytic HER activity of PTI/Li^+^Cl^−^ [81]; Copyright 2014, American Chemical Society.** e** Left, schematic illustration of the PTI/Li^+^Cl^−^ single crystal with the basal plane terminated by two {0001} planes and the side faces terminated by six equivalent {1010} facets. Right, the crystal structure of PTI/Li^+^Cl^−^; **f** HAADF-STEM image of a PTI crystal aligned close to the {0001} direction with Pt nanoparticles on prismatic {1010} facets; **g** computational PTI/Li^+^Cl^−^ model; **h** projected band structure (Г-point, spin up) of Pt_8_@PTI/Li^+^Cl^−^; **i** transition dipole moments between VBs and CBs and **j** charge density of Pt_8_@PTI/Li^+^Cl^−^ with band numbers of 1,510 (CB of C, N) and 1,500 (VB of Pt) [276]. Copyright 2020, Springer Nature

Lotsch’s group first proposed the ion thermal method to obtain PTI-based carbon nitrides, which employed the eutectic mixture of LiCl and KCl as solvent [272]. The synthetic route concluded two steps: (1) Pre-heating of dicyandiamide and molten salt under an inert Ar atmosphere at 400–500 °C; (2) Annealing above mixture under vacuum for a long time up to 48 h, the brownish PTI/Li^+^Cl^−^ was obtained. Based on the XRD, TEM, and solid-state NMR spectroscopy analysis, PTI/Li^+^Cl^−^ was found to exhibit a high crystallinity with ABA stacking and separated by the weak van der Waals forces. Additionally, the Li^+^ and Cl^−^ were located in the channels along the *Z*-axis direction (Fig. 30a). Further investigation on the exfoliated ultra-thin PTI nanosheets showed that the hexagonal shape and triazine unit were kept as reflected by the HRTEM images in Fig. 30b–c [81]. Interestingly, the resultant PTI nanosheets enabled the H_2_ evolution under solar irradiation (Fig. 30d). However, its cycling HER activity in the TEOA solution suffered from severe performance decay, indicating instability in the basic environment. While changing to a methanol additive, the HER performance showed reasonable stability within 130 h, suggesting the promising potential in photocatalysis.

Further identification of the reactive planes of PTI/Li^+^Cl^−^ during the photocatalytic HER has been performed by fabricating PTI/Li^+^Cl^−^ single crystals via a modified above-mentioned synthetic method with a higher rating rate of 6 °C min^−1^ and a shorter post-annealing time of 12 h [276]. The corresponding PTI/Li^+^Cl^−^ sample showed a regular hexagonal structure with prismatic {1010} planes and basal {0001} planes as shown in Fig. 30e. According to previous reports, the poor solar water splitting performance of PTI-based g-C_3_N_4_ can be enhanced by the photo-deposition of suitable co-catalysts such as Pt and Co clusters. The HAADF-STEM image of PTI/Li^+^Cl^−^ displayed that the Pt clusters were deposited on the prismatic {1010} facets with negligible distribution on {0001} planes (Fig. 30f). In addition, contrast samples PTI−*x* with different S_{1010}_/S_{0001}_ ratios were also obtained, where x is the annealing temperature. The higher S_{1010}_/S_{0001}_, the better overall water splitting performance is. Correspondingly, PTI-550, with the highest ratio, exhibited the best HER and OER rates of 189 and 91 μmol h^−1^, respectively. These results demonstrated the prismatic {1010} planes were the active facets, which was also confirmed by the transition dipole moments between the CB and VB using the Pt_8_ cluster absorbed on the {1010} planes of PTI/Li^+^Cl^−^ as a calculated model (Fig. 30g–j). The photogenerated electrons were demonstrated to migrate from the Pt_8_ energy levels (band no. 1475–1505) to CB of g-C_3_N_4_ (above band no. 1510) on the active {1010} facets. Besides, recent advancements of crystalline g-C_3_N_4_ in both PHI and PTI types are summarized in Table 4.

**Table 4:** Recent advancement of defective g-C_3_N_4_ with functional groups and highly crystalline g-C_3_N_4_ for different solar applications

Photocatalyst	Light source	Solar application	Photocatalytic activity	Refs.
PTI/Li^+^Cl^−^	*λ* > 300 nm	HER	1890 μmol g^−1^ h^−1^	[276]
CGCN	*λ* > 100 nm	HER	600 μmol g^−1^ h^−1^	[281]
p-gCN-NS	*λ* > 400 nm	RhB removal	0.97 min^−1^ g^−1^	[282]
CNCN	*λ* > 420 nm	HER	3591 μmol g^−1^ h^−1^	[129]
NPZ0.5	*λ* > 420 nm	HER	1360 μmol g^−1^ h^−1^	[283]
C-Ti_2_NBs/g-C_3_N_4_/Fe_3_O_4_	Visible light	MO removal	0.16 min^−1^ g^−1^	[284]
CCNNS_S_	*λ* > 420 nm	HER	1060 μmol g^−1^ h^−1^	[83]
g-CN-1	*λ* > 420 nm	HER	15,400 μmol g^−1^ h^−1^	[273]
BaCN-C_3_N_4_	*λ* > 420 nm	HER	7382 μmol g^−1^ h^−1^	[89]
COC30	Visible light	HER	1336.8 μmol g^−1^ h^−1^	[285]
PYM100-CN	*λ* > 420 nm	HER	5.418 μmol g^−1^ h^−1^	[286]
O–g-C_3_N_4_	simulated sunlight	HER	7285 μmol g^−1^ h^−1^	[175]
K/S@CN	Visible light	CRR	16.27 μmol g^−1^ h^−1^	[287]
CNS-500	*λ* > 420 nm	H_2_O_2_ formation	4980 μmol g^−1^ h^−1^	[288]
PCN-G	*λ* > 400 nm	HER	5.5 mmol g^−1^ h^−1^	[289]
CCN-W	420–780 nm	CRR	11.91 μmol g^−1^ h^−1^	[280]
DCN350	*λ* > 420 nm	HER	1541.6 μmol g^−1^ h^−1^	[290]
CNT	*λ* > 400 nm	H_2_O_2_ formation	2.48 mmol g^−1^ h^−1^	[277]
OPCN	*λ* > 400 nm	H_2_O_2_ formation	1002.4 mmol g^−1^ h^−1^	[199]
g-CN-I	*λ* > 420 nm	HER	5880 μmol g^−1^ h^−1^	[291]
GOD-OCN-3d	*λ* > 420 nm	Cr^4+^ removal	0.78 min^−1^ g^−1^	[58]
g-C_3_N_4_-0.01	*λ* > 420 nm	HER	0.44 mmol g^−1^ h^−1^	[258]
DCN-200	*λ* > 420 nm	HER	4020 μmol g^−1^ h^−1^	[292]
CN680	*λ* > 440 nm	HER	310 μmol g^−1^ h^−1^	[293]
CNHP_30_	*λ* > 420 nm	HER	12,723 μmol g^−1^ h^−1^	[264]
g-C_3_N_4_-30	*λ* > 400 nm	Photocatalytic disinfection	30.7 min^−1^ g^−1^	[87]
20OTh5/g-C_3_N_4_	Visible light	HER	3.63 mmol g^−1^ h^−1^	[294]
DMC30	*λ* > 420 nm	HER	306.52 μmol g^−1^ h^−1^	[295]

### Recent Discussion on Defect Traps with fs-TAS

Based on the above discussion, defect engineering has demonstrated an indispensable contribution to improving the solar utilization of g-C_3_N_4_ toward various solar applications. With discreet regulations, mono/multiple types of defects (vacancies, dopants, and functional groups) can induce additional impurity energy levels, such as midgap states and subgap states in g-C_3_N_4_ [102, 110, 171]. Most cases emphasize the critical role of these defect states: (1) lowering the photocarrier excitation energy with extended visible-light absorption; (2) acting the temporary electron reservoir to accept the migrated electrons from CB, further inhibiting the photocarrier recombination rate. However, the side effects of these defect states in g-C_3_N_4_ have drawn less research attention compared to those in other semiconductor-based systems, which mainly include: (1) deeper defect energy levels with insufficient redox driving force to restrain the formation of desired products; (2) trapped energy levels or detrimental surface states acting as photocarrier recombination centers that reduce the amount of thermodynamically satisfied electrons.

**Fig. 31:**
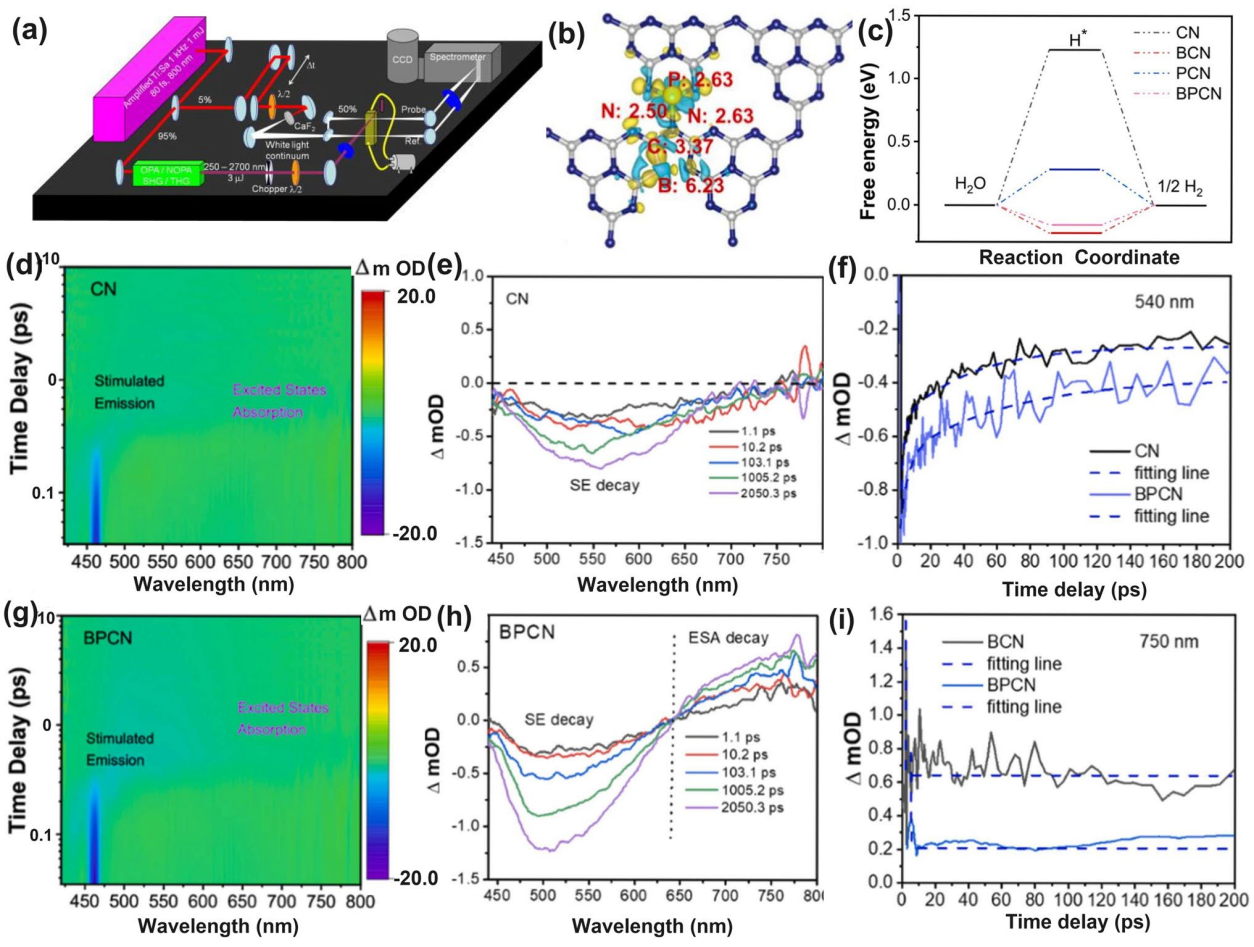
Defect control of defect states. **a** Schematic representation of a fs-TASM setup [309]. Copyright 2012, Elsevier. **b** Charge density difference maps of the as-obtained sample (yellow and cyan regions represented the electron accumulation and depletion; values at the atoms were the Bader charge); **c** free energy profiles of hydrogen absorption over the CN, PCN, BCN and BPCN for HER; time-dependent contour plots of fs-TAS (pump laser: 400 nm): **d** CN, **g** BPCN; TAS of **e** CN, **h** BPCN; Kinetics decay profiles of **f** CN and BPCN at 540 nm and **i** BCN and BPCN at 750 nm [112]. Copyright 2024, Elsevier

Despite few studies on g-C_3_N_4_ deep into this concern, there are tremendous research experiences in other photocatalyst systems to be referred to [296–307]. For example, the dangling bonds aroused during the defect manipulation might cause deteriorated surface states which would further lower the solar activity. The surface states have also been well studied in the application of photoelectrochemical water splitting. For instance, Benjamin and co-workers proposed an electrochemical EIS method to interpret the effect of surface states in the charge transfer process [305]. These surface states were optimized with the accumulation of holes at the α-Fe_2_O_3_/electrolyte interface, which is beneficial for water oxidation. Furthermore, theoretical calculations verified the N-doping level is vital for the position of defect states in La_2_Ti_2_O_7_ [304]. Specifically, one N atom replacing the O atoms would lead to deep localized states, which was not good for photocatalytic activity. Two N atoms and one O vacancy induced a continuum energy band just below the CB of La_2_Ti_2_O_7_, which enabled a fast charge transfer rate and enhanced solar utilization. This finding allows us to develop advanced defect control with optimized defect states. Recently, several studies on identify the shallow defect states to explore the deep photocarrier transfer kinetics have emerged using the femtosecond transient absorption spectrometer (fs-TASM) [112, 144, 308]Typically, the time and space-resolved fs-TASM is composed of a femtosecond Ti/Sapphire regenerative amplifier laser system to generate a pulse of tens femtosecond and a data acquisition transient absorption spectrometer. After the amplifier and BBO crystals, the laser with a certain wavelength can be obtained as the pump, and the probe pulse can also generate a white-light continuum spectrum. The pump and probe beams are focused onto the sample to get the temporal and spatial overlap (Fig. 31a) [309]. Very recently, Gao et al. fabricated the B and P co-doped g-C_3_N_4_ (BPCN) and found the electrons can be transferred along the pathway of P → N → C → B according to the DFT calculations (Fig. 31b) [112]. Moreover, the smallest Δ*G* change of 0.16 eV further indicated BPCN was more favorable in the absorption and desorption processes of active H*, and thus HER performance (Fig. 31c). The femtosecond transient absorption spectra (fs-TAS) were employed to reveal the deep charge transfer dynamics. Specifically, for pristine BCN, only a negative signal was observed in the range of 420–800 nm, mainly due to the stimulated emission (SE, Fig. 31d–e). However, for BPCN, both negative signal and positive signals were seen in the range of 420–640 and 640–800 nm, respectively (Fig. 31g–h). The strongest positive absorption band of BPCN was ascribed to the excited states absorption (ESA) induced by the photogenerated electrons, indicating the fast charge excitation and separation processes. The authors continue to perform the kinetic decay curves to identify the lifetime species after excitation. According to the decay curves at 540 nm in Fig. 31f, the corresponding shortest fast-trapping component *τ*_1_ of 2.16 ps for BPCN indicated the shallowest defect states compared to those of CN, B-doped g-C_3_N_4_ (BCN) and P-doped g-C_3_N_4_ (PCN). As for the 750 nm decay curves, CN didn’t exist due to its ignorable positive signal. Interestingly, the authors observed a shortest *τ*_1_ of 0.3 ps and longest *τ*_2_ of 31 ps for BPCN (Fig. 31i), indicating its shallowest defect traps and longest charge separation lifetime due to the synergistic effect of the electron-rich P and electron-deficient B. Along with the optimized electronic band structure with smallest bandgap of 2.46 eV and electron transfer pathway, BPCN exhibited a superior photocatalytic HER rate of 4579 μmol h^−1^ g^−1^.

**Fig. 32:**
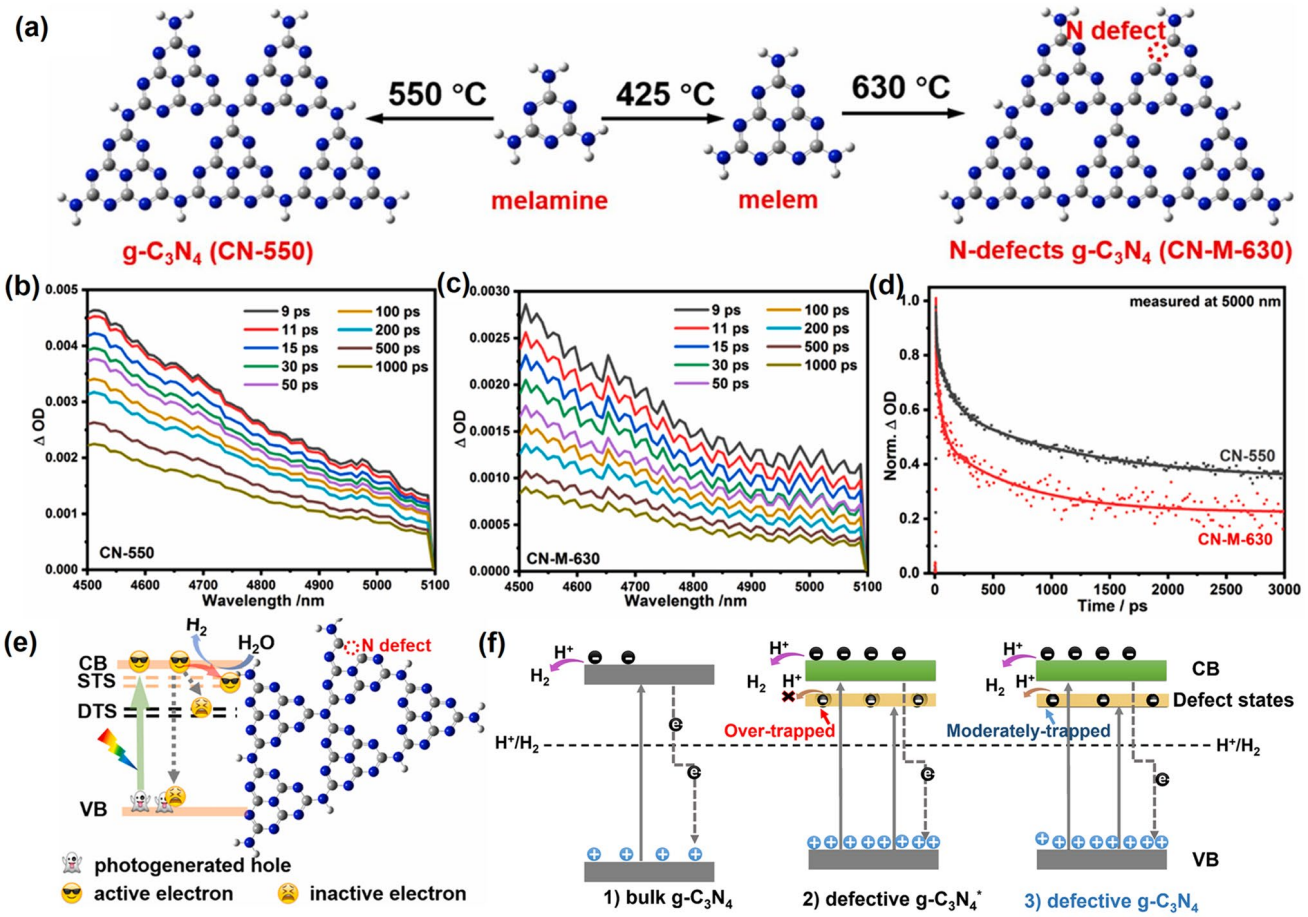
Defect control of defect states. **a** Schematic illustration for the fabrication of CN-550 and CN-M-630; MIR fs-TAS for **b** CN-550 and **c** CN-M-630; **d** MIR fs-TA decay kinetics; **e** scheme of the photocatalytic mechanism of CN-M-630 (STS: shallow trap states; DTS: deep trap states) [144]. Copyright 2022, Elsevier.** f** Electron-trapping ability of defect states with different positions [91]. Copyright 2023, Wiley–VCH

Zhu’s group proposed more direct evidence probing the charge dynamics in the trap states for N vacant g-C_3_N_4_ via the midinfrared femtosecond transient absorption spectroscopy (MIR fs-TAS) [144]. Two control samples, bulk CN-550 obtained at 550 °C with deep trap states and mesoporous N vacant g-C_3_N_4_ obtained at 630 °C with shallow trap states, were employed in this case (Fig. 32a). According to MIR fs-TAS, both samples showed the presence of trapped electrons as their relatively strong absorption bands from 4500 to 5100 nm (Fig. 32b-c). Their MIR decay kinetics curves reflected that CN-M-630 had much shorter lifetimes of *τ*_1_ and *τ*_2_ than CN-550, suggesting the relaxation of electrons from CB to more shallow trap states (Fig. 32d). A similar phenomenon was observed under visible light fs TA decay curves. In addition, the CN-M-630 displayed a prolonged longer lifetime *τ*_3_, indicating a slow decay of the recombination process in the presence of hole-trapping solvent (methanol, Me-OH). To further reveal the charge transfer dynamics, the time-resolved PL spectrum was performed, where *τ*_1_ (short lifetime) and *τ*_2_ (long lifetime) are assigned to the radiative and non-radiative decay of photocarriers from CB/defect states to VB, respectively. The CN-M-630 showed both shorter *τ*_1_ (1.33 vs. 2.59 ns) and *τ*_2_ (8.70 vs. 14.17 ns) with a decreased contribution of *τ*_1_ (51.0–49.4%), which demonstrated its lower quantity of quick recombined photocarrier and enhanced charge separation and transfer. Based on the above fs-TAS analysis, one can infer the shallow trap states in CN-M-630 enabled not only a fast charge separation and transfer process but also a suppressed photocarrier recombination, which is good for photocatalytic activity. While the deep trap states in CN-550 revealed a sluggish photocarrier transport and severe charge recombination process (Fig. 32e). Inspired by this work, our group realized a precise defect control on g-C_3_N_4_ with shallow defect states toward enhanced HER performance [91]. Specifically, the S dopants and N vacancies were simultaneously introduced into hollow g-C_3_N_4_ prisms via a dual-solvent-assisted synthetic strategy. By adding the ethylene glycol solvent into precursor formation and molten sulfur solvent into the pyrolysis process, the defective g-C_3_N_4_ exhibited a moderate concentration of N vacancy and a high S-doping level. This has been demonstrated to be effective to induce shallow defect states, which enabled both a promoted solar harvesting ability and a moderate electron-trapping ability to avoid photocarrier recombination (Fig. 32f). As a result, the resultant defective g-C_3_N_4_ displayed a superior HER rate of 4219.9 μmol g^−1^ h^−1^, which was 29.1-fold higher than unmodified g-C_3_N_4_.

**Fig. 33:**
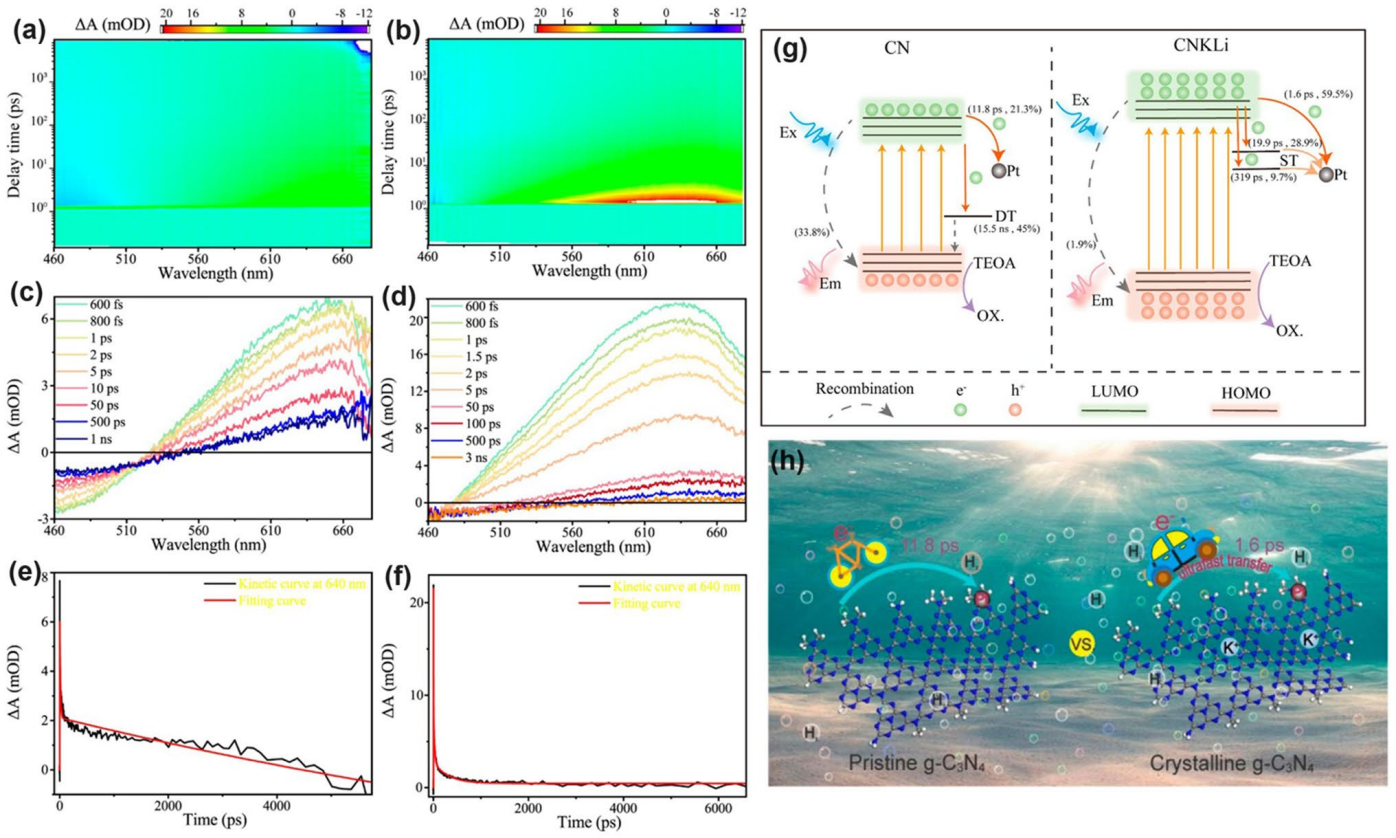
Defect control of defect states. **a, b** Pseudocolor fs-TAS of 2 wt% Pt-deposited CN and CNKLi in 10 vol % TEOA aqueous solution under the pump excitation of 350 nm; **c, d** fs-TAS at different delay times of 2 wt% Pt-deposited CN and CNKLi in 10 vol% TEOA aqueous solution; **e, f** Corresponding kinetic decay and fitting curves at 640 nm of 2 wt% Pt-deposited CN and CNKLi in 10 vol% TEOA aqueous solution; **g** Schematic illustration of the proposed charge carrier dynamics of CN and CNKLi (*E*_*x*_ stands for excitation; *E*_m_ stands for emission; DT stands for deep-trapped states, whereas ST is representative of shallowly trapped states); **h** schematic illustration of ultra-fast charge transfer in crystalline CNKLi than pristine one [308]. Copyright 2022, American Chemical Society

It is very exciting to learn that the fs-TAS is a powerful tool to reveal the photocarrier transfer kinetics so that we can glimpse an insight into the real defect behavior. It has been discussed above that the crystalline PHI-CN is a good photocatalyst HER catalyst owing to its weaker interlayered distance and ordered atomic arrangement [106]. Recently, excellent work on detecting the electron's lifetime in defect states has been contributed by Ye et al. by analyzing the fs-TAS spectrums of crystalline g-C_3_N_4_ obtained in the presence of KCl/LiCl mixture (CNKLi) and pristine g-C_3_N_4_ (CN) [308]. To simulate the real photocatalytic environment, the photocatalyst was deposited with 2 wt% Pt in 10% TEOA solution. As shown in Fig. 33a–d, CNKLi exhibited a negligible simulated emission, indicating the fast charge carrier separation process. In addition, the signal at around 640 nm of CNKLi (Fig. 33d) was significantly stronger than CN (Fig. 33c), further suggesting the efficient absorption of photogenerated charge carriers. The authors continue to perform the kinetic decay and fitting curves at 640 nm to identify the lifetime species after excitation. According to the decay curves in Fig. 33e–f, the corresponding short-lived and long-lived spectral component (*τ*_1_–*τ*_4_) can be obtained (Fig. 33g). The sluggish photocarrier transfer and severe recombination progress of CN was evidenced by the high 21.7% ratio of short-lived *τ*_1_ of 11.8 ps, whereas for CNKLi the *τ*_1_ value dramatically reduced to 1.6 ps of 59.5%, indicating an ultra-fast charge generation and transfer process due to the high PHI crystallinity. Different from the long-lived *τ*_2_ of 15.5 ns indicating severe photocarrier recombination of CN due to the deep-localized states, CNKLi exhibited short *τ*_2_ and *τ*_3_ in the ps scale, suggesting the formation of shallow defect states. In this case, the crystalline g-C_3_N_4_ with shallow defect states delivered the accelerated charge transfer kinetics via the advanced experimental fs-TA characterization, of which the photocatalytic activity would be boosted (Fig. 33h). The above works provide us with new insight into the charge transfer dynamics in defective trap states by more convincing data rather than the qualitative results.

## Conclusions and Outlook

**Fig. 34:**
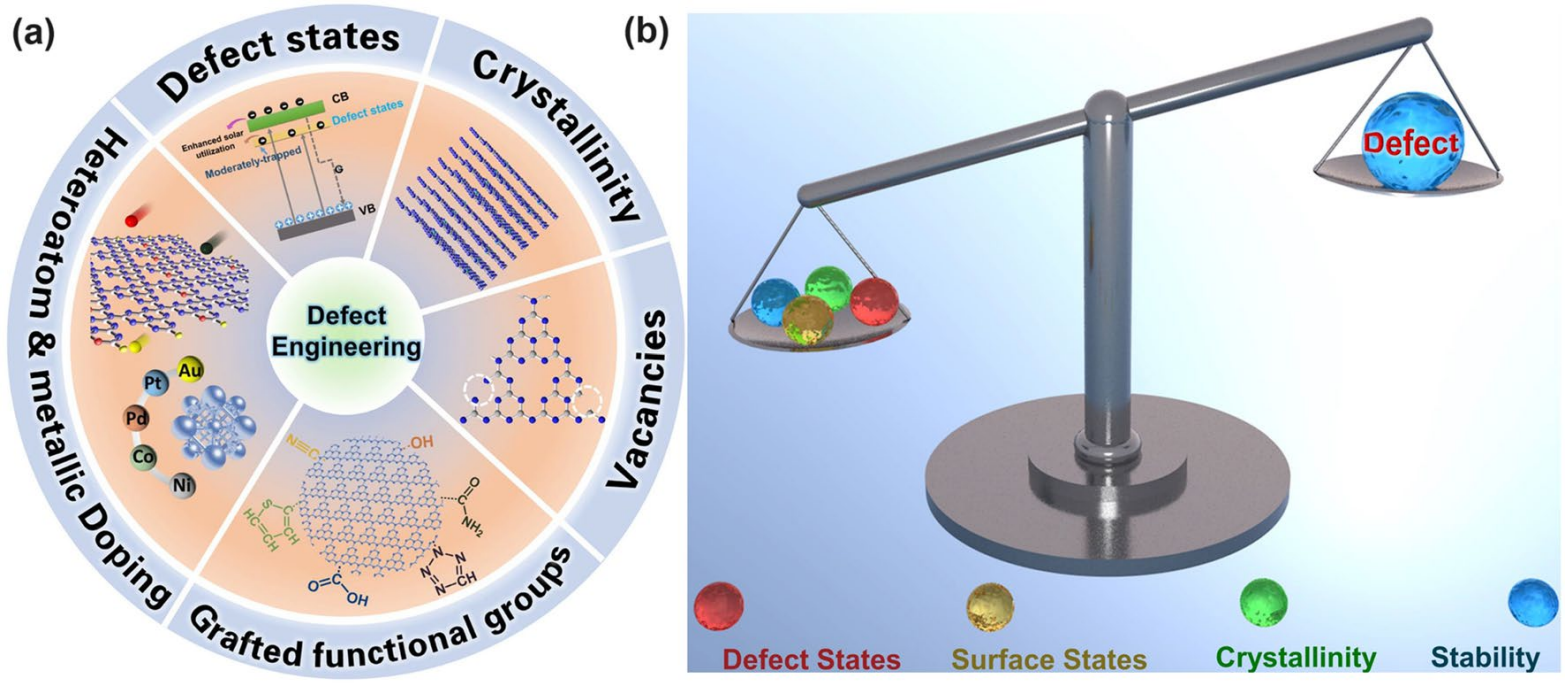
**a** Review summary; **b** defect design for future g-C3N4-based photocatalysis

Over the past 10 years, enormous attribution has been devoted to the defect-engineered g-C_3_N_4_ to boost its solar utilization on light harvesting and charge transfer kinetics by optimizing the electronic structure, electronic conductivity, and electronic polarization. We highlight the regulation strategies of vacancy creation, impurities doping by hetero-atoms and metallic atoms, defect modification of grafted functional groups, and crystallinity control. Despite great advancements being made, there is still space for future breakthroughs in the research direction of defect-engineered g-C_3_N_4_ in the following aspects (Fig. 34):

1. Further regulation on defect-associated energy levels to maximize the solar utilization, referring to shallow defect states and non-deterioration on surface states, preventing the traps from being the recombination centers. More importantly, the photocarrier transfer dynamics in defect states are meaningful to the deep understanding of defect creation, which needed to be characterized by more advanced optical techniques (fs-femtosecond transient absorption spectroscopy) or electrochemical method (EIS analysis).
2. Discreet manipulation of defect concentration to maintain the crystallinity of g-C_3_N_4_ at a reasonable level. As is known, there is a trade-off between defect engineering and crystallinity. The high crystallinity of PHI-based g-C_3_N_4_ enables a better in-plane and interlayer charge transfer due to the reduced hanging bonds induced by the defects. Thus, the balance between defects and crystallinity of g-C_3_N_4_ should be paid more attention.
3. Defect stability should be given more emphasis in future g-C_3_N_4_-based studies. Due to the long-term solar irradiation, the more inert bulk g-C_3_N_4_ itself would inevitably suffer from photocatalytic activity degradation. The vacancies, dopants, functional groups, and single/dual atoms might go through a more complex structure change, which needs to be detected via more advanced real-time technologies.
4. A precise understanding of each defect type in multiple-defect-modified g-C_3_N_4_ needs to be specified. The coupling of different defect types in g-C_3_N_4_ is common and the benefits for performance improvement are obvious. However, it is difficult to use the control variables strategy to fabricate the target defective g-C_3_N_4_ with a single defect or random mixing of defect types, making it hard to figure out which type/types are the most important. Building a precise calculated model as close to its experiment result to simulate the reaction process and catch a glimpse of real defect mechanism is a fantastic but challenging work, which deserves more research attention.

To this end, for a better future defect “customization” on g-C_3_N_4_, researchers need to fabricate the target g-C_3_N_4_ via a precise control on both defect type and concentration in the experiment along with the guidance of theoretical calculations. More importantly, the desired “customization” goal must obey basic principles: (i) tunable electronic band structure with designed CBM and VBM for a required photocatalytic activity, giving sufficient redox driving force; (ii) shallow defect states and optimized surface states to avoid the severe photocarrier recombination in both bulk phase and surface, maximizing the benefits of defects; (iii) optimized crystallinity with appropriate interlayered interaction and good balance with defects to guarantee the fast photocarrier transfer pathway, accelerating the redox kinetics; (iv) robust stability to maintain a high activity producing fuels, removing pollutants, and showing great potential for large-scale use.

In summary, we review the background and research history with significant progress, challenges, and corresponding solutions of defect-engineered g-C_3_N_4_ toward enhanced solar utilization on various applications. In addition, recent inspiring work on tracing the charge dynamics of trapping states is also emphasized. Furthermore, future design strategies for more effective defective g-C_3_N_4_ have also been proposed. We believe with synergetic efforts on defect controls and advanced characterization techniques, more breakthroughs in highly efficient g-C_3_N_4_-based photocatalysts in various solar applications can be achieved.
